# Waist circumference-years and cancer risk: a prospective study of the association and comparison of predictive performance with waist circumference and body mass index

**DOI:** 10.1038/s41416-024-02860-y

**Published:** 2024-10-04

**Authors:** Nadin Hawwash, Matthew Sperrin, Glen P. Martin, Corinne E. Joshu, Roberta Florido, Elizabeth A. Platz, Andrew G. Renehan

**Affiliations:** 1 Division of Cancer Sciences, School of Medical Sciences, Faculty of Biology, Medicine and Health, University of Manchester, Manchester, UK; 2 Cancer Research UK Manchester Cancer Research Centre, Manchester, UK; 3 Centre for Health Informatics, Division of Informatics, Imaging and Data Sciences, School of Health Sciences, Faculty of Biology, Medicine and Health, University of Manchester, Manchester, UK; 4 Department of Epidemiology, Johns Hopkins Bloomberg School of Public Health, Baltimore, MD USA; 5 Sidney Kimmel Comprehensive Cancer Center at Johns Hopkins, Baltimore, MD USA; 6 Johns Hopkins University School of Medicine, Department of Medicine, Division of Cardiology, Baltimore, MD USA; 7 Department of Medicine, Division of Cardiology, University of Utah, Salt Lake City, UT USA; 8 National Institute for Health Research (NIHR) Manchester Biomedical Research Centre, Manchester, UK

**Keywords:** Cancer epidemiology, Cancer prevention

## Abstract

**Background:**

Associations of waist circumferences (WC) and body mass index (BMI) measured once or over time, with cancer incidence were studied. WC is associated with some cancers independent of BMI. Analyses of cumulative central adiposity and cancer are lacking. We investigated associations between waist circumference-years, incorporating exposure time to WC ≥ 102 cm in men or ≥88 cm in women, and cancer, and compared this with single WC or BMI.

**Methods:**

Serial WC measurements taken over 9 years in the prospective Atherosclerosis Risk in Communities Study (ARIC) predicted yearly WC. Cox proportional hazards regression estimated hazard ratios (HRs) of cancer incidence for waist circumference-years, WC or BMI, measured in Visit 4. Harrell’s C-statistic quantified metric predictive performances.

**Results:**

10,172 participants were followed up from Visit 4 for cancer over a median 13.7 for men and 15.8 years for women. For obesity-related cancers, HRs per standard deviation waist circumference-years were 1.14 (95%CI:1.04,1.25) and 1.19 (95%CI:1.12,1.27), respectively. Differences in metric predictive performances were marginal.

**Discussion:**

This is the first study to identify positive associations between waist circumference-years and cancer. Waist circumference-years did not provide additional information on cancer risk beyond that of WC and BMI. BMI is routinely measured in clinic so it may be preferred over WC.

## Introduction

There is sufficient strength of evidence of an association between body mass index (BMI) and at least thirteen cancer sites; however, these findings are mainly based on a single BMI measure [1]. BMI is calculated by dividing the weight of an individual in kilograms by their height in metres squared. An alternative parameter used to quantify excess adiposity is waist circumference (WC). The 2020 Consensus Statement by the International Atherosclerosis Society and International Chair on Cardiometabolic Risk working group supported the inclusion of WC measures in clinical practice. Therefore, it is important to explore WC as a measure of excess adiposity to identify its potential relevance in cancer epidemiology and prevention [2].

WC is a measure of excess adiposity around the abdomen commonly taken using a tape measure. BMI cannot measure the proportion and distribution of adipose tissue [3]. For a BMI of 25 kg/m^2^, Janssen et al. found WC measures have increased by 1.10 cm in men and 4.90 cm in women over 30 years (1981 to 2007-2009) [4]. BMI is limited in capturing central adiposity, which correlates more with cardiometabolic risk [2]. Associations between WC and cancer risk were previously explored to identify whether central adiposity correlates more with cancer risk [5–15]. WC is associated with some cancers such as colon, post-menopausal breast, endometrial and pancreatic cancer independent of BMI [5]. Most studies were based on self-reported WC, which were found to be reasonably accurate indicators of excess adiposity [16]. A cohort study of 22.9 million Koreans found positive associations for measured WC with cancer sites before and after BMI adjustment; increased risk was found for colorectal, gastric, hepatobiliary, kidney, thyroid, brain, and lymphoma, with little change in association after adjusting for BMI [15]. Underlying mechanisms that may explain differential associations of BMI and WC metrics with incident cancers include inflammatory and metabolic cytokines and insulin resistance, which is strongly influenced by WC [17–19].

We previously explored associations between overweight-years (a metric incorporating exposure time to BMI ≥ 25 kg/m^2^) and cancer incidence [20]. What has yet to be explored in the cancer literature, is whether a metric that combines the cumulative degree of excess WC with the duration of excess WC exposure, known as waist circumference-years, has a stronger association with cancer (especially for cancer sites where WC is concluded to be associated with independent of BMI). A more careful definition of exposure to adiposity through the incorporation of cumulative degree and duration of exposure to excess central adiposity over time may be a more valid assessment of the role of excess adiposity as a cancer risk factor given the underlying biological mechanisms involved in the obesity-cancer link. Thus, the aims of this study were to (1) calculate the association between waist circumference-years and total, obesity-related and non-obesity-related cancer incidence, (2) estimate the association between cumulative degree or duration of excess WC over adulthood with cancer incidence, (3) compare the predictive performance of waist circumference-years with single WC and BMI measures and (4) compare the predictive performance of cumulative degree and cumulative duration of excess WC with cancer incidence, in a prospective cohort study of men and women who are Black and White. This study is part of a larger research project—the ABACus 2 consortium project [21].

## Methods

### Study population and design

The Atherosclerosis Risk in Communities (ARIC) study (RRID:SCR_021769) is a prospective cohort study of around 16,000 participants from four communities in the United States, namely Forsyth County in North Carolina, Washington County in Maryland, Jackson in Mississippi and suburbs of Minneapolis in Minnesota which were primarily followed up from recruitment in 1987–1989 to investigate causes and clinical outcomes of cardiovascular disease [22]. Participants were followed up for cancer until December 31, 2015. 99% of the total ARIC cohort were linkable to cancer registry data [23]. ARIC study participants were examined at Visit 1, with an average age 44 years, and then at three-year intervals for three more Visits. After 15 years, participants attended Visit 5 (2011-2013), Visit 6 (2016-2017), Visit 7 (2018-2019), Visit 8 (2019-2020) and Visit 9 (2021-2022) [22].

We conducted a prospective cohort analysis using Visit 4 as the baseline. Participants included (i) had no past or current diagnosis of cancer at Visit 4 [24], (ii) had WC and BMI measured at Visit 4 given both are exposures of interest and (iii) had at least 2 WC measurements before baseline to calculate waist circumference-years. We excluded participants who self-reported being a race other than Black or White.

### Exposure

Measures of WC from each of Visits 1 (1987–1989) through 4 (1997–1999) were used. WC was measured by trained technicians by horizontally applying a tape measure at the level of the umbilicus. Participants fasted overnight before Visits and wear scrub attire whilst measurements were taken [25]. WC was measured in centimetres. Trained personnel measured height and weight following a standardised protocol from which BMI was calculated by dividing the weight (kg) by the square of the height (m^2^) [25]. WC was quantified in two ways, (i) waist circumference-years, which accumulates time experiencing WC ≥ 102 cm in men or ≥88 cm in women as a fixed variable at Visit 4 (baseline); (Fig. S1), (ii) a single WC measure at Visit 4 (baseline), (iii) cumulative degree of excess WC which was the sum of WC exposures each year in excess (WC minus 102 cm in men) or (WC minus 88 cm) in women and (iv) cumulative duration of excess WC which was the number of years with a WC ≥ 102 cm in men or ≥88 cm in women. If a participant’s WC was no longer in excess i.e., the degree of excess WC was 0, then the duration of exposure to excess WC would be 0 and the cumulative waist circumference-years exposure, cumulative WC degree and cumulative WC duration measures would remain constant until their WC exceeds the excess WC threshold again. For comparing predictive performance of WC measures to BMI, we used a single BMI measure taken at Visit 4.

### Outcomes

Cancer diagnoses were the primary outcomes and ascertained through the linkage with four state cancer registries, hospital discharge summaries and medical records [23]. Secondary outcomes were obesity-related cancers (colorectal, gastric, oesophageal, thyroid, kidney, liver, pancreatic, multiple myeloma, gallbladder, meningioma, breast, endometrial and ovarian cancer) and non-obesity related cancers (the remaining cancer sites). We analysed cancer sites that fulfilled the criteria of having at least 10 events per candidate predictor parameter (EPP) separately; these were colorectal, pancreas, lung, kidney, prostate and bladder and metastatic prostate cancer in men and colorectal, pancreatic, endometrial, ovarian, kidney, lung, and post-menopausal breast cancer in women [26].

### Covariates

Self-reported covariates at Visit 4 were used, including cigarette smoking as “ever smokers” and “never smokers”; hormone replacement therapy (HRT) in women only as “ever HRT users” and “never HRT users” and alcohol (ethanol g/day). Race was self-reported at Visit 1 of the ARIC cohort study and solely included “White” and “Black” categories.

### Statistical analysis

Participants were followed from Visit 4 and follow-up was censored at the date of a first primary cancer diagnosis, death, or administrative censoring, where the study observation period ended on 31 December 2015, whichever came first. Follow-up was censored at the date of the other cancer event(s) (e.g., for incident obesity-related cancer, follow-up was censored at the date of non-obesity related cancer), if the other cancer event(s) came first (Fig. S1). We landmarked the time-point of interest to the baseline at Visit 4, given waist circumference-years require repeated WC measures over adulthood to calculate its exposure as a time-fixed variable at baseline [27]. Visit 4 was chosen as the index date as Visit 3 of the ARIC cohort study would have limited the duration of the exposure period to 9 years and an index date at Visit 5 would have led to a significant exclusion of participants from the study given the drop in the number of participants present at Visit 5 and the study inclusion requirement of a baseline WC measurement at the index date [22].

Missing covariate data were assumed to be missing at random and handled by multiple imputation to create ten imputed datasets [28]. Variables contained within the predictor matrix to predict plausible values for missing variables were “cancer incidence”, “cancer age”, “HRT (women only)”, “alcohol”, “education”, “smoking” and “race”. The remaining analyses were performed separately on each of the 10 imputed datasets and findings were pooled using Rubin’s rules [28]. WC thresholds vary by sex. Men and women were analysed separately and thresholds of ≥102 cm in men and ≥88 cm in women first used by the National Institutes of Health were used [29].

WC was predicted per year over the exposure period, from the minimum age at Visit 1 till each participant’s age at Visit 4, using a mixed effects model that included a random intercept, a random slope, an interaction term by sex (factoring in sex-specific differences in WC) and a spline on age. Yearly WC measures were used to calculate waist circumference-years by multiplying the degree of WC, i.e., the number of units above the WC threshold (≥102 cm in men or ≥88 cm in women) by the duration of exposure to that degree of WC. Waist circumference-years was a time-fixed exposure at baseline calculated as the sum of the waist circumference-years exposure prior to Visit 4. Cumulative degree of excess WC was the sum of the number of units above the WC threshold from Visit 1 to 4. Cumulative duration of excess WC was the total number of years with a WC measure above the WC threshold from Visit 1 to 4. An example calculation of waist circumference-years is shown in Table S1. Single WC and BMI measures at Visit 4 were separate exposures.

Incidence rates of all cancers combined were calculated per 1000 person-years for 0, 1-100, and >100 cm-years. Hazard ratios (HRs) of all cancer, obesity-related and non-obesity-related cancer associations were obtained using Cox proportional hazards models which were age-adjusted and multivariable-adjusted for age at baseline, race, HRT use (in women), alcohol and cigarette smoking. Variables that violated proportional hazards assumption were stratified or the hazard ratio was allowed to vary by the variable that violated the assumption depending on whether they were categorical or continuous variables respectively. Cumulative excess WC degree and duration from Visit 1 to 4 adjusted for baseline WC were analysed separately for their association with cancer incidence. Harrell’s C-statistic identified how well waist circumference-years discriminated those with cancer from those without (where a higher C-statistic showed increased discrimination) either in a model also including or compared with a single WC and BMI measure. The C-statistic was corrected for in-sample optimism using 100 bootstrapped models. Additional analysis included further multivariable adjustment with smoking pack-years (a cumulative measure of smoking exposure). The main analysis was repeated for White and Black participants separately to identify any variations in obesity-cancer trends by race. Analysis of interactions and stratification by race, smoking and HRT per SD waist circumference-years, single WC and single BMI was undertaken to identify variation in related cancer risks between potential effect modifier subgroups across all cancer subgroups and cancer sites explored.

### Sensitivity analysis

Non-obesity-related cancers were analysed, (i) overall and (ii) excluding lung and prostate in men given both cancers make up a large proportion of this subgroup and lung cancer associations may be subject to residual confounding by smoking and prostate cancers have a common inverse association possibly due to cause and/or detection bias [30, 31]. Two main sensitivity analyses were undertaken. First, the analysis was repeated using lower WC thresholds to adjust for any potential excess risk of adiposity-related cancer associations below the threshold. In line with prior literature, lower WC thresholds of 90 cm for men and 76 cm for women were used [32]. Second, the main analysis was repeated including participants with at least 1 WC measurement to identify any selection bias in the main analysis through selection of those with at least 3 WC measurements.

High Performance Computing was used. R 4.1.2 (RRID:SCR_001905), and the following packages purr [33], lme4 [34], magrittr [35], tidyverse [36], haven [37], survival [38], interactions [39], rms [40], ggplot2 [41], survminer [42], lubridate [43], ggpubr [44], lattice [45], arsenal [46], Hmisc [47] kableExtra [48], gtsummary [49], splines [50], Ecdat [51], were used. R markdown helped combine results into tables [52]. Strengthening the Reporting of Observational studies in Epidemiology (STROBE) was used (Table S2) [53].

## Results

**Fig. 1:**
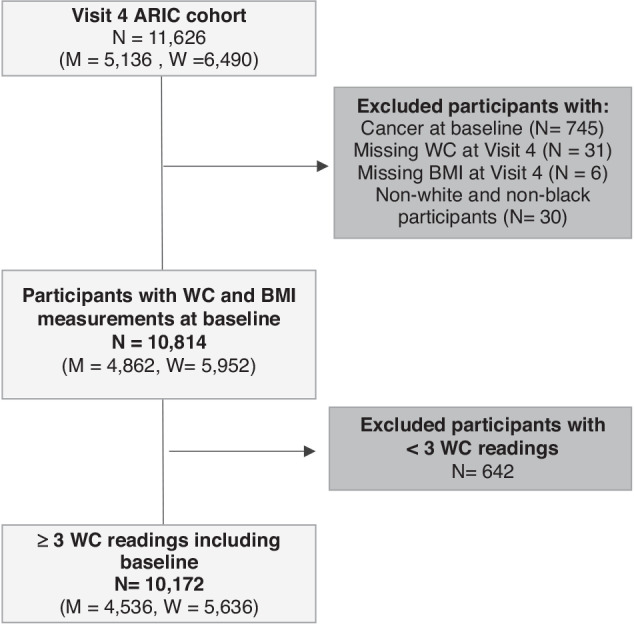
Flow diagram of individuals in the ARIC cohort. Excluded individuals (dark grey). Included individuals (light grey). Waist circumference-related exclusion criteria were observational level exclusions but led to individual exclusions if all waist circumference readings were outside the range. ARIC atherosclerosis risk in communities (Study), WC waist circumference, N number of participants, BMI body mass index.

At Visit 4 (baseline) of the ARIC cohort, there were 11,626 participants. Those with cancers prior to and at baseline (745 participants), missing baseline WC (31 participants) and baseline BMI (6 participants) measurements were excluded (Fig. 1). 30 participants not of White or Black race were excluded due to the small numbers in other racial groups and those with less than 3 WC measurements were also excluded (642 participants). 10,172 participants were included in this study (4536 men and 5636 women) and followed up for 16.7 years (IQR 11.5–21.9) and 18.1 years (IQR 15.7–20.6) respectively (Fig. 1, Table S3). During follow-up, 1514 men and 1239 women were diagnosed with a first primary cancer. Table 1 describes the baseline characteristics. Men and women included had a mean age of 63 years (SD 6) and 62 years (SD 6) and a mean WC at baseline of 103 cm (SD 12) and 101 cm (SD 16) respectively (Table 1). 19% of men and 27% of women were Black. On average, waist circumference-years exposure was 32 cm-years (SD 58) for men and 101 cm-years (SD 113) for women (Table S3). Incidence rates of all cancers combined were highest for >100 cm-years than <100 cm-years groups including those of White or Black race, ever smokers or never smokers and women that were ever or never HRT users (Table S4).

**Table 1:** Characteristics^a^ of the analytic cohort at Visit 4, ARIC.

Characteristic	Men			Women		
	Overall *N* = 4536	≤ Median WC (cm) *N* = 2321	> Median WC (cm) *N* = 2215	Overall *N* = 5636	≤ Median WC (cm) *N* = 2827	> Median WC (cm) *N* = 2809
Age at baseline, years, mean (SD)	62.9 (5.7)	63.0 (5.7)	62.8 (5.6)	62.3 (5.6)	62.1 (5.6)	62.5 (5.5)
Height^a^, metres, mean (SD)	1.8 (0.1)	1.7 (0.1)	1.8 (0.1)	1.6 (0.1)	1.6 (0.1)	1.6 (0.1)
BMI at baseline^a^, kg/m^2^, mean (SD)	28.5 (4.5)	25.5 (2.5)	31.7 (3.9)	29.2 (6.4)	24.8 (3.3)	33.6 (5.7)
Mean WC at baseline^a^, cm, mean (SD)	103.0 (12.0)	94.0 (6.0)	112.0 (9.0)	101.0 (16.0)	88.0 (8.0)	114.0 (12.0)
**Race,** ***n*** **(%)**						
White	3685 (81.2)	1820 (78.4)	1865 (84.2)	4121 (73.1)	2261 (80.0)	1860 (66.2)
Black	851 (18.8)	501 (21.6)	350 (15.8)	1515 (26.9)	566 (20.0)	949 (33.8)
**Smoking,** ***n*** **(%)**						
Ever	3266 (72.0)	1621 (69.8)	1645 (74.2)	2648 (47.0)	1377 (48.7)	1271 (45.2)
Never	1238 (27.2)	682 (29.3)	556 (25.1)	2949 (52.3)	1432 (50.1)	1516 (54.0)
Missing	32 (0.7)	18 (0.7)	14 (0.6)	39 (0.7)	18 (0.6)	21 (0.7)
Alcohol consumption, units of alcohol^a^ per week, mean (SD)	3.9 (7.8)	3.9 (7.6)	4 (8.1)	1.2 (3.2)	1.6 (3.6)	0.8 (2.7)
Missing	39 (0.8)	21 (0.9)	18 (0.8)	41 (0.7)	18 (0.6)	23 (0.8)
**HRT,** ***n*** **(%)**						
Ever				2045 (36.3)	1145 (40.5)	900 (32.0)
Never				2071 (36.7)	913 (32.3)	1158 (41.2)
Missing				1520 (27.0)	769 (27.2)	751 (26.7)
**Education,** ***n*** **(%)**						
Graduate or professional school	621 (13.7)	355 (15.3)	266 (12.0)	501 (8.9)	281 (9.9)	220 (7.8)
At least some college	1337 (29.4)	654 (28.1)	683 (30.8)	1438 (25.5)	856 (30.3)	582 (20.7)
Vocational school	438 (9.7)	217 (9.3)	221 (10.0)	447 (7.9)	233 (8.2)	214 (7.6)
Completed high school	1253 (27.6)	625 (26.9)	628 (28.3)	2140 (38.0)	1049 (37.1)	1091 (38.8)
High school but no degree	492 (10.8)	266 (11.5)	226 (10.2)	750 (13.3)	293 (10.4)	457 (16.3)
Grade school or 0 years education	385 (8.5)	199 (8.6)	186 (8.4)	353 (6.3)	112 (4.0)	241 (8.6)
Missing	10 (0.2)	5 (0.2)	5 (0.2)	7 (0.1)	3 (0.1)	4 (0.1)

*N* number of participants, *BMI* body mass index, *WC* waist circumference, *HRT* hormone replacement therapy.

^a^Mean (SD); *n* ().

Values in parentheses are percentages unless otherwise stated.

Covariates are from the start of follow-up at Visit 4 except race and education which were collected at Visit 1.

Baseline refers to Visit 4.

BMI is calculated from weight and height that was measured in a standard way by trained technicians.

### Associations of waist circumference-years, BMI, WC with cancer

Significant positive associations were identified per SD increment in waist circumference-years and incident obesity-related cancers in men and women with multivariable-adjusted HRs of 1.14 (95% CI:1.04,1.25) and 1.19 (95% CI:1.12,1.27) respectively (Table 2). In men, non-significant positive associations were found across all metrics for non-obesity-related cancers; however, on analysis of the non-obesity-related cancer subgroup without prostate and lung cancers, significant positive associations were found across all metrics - waist circumference-years, single WC and single BMI—with HRs of 1.15 (95% CI:1.06,1.24), 1.12 (95% CI:1.02,1.23) and 1.11 (95% CI:1.01,1.22) respectively (Table 2**)**. Significant positive associations were found across all 3 metrics for colorectal cancer in men with HRs of 1.22 (95% CI:1.09,1.37), 1.26 (95% CI:1.07,1.49) and 1.26 (95% CI:1.07,1.47) respectively. There was a non-significant positive association for lung cancer in men per SD increment in waist circumference-years of 1.02 (95% CI:0.90,1.16) per SD increment in waist circumference-years and a significant inverse association per SD increment in BMI of 0.77 (95% CI: 0.66,0.89) (Table 2).

**Table 2:** Hazard ratio of cancers per standard deviation of waist circumference-years at Visit 4 and BMI at Visit 4, ARIC.

Outcomes	Cancers	Waist circumference-years (per SD)		Single WC (per SD)		Single BMI (per SD)	
		Age-adjusted HR (95% CI)	MV-adjusted* HR (95% CI)	Age-adjusted HR (95% CI)	MV-adjusted* HR (95% CI)	Age-adjusted HR (95% CI)	MV-adjusted* HR (95% CI)
		Men					
All Cancers	1514	1.08 (1.03,1.12)	1.07 (1.03,1.12)	1.04 (0.99,1.09)	1.04 (0.99,1.10)	1.03 (0.98,1.08)	1.02 (0.97,1.08)
OR-cancers	306	1.14 (1.04,1.25)	1.14 (1.04,1.25)	1.13 (1.02,1.27)	1.14 (1.02,1.27)	1.13 (1.02,1.26)	1.13 (1.01,1.26)
NOR-cancers	1208	1.06 (1.00,1.11)	1.06 (1.00,1.11)	1.02 (0.96,1.07)	1.02 (0.96,1.08)	1.00 (0.94,1.06)	1.00 (0.94,1.06)
NOR cancers excluding lung and prostate	426	1.15 (1.07,1.24)	1.15 (1.06,1.24)	1.14 (1.04,1.25)	1.12 (1.02,1.23)	1.11 (1.01,1.22)	1.11 (1.01,1.22)
**Specific cancer sites**							
Colorectal	126	1.23 (1.09,1.38)	1.22 (1.09,1.37)	1.25 (1.06,1.48)	1.26 (1.07,1.49)	1.27 (1.08,1.49)	1.26 (1.07,1.47)
Pancreas	45	1.04 (0.79,1.39)	1.05 (0.79,1.4)	1.11 (0.83,1.48)	1.13 (0.85,1.51)	1.16 (0.88,1.54)	1.16 (0.88,1.54)
Kidney	55	0.96 (0.72,1.29)	0.96 (0.72,1.28)	0.96 (0.74,1.26)	0.97 (0.74,1.27)	0.97 (0.74,1.27)	0.96 (0.73,1.27)
Bladder	53	1.13 (0.90,1.43)	1.12 (0.88,1.42)	1.21 (0.93,1.57)	1.17 (0.9,1.52)	1.17 (0.9,1.52)	1.16 (0.89,1.50)
Lung	211	1.04 (0.91,1.17)	1.02 (0.9,1.16)	0.89 (0.78,1.03)	0.87 (0.75,1.00)	0.77 (0.66,0.89)	0.77 (0.66,0.89)
Prostate	571	0.99 (0.91,1.07)	0.99 (0.92,1.08)	0.97 (0.90,1.06)	1.00 (0.92,1.09)	1.01 (0.93,1.10)	1.01 (0.93,1.09)
Metastatic Prostate	34	0.86 (0.56,1.31)	0.88 (0.58,1.32)	0.92 (0.65,1.30)	0.97 (0.69,1.35)	0.95 (0.68,1.35)	0.95 (0.68,1.34)
**Women**							
All Cancers	1239	1.10 (1.05,1.16)	1.12 (1.06,1.18)	1.13 (1.07,1.19)	1.15 (1.09,1.22)	1.12 (1.06,1.18)	1.15 (1.08,1.21)
OR-cancers	751	1.18 (1.11,1.26)	1.19 (1.12,1.27)	1.21 (1.13,1.29)	1.22 (1.13,1.31)	1.18 (1.11,1.27)	1.2 (1.12,1.29)
NOR-cancers	488	0.98 (0.90,1.07)	1.01 (0.92,1.11)	1.02 (0.93,1.11)	1.05 (0.96,1.15)	1.01 (0.92,1.10)	1.06 (0.97,1.16)
NOR cancers excluding lung	336	1.00 (0.90,1.11)	1.02 (0.92,1.14)	1.04 (0.93,1.16)	1.07 (0.96,1.19)	1.06 (0.95,1.18)	1.11 (0.99,1.24)
**Specific cancer sites**							
Colorectal	132	1.19 (1.03,1.38)	1.17 (1.01,1.37)	1.08 (0.91,1.28)	1.06 (0.89,1.26)	1.06 (0.89,1.25)	1.02 (0.86,1.22)
Pancreas	43	1.27 (1.00,1.61)	1.16 (0.90,1.50)	1.33 (1.00,1.77)	1.21 (0.90,1.62)	1.19 (0.90,1.58)	1.04 (0.76,1.40)
Kidney	41	1.32 (1.04,1.69)	1.31 (1.02,1.69)	1.47 (1.11,1.94)	1.46 (1.10,1.95)	1.43 (1.10,1.85)	1.42 (1.08,1.87)
Lung	152	0.95 (0.80,1.11)	0.98 (0.83,1.16)	0.97 (0.82,1.14)	1.00 (0.85,1.18)	0.89 (0.75,1.06)	0.96 (0.81,1.14)
Endometrial	76	1.60 (1.37,1.87)	1.62 (1.38,1.9)	1.80 (1.48,2.18)	1.85 (1.51,2.27)	1.67 (1.4,2)	1.78 (1.47,2.14)
Ovarian	40	0.93 (0.67,1.31)	1.05 (0.75,1.47)	1.00 (0.74,1.37)	1.12 (0.81,1.54)	0.91 (0.65,1.26)	1.05 (0.75,1.47)
Post-menopausal breast	357	1.09 (0.99,1.20)	1.11 (1.01,1.23)	1.15 (1.04,1.27)	1.18 (1.06,1.31)	1.14 (1.03,1.26)	1.17 (1.06,1.30)

*Multivariable adjustment for baseline age, race, alcohol, smoking and HRT (in women).

*OR* obesity-related, *NOR* non-obesity related, *CI* confidence interval, *HR* hazard ratio, *BMI* body mass index, *MV* multivariable, *WC* waist circumference.

In women, a significant positive association was found for colorectal cancer per SD waist circumference-years of 1.17 (95% CI:1.01,1.37) but positive non-significant HRs were found per SD WC and BMI measure with HRs of 1.06 (95% CI: 0.89,1.26) and 1.02 (95% CI:0.86,1.22) respectively (Table 2). For kidney cancer in women, there were significant positive associations across all 3 exposure metrics -waist circumference-years, single WC and single BMI—with HRs of 1.31 (95% CI:1.02,1.69), 1.46 (95% CI:1.10,1.95) and 1.42 (95% CI:1.08,1.87) respectively. For endometrial cancer, significant positive associations across all 3 exposure metrics were found with HRs of 1.62 (95% CI:1.28,1.90), 1.85 (95% CI:1.51,2.27) and 1.78 (95% CI:1.47,2.14). Positive associations across all 3 exposure metrics were found for post-menopausal breast cancer with HRs of 1.11 (95% CI:1.01,1.23), 1.18 (95% CI:1.06,1.31) and 1.17 (95% CI:1.06,1.30) (Table 2). Cox proportional hazards assumptions were not violated following the inclusion of time-varying by baseline age and stratification by race. Analysis per 100 waist circumference-years, per 5 cm WC per 5 kg/m^2^ BMI is shown in Table S5 and analysis per 10 cm increment in WC and per 10-year duration of excess WC is shown in Table S6. Little variance was found across the Akaike information criterion (AIC) of the metrics explored (Table S7).

Additional analysis found no interactions by race between waist circumference-years, single WC and BMI. Positive associations were found per SD waist circumference-years in White men, but inverse associations were found in Black men for all cancers (Table S9). A significant interaction was found by smoking per SD waist circumference-years and colorectal cancer in men: never smokers had a higher HR of association than ever smokers (Table S10). Further multivariable adjustment for smoking pack-years showed little variation in cancer risk per SD increment in waist circumference-years, single WC and single BMI (Table S11). Analysis by HRT use found an interaction only for endometrial cancer per SD waist circumference-years, single WC and BMI with higher HRs of association in never vs ever HRT users (Table S12).

### Association of cumulative excess waist circumference degree and duration with cancer

In men, for all cancers, a significant positive association was found for cumulative excess WC degree, but not for cumulative WC duration with HRs of 1.11 (95% CI:1.03,1.19) and 1.05 (95% CI:0.97,1.13) respectively (Table 3). For obesity-related cancers in men, positive but non-significant associations were found per SD increment in cumulative excess WC degree and duration; however, for non-obesity-related cancers, a significant positive association was found per SD increment in cumulative degree of excess WC, but the association was non-significant per SD increment in cumulative duration of excess WC with HRs of 1.11 (95 CI:1.02,1.20) and 1.05 (95% CI: 0.96,1.14) respectively. A similar pattern was found on exclusion of lung and prostate cancers from the non-obesity-related cancer subgroup with HRs of 1.17 (95% CI:1.03,1.32) and 1.15 (95% CI:1.00,1.32) respectively. For bladder cancer, men had significant associations per SD increment in the duration of cumulative excess WC but no evidence of an association per SD degree of cumulative excess WC with HRs of 1.88 (95% CI:1.3,2.72) and 1.00 (95% CI:0.66,1.52) respectively. For lung cancer, men only had significant associations per SD degree of cumulative excess WC and not for duration of cumulative excess WC with HRs of 1.31 (95% CI:1.08,1.58) and 1.18 (95% CI:0.98,1.43) respectively (Table 3).

**Table 3:** Hazard ratios of cancers per standard deviation cumulative excess waist circumference degree and duration at Visit 4, in ARIC.

Outcomes		Cumulative degree of waist circumference^a^ (per SD)		Cumulative duration of waist circumference^b^ (per SD)	
		Age-adjusted HR (95% CI)	MV-adjusted* HR (95% CI)	Age-adjusted HR (95% CI)	MV-adjusted* HR (95% CI)
	Cancers	Men			
All Cancers	1514	1.12 (1.04,1.20)	1.11 (1.03,1.19)	1.05 (0.98,1.14)	1.05 (0.97,1.13)
OR-cancers	306	1.13 (0.97,1.32)	1.12 (0.96,1.30)	1.05 (0.88,1.24)	1.05 (0.88,1.24)
NOR-cancers	1208	1.11 (1.03,1.20)	1.11 (1.02,1.20)	1.06 (0.97,1.15)	1.05 (0.96,1.14)
NOR cancers excluding lung and prostate	426	1.15 (1.01,1.3)	1.17 (1.03,1.32)	1.15 (1.00,1.32)	1.15 (1.00,1.32)
**Specific cancer sites**					
Colorectal	126	1.19 (0.97,1.47)	1.17 (0.95,1.44)	1.03 (0.80,1.32)	1.03 (0.80,1.32)
Pancreas	45	0.93 (0.58,1.49)	0.91 (0.56,1.48)	1.22 (0.79,1.89)	1.20 (0.78,1.86)
Kidney	55	0.98 (0.63,1.53)	0.97 (0.63,1.51)	0.89 (0.57,1.39)	0.90 (0.57,1.41)
Bladder	53	0.97 (0.64,1.47)	1.00 (0.66,1.52)	1.90 (1.31,2.76)	1.88 (1.30,2.72)
Lung	211	1.26 (1.06,1.50)	1.31 (1.08,1.58)	1.23 (1.01,1.49)	1.18 (0.98,1.43)
Prostate	571	1.01 (0.89,1.15)	0.98 (0.86,1.11)	0.92 (0.81,1.05)	0.92 (0.80,1.04)
Metastatic Prostate	34	0.83 (0.44,1.56)	0.79 (0.42,1.48)	1.09 (0.64,1.85)	1.07 (0.63,1.81)
**Women**					
All Cancers	1239	1.00 (0.90,1.11)	1.01 (0.91,1.12)	1.06 (0.97,1.15)	1.08 (0.99,1.18)
OR-cancers	751	1.07 (0.94,1.21)	1.07 (0.95,1.22)	1.13 (1.00,1.27)	1.14 (1.01,1.28)
NOR-cancers	488	0.89 (0.75,1.05)	0.91 (0.77,1.08)	0.98 (0.86,1.13)	1.02 (0.89,1.17)
NOR cancers excluding lung	336	0.88 (0.71,1.08)	0.90 (0.73,1.10)	0.97 (0.82,1.14)	0.99 (0.84,1.17)
**Specific cancer sites**					
Colorectal	132	1.46 (1.12,1.92)	1.45 (1.10,1.9)	1.58 (1.20,2.09)	1.57 (1.19,2.07)
Pancreas	43	1.10 (0.68,1.79)	1.05 (0.64,1.72)	1.27 (0.79,2.03)	1.21 (0.76,1.93)
Kidney	41	0.93 (0.54,1.58)	0.93 (0.54,1.60)	1.47 (0.86,2.52)	1.48 (0.87,2.54)
Lung	152	0.90 (0.66,1.23)	0.94 (0.69,1.28)	1.02 (0.80,1.30)	1.08 (0.85,1.38)
Endometrial	76	1.16 (0.81,1.65)	1.14 (0.80,1.62)	0.91 (0.61,1.35)	0.93 (0.62,1.38)
Ovarian	40	0.78 (0.40,1.52)	0.85 (0.44,1.64)	1.17 (0.71,1.93)	1.25 (0.75,2.08)
Post-menopausal breast	357	0.90 (0.74,1.10)	0.91 (0.75,1.11)	1.08 (0.92,1.28)	1.09 (0.93,1.29)

*Multivariable adjustment for baseline age, baseline WC, race, alcohol, smoking and HRT (in women).

^a^Degree of excess WC is the cumulative sum of the number of WC units ≥102 cm in men and ≥88 cm in women over the exposure period.

^b^Duration of excess WC is the cumulative sum of the duration of WC above the threshold over the exposure period.

*OR* obesity-related, *NOR* non-obesity related, *CI* confidence interval, *HR* hazard ratio, *BMI* body mass index, *MV* multivariable, *WC* waist circumference.

In women, for obesity-related cancers combined, a significant positive association was found per SD duration but not degree of cumulative excess WC with HRs of 1.14 (95% CI:1.01,1.28) and 1.07 (95% CI:0.95,1.22) respectively. For colorectal cancer in women, significant positive associations were found both per SD increment in cumulative degree and duration of excess WC with HRs of 1.45 (95% CI:1.1,1.9) and 1.57 (95% CI:1.19,2.07) respectively (Table 3). For kidney, lung, endometrial and ovarian and post-menopausal breast cancer in women, there were non-significant positive associations per SD increment in cumulative duration of excess WC unlike per SD increment in cumulative degree of excess WC where associations were non-significant and inverse (Table 3). However, for endometrial cancer, there was a non-significant positive association per SD cumulative degree unlike per SD cumulative duration of excess WC where duration had a non-significant inverse association with HRs of 1.14 (95% CI:0.8,1.62) and 0.93 (95% CI:0.62,1.38) respectively.

### Model performance characteristics

Overall marginal differences in the C-statistic were found across metrics - waist circumference-years, single WC and single BMI (Table 4; Table S8). For lung cancer in men, there was a significantly higher C-statistic for single BMI than single WC with C-statistics of 0.739 (95% CI:0.717,0.761) and 0.730 (95% CI:0.709,0.752) respectively and a difference of 0.009 (95% CI:0.001,0.017) (Table 4). On comparison of the predictive performance between cumulative degree and duration of overweight, differences were found in the predictive performance for bladder cancer in men which had a significantly higher C-statistic per cumulative duration vs degree of excess WC of 0.648 (95% CI:0.604,0.694) and 0.615 (95% CI:0.572,0.661), respectively with a difference of 0.053 (95% CI:0.004,0.102).

**Table 4:** Comparison of the waist circumference-years metrics, single waist circumference and body mass index using Harrell’s C-statistic, ARIC.

Characteristic	(a) Harrell’s C-statistic (95% CI)								
	WC-years	Single WC	Difference in c-statistic between single WC vs WC-years	Single BMI	Difference in c-statistic between single BMI vs WC-years	Difference in c-statistic between single BMI vs single WC	Cumulative degree of excess WC	Cumulative duration of excess WC	Difference in c-statistic between cumulative WC duration and WC degree
**Men**									
**All cancers**	0.587 (0.578,0.597)	0.585 (0.576,0.595)	–0.002 (-0.007,0.002)	0.584 (0.575,0.594)	–0.003 (-0.008,0.002)	–0.001 (–0.004,0.002)	0.588 (0.578,0.597)	0.585 (0.576,0.595)	–0.002 (–0.008,0.004)
**OR-cancers**	0.581 (0.562,0.601)	0.580 (0.560,0.599)	–0.002 (-0.013,0.010)	0.578 (0.558,0.598)	–0.004 (-0.015,0.008)	–0.002 (–0.011,0.007)	0.581 (0.562,0.602)	0.572 (0.553,0.592)	–0.009 (–0.023,0.004)
**NOR-cancers**	0.593 (0.582,0.604)	0.592 (0.582,0.603)	–0.001 (-0.011,0.009)	0.593 (0.582,0.603)	–0.001 (-0.010,0.009)	0.000 (–0.007,0.008)	0.593 (0.582,0.604)	0.592 (0.582,0.603)	–0.000 (–0.008,0.007)
**NOR-cancers excluding lung and prostate**	0.600 (0.583,0.619)	0.600 (0.583,0.618)	–0.000 (-0.008,0.007)	0.600 (0.582,0.618)	–0.001 (-0.008,0.007)	–0.000 (–0.005,0.004)	0.600 (0.583,0.619)	0.599 (0.581,0.617)	–0.001 (–0.010,0.007)
Colorectal	0.638 (0.607,0.670)	0.630 (0.600,0.662)	–0.007 (-0.025,0.010)	0.629 (0.599,0.661)	–0.009 (-0.028,0.011)	–0.001 (–0.014,0.011)	0.638 (0.607,0.670)	0.629 (0.598,0.661)	–0.009 (–0.029,0.011)
Pancreas	0.644 (0.596,0.696)	0.643 (0.595,0.695)	0.016 (-0.020,0.051)	0.644 (0.595,0.696)	0.027 (-0.016,0.070)	0.011 (–0.009,0.031)	0.644 (0.596,0.696)	0.641 (0.592,0.693)	0.032 (–0.018,0.084)
Kidney	0.655 (0.606,0.707)	0.657 (0.609,0.710)	–0.001 (-0.005,0.003)	0.653 (0.606,0.704)	–0.001 (-0.005,0.004)	0.001 (–0.003, 0.004)	0.655 (0.606,0.707)	0.707 (0.658,0.760)	–0.004 (–0.014,0.008)
Bladder	0.612 (0.569,0.657)	0.627 (0.585,0.672)	0.003 (-0.025, 0.030)	0.638 (0.596,0.684)	–0.007 (-0.029,0.025)	–0.005 (–0.019,0.010)	0.615 (0.572,0.661)	0.648 (0.604,0.694)	0.053 (0.004,0.102)
Lung	0.722 (0.701,0.743)	0.730 (0.709,0.752)	0.008 (-0.003,0.019)	0.739 (0.717,0.761)	0.017 (-0.000,0.034)	0.009 (0.001,0.017)	0.722 (0.701,0.743)	0.724 (0.703,0.745)	0.002 (–0.001,0.005)
Prostate	0.588 (0.573,0.603)	0.588 (0.574,0.603)	0.000 (-0.001,0.002)	0.588 (0.576,0.603)	0.000 (-0.002,0.002)	0.000 (–0.001,0.001)	0.588 (0.573,0.603)	0.588 (0.573,0.603)	0.000 (–0.003,0.003)
Metastatic Prostate	0.573 (0.525,0.626)	0.585 (0.534,0.640)	0.012 (-0.033,0.057)	0.588 (0.535,0.647)	0.015 (-0.047,0.077)	0.003 (–0.032,0.039)	0.573 (0.525,0.626)	0.552 (0.511,0.597)	–0.021 (–0.097,0.055)
**Women**									
**All cancers**	0.576 (0.565,0.586)	0.579 (0.568,0.590)	0.003 (–0.004,0.011)	0.578 (0.567,0.588)	0.002 (-0.007,0.011)	–0.001 (–0.010,0.007)	0.579 (0.569,0.590)	0.578 (0.568,0.589)	–0.001 (–0.014,0.011)
**OR-cancers**	0.577 (0.564,0.589)	0.574 (0.562,0.570)	–0.002 (–0.013,0.009)	0.568 (0.556,0.581)	–0.008 (-0.019,0.003)	–0.006 (–0.014,0.003)	0.577 (0.564,0.590)	0.575 (0.562,0.588)	–0.0022 (–0.016,0.012)
**NOR-cancers**	0.628 (0.611,0.644)	0.628 (0.612,0.645)	0.001 (-0.002,0.003)	0.629 (0.613,0.645)	0.001 (-0.002,0.005)	0.001 (–0.001,0.002)	0.628 (0.611,0.644)	0.629 (0.612,0.645)	0.001 (–0.002,0.004)
**NOR-cancers excluding lung**	0.578 (0.560,0.597)	0.582 (0.565,0.601)	0.005 (-0.003,0.012)	0.588 (0.571,0.607)	0.011 (-0.000,0.022)	0.006 (0.000,0.013)	0.5782 (0.560,0.597)	0.579 (0.561,0.598)	0.001 (–0.004,0.006)
Colorectal	0.618 (0.588,0.650)	0.590 (0.559,0.621)	–0.029 (–0.051, –0.006)	0.586 (0.556,0.618)	–0.032 (–0.061,–0.004)	–0.004 (–0.015,0.008)	0.617 (0.587,0.649)	0.617 (0.586,0.650)	–0.001 (–0.028,0.027)
Pancreas	0.622 (0.565,0.685)	0.625 (0.568,0.688)	0.003 (–0.015,0.021)	0.614 (0.559,0.674)	–0.008 (–0.041,0.025)	–0.011 (–0.047,0.025)	0.622 (0.565,0.685)	0.628 (0.571,0.690)	0.006 (–0.036,0.048)
Kidney	0.653 (0.596,0.715)	0.661 (0.601,0.727)	0.008 (–0.023,0.040)	0.656 (0.596,0.719)	0.002 (–0.015,0.019)	–0.007 (–0.034,0.021)	0.654 (0.597,0.716)	0.653 (0.591,0.723)	–0.001 (–0.061,0.060)
Lung	0.761 (0.733,0.789)	0.761 (0.733,0.789)	0.000 (–0.001,0.001)	0.761 (0.733,0.789)	0.000 (–0.001,0.002)	–0.000 (–0.002,0.002)	0.761 (0.733,0.789)	0.762 (0.735,0.790)	0.007 (–0.001,0.004)
Endometrial	0.687 (0.638,0.739)	0.710 (0.662,0.760)	0.023 (–0.008,0.054)	0.697 (0.650,0.746)	0.010 (–0.021,0.041)	–0.013 (–0.036,0.010)	0.6883 (0.64,0.741)	0.676 (0.632,0.723)	–0.012 (–0.042,0.017)
Ovarian	0.621 (0.567,0.680)	0.626 (0.571,0.687)	0.005 (–0.016,0.026)	0.620 (0.566,0.679)	–0.001 (–0.014,0.011)	–0.006 (–0.028,0.016)	0.6216 (0.568,0.681)	0.643 (0.591,0.700)	0.022 (–0.011,0.054)
Post-menopausal breast cancer	0.576 (0.559,0.595)	0.583 (0.565,0.601)	0.006 (–0.006,0.018)	0.584 (0.566,0.602)	0.007 (–0.005,0.019)	0.001 (–0.007,0.009)	0.577 (0.559,0.595)	0.584 (0.566,0.603)	0.007 (–0.006,0.021)

* Multivariable adjustment for baseline age, race, alcohol, smoking and HRT (in women).

*OBR* obesity-related, *NOR* non-obesity related, *BMI* body mass index, *CI* confidence interval, *WC* waist circumference.

In women, for pancreatic, kidney, endometrial, ovarian and post-menopausal breast cancer, there were non-significant differences in the predictive performances across metrics (Table 4). For the combined non-obesity-related cancer subgroup without lung cancers in women, there was a significantly higher C-statistic for single BMI than WC of 0.589 (95% CI:0.571,0.607) and 0.582 (95% CI:0.565,0.601) respectively with a difference of 0.006 (95% CI:0.000,0.013). For colorectal cancer in women, there was a significantly higher C-statistic per SD waist circumference-years than a single WC measure with C-statistics of 0.618 (95% CI:0.588,0.650) and 0.590 (95% CI:0.559,0.621) respectively and a difference of 0.029 (95% CI:0.051,0.006). Furthermore, waist circumference-years had a significantly higher C-statistic than single BMI with a C-statistic of the latter of 0.586 (95% CI:0.556,0.618) and a difference of 0.032 (95% CI:0.061,0.004) (Table 4).

### Sensitivity analysis

Results in the sensitivity analysis using lower WC thresholds were relatively similar to the main analysis thresholds (Table S13–S20). Results in the sensitivity analysis in the subgroup with yearly WC predicted from participants with at least 1 WC measurement (Table S21–S28) varied from the main analysis in that for combined obesity-related cancers, there were non-significant positive associations per SD waist circumference-years exposure in men and women with HRs of 1.14 (95% CI:0.99,1.30) and 1.12 (95% CI:1.00,1.25) respectively (Table S23). For colorectal cancer in men, the positive association per SD waist circumference-years was non-significant per SD increment in waist circumference 1.16 (95% CI:0.96,1.4). In women, there was a significant positive association only for colorectal cancer per SD waist circumference years 1.47 (95% CI:1.14,1.89). Similar associations were found overall in the main analysis on additional analysis of White participants separately (Table S29–S36). There were no differences across C-statistics in the performance characteristics (Table S36a, S36b). On analysis of Black participants separately (Table S37–S44), there were no significant associations per SD waist circumference-years for combined cancer subgroups, but by cancer type the significant association found was only for colorectal cancer 1.37 (95% CI:1.13,1.67) in men and endometrial cancer in women 1.69 (95% CI:1.15, 2.5) (Table S39). For lung cancer, varied directions of associations were found per SD increment in waist circumference-years in men with a non-significant but positive association in Black men of 1.17 (95% CI:0.90,1.53) unlike White men where there was a non-significant inverse association [HR 0.96 (95% CI:0.84,1.10)] (Table S39, Table S31). There were no differences in the performance characteristics between any of the metrics across all cancer sites (Table S44a, S44b).

## Discussion

In this prospective cohort study of White and Black men and women, we found that waist circumference-years metric quantified using waist circumference measurements predicted per year at an older age of assessment (from age 44 years on average onwards), had significant positive associations with all cancers combined, obesity-related cancers combined, and colorectal cancer in men and women. Significant positive associations with waist circumference-years were also found for non-obesity-related cancers when excluding lung and prostate cancers in men and kidney, endometrial and post-menopausal breast cancer in women. Overall, the performance characteristics across all metrics – waist circumference-years, single WC and single BMI – were similar except for lung cancer in men and combined non-obesity-related cancers in women excluding lung where BMI had a significantly higher predictive performance than a single WC measure. We found that for colorectal cancer in women, waist circumference-years had a significantly higher predictive performance than both single BMI and WC measures.

On analysis of the cumulative degree and duration of excess WC, for all cancers and non-obesity-related cancers in men significant positive associations were found for the cumulative degree but not duration of excess WC. Duration of excess WC had a significant positive association with bladder and lung cancer in men. In women, cumulative duration, but not degree of excess WC, had a significant positive association with obesity-related; however, cumulative degree and duration of excess WC had a significant positive association for colorectal cancer in women. On analysis of the predictive performance for cumulative degree and duration of excess WC, marginal differences were found across the metrics in men and women, except for bladder cancer in men where duration of excess WC had a significantly higher predictive performance than the degree of excess WC.

Many studies have analysed excess adiposity throughout adulthood and the related cancer risk [54]; however, these studies have primarily used single BMI or to a lesser extent single WC measurements. The Framingham Study showed that WC had a stronger association with colon cancer incidence than BMI in women [55]. Our study did not find a significant difference in the predictive performance of single WC and BMI measures for colorectal cancer but found a significantly greater predictive performance for waist circumference-years compared to single BMI and WC measures. For pancreatic cancer, there were non-significant positive associations for waist circumference-years, single WC and BMI exposures. This differs from some prior studies which showed no associations between WC and pancreatic cancer [56, 57]; however, our findings are in line with an umbrella review on WC and pancreatic cancer associations with the suggestive association between WC and pancreatic cancer [5]. For kidney cancer, non-significant inverse associations were found in all exposure metrics explored in men; however, in women, significant positive associations were identified across all metrics. Prior studies on European and East Asian cohorts showed positive associations between WC and renal cancer [58, 59]. Bladder cancer also had positive but non-significant associations in men for waist circumference-years, single WC and BMI exposures. In men, duration of excess WC had a significant positive association unlike the degree. Cumulative excess WC duration had a significantly higher discriminatory predictive performance than the degree of excess WC in men. A recent systematic review and meta-analysis of 27 studies supports the positive association between central adiposity and bladder cancer incidence with a risk ratio of 1.18 (95%CI:1.09,1.26) [60].

For lung cancer in men, there was an inverse association with a single BMI but a non-significant positive association for waist circumference-years. A potential explanation is that increased smoking consumption is associated with increased abdominal distributions of adipose tissue for a given BMI. A Mendelian randomisation meta-analysis in 2015 of 148,731 participants of European ancestry from 29 studies showed for a given BMI, there were associations between a genetic variant associated with increased consumption of cigarettes and increased WC [61]. However, in our study, single WC also had an inverse association with cancer risk, potentially showing the importance of accounting for cumulative WC exposure through waist circumference-years. Potential mechanisms underlying cumulative excess WC include elevated cortisol levels [62]. In women, there was an inverse association for lung cancer across all metrics which is not in-line with a previous systematic review and meta-analysis of 6 studies showing significant positive associations between a single WC measure and lung cancer [63]. In the sensitivity analysis of our study with waist circumference-years derived from predicted WC in those with at least 1 observed WC measure, waist circumference-years, single WC and cumulative degree of excess WC had significant positive associations with lung cancer incidence in men potentially indicating a selection bias towards healthier individuals with at least 3 observed WC measures. In alignment with a recent study on four Swedish cohorts, our study found that none of the metrics were associated with prostate cancer or metastatic disease and no differences in the discriminatory predictive performance of metrics [64].

There were significant positive associations across all metrics for endometrial and post-menopausal breast cancer in women and no difference in the predictive performance of metrics highlighting overall and central excess adiposity contribute to endometrial and post-menopausal breast cancer risk. For ovarian cancer in women, there were positive associations across waist circumference-years, single WC and BMI exposures; however, an inverse association was identified for ovarian cancer per cumulative degree of excess WC exposure on age adjustment. Prior analysis of single WC and ovarian cancer on NIH-AARP found a non-significant but positive association [65].

Overall, findings from our sensitivity analysis using lower WC thresholds were like those of the main analysis except in women where numerically higher positive associations were found using lower thresholds. A prospective study by Reis et al. highlighted similar associations and AIC measures for the lower WC thresholds compared with standard WC thresholds used [32]. So lower WC thresholds are of clinical relevance given the higher association with cancer incidence.

In US adults of all demographic groups, socioeconomic and education levels, the prevalence of obesity has predominantly increased since the 1970s [66]. Visit 1 in ARIC started in 1987–1989, so this study has captured the epidemic in middle-aged and older adults and somewhat representatives the last 2.5 decades of exposure. However, with increasing exposure per year of adiposity worldwide, findings are potentially underestimated when relating to the current population and to participants that lived with excess adiposity in earlier life or whose mothers were living with excess adiposity entering pregnancy. Prior studies that used waist circumference-years analysed associations with the incidence of Type 2 diabetes, cardiovascular disease and stroke [32, 67, 68]. All three studies identified had little additional risk using the time-dependent waist circumference-years and either single BMI or WC measures; there were minimal variations in the AIC, a goodness of fit measure, across metrics [32, 67, 68]. No study has analysed waist circumference-years exposure and cancer incidence [5]. Comparable associations were previously found between single WC and BMI measures and cancer incidence [6]. Single BMI and WC measures may be favoured over waist circumference-years for the simplicity of data collection in the clinic; however, the duration of excess WC exposure was potentially limited given it was only measured from age 44 thus underestimates associations with cancer incidence.

### Strengths

A strength of this study was the novel application of waist circumference-years in cancer epidemiology on a cohort with detailed covariate information with a reasonably long follow-up for cancer confirmed by registries. Analysis by race, smoking and HRT between waist circumference-years and cancer sites added further novelty. An additional strength was the number of participants included with at least 3 repeated WC measures. Also, WC was measured by trained technicians following a protocol reducing the likelihood of measurement errors or biases [69].

### Limitations

A limitation of this study was that only one cohort was analysed, so the generalisability of findings needs to be explored in other populations such as Asians, Hispanics and other racial or ethnic groups. A further study limitation was that participants did not have the option to self-report if they were bi-racial or mixed-race; this is an important aspect to consider in future studies. Multiple imputation was used to handle the missing covariate data; however, this method relies on the assumption that covariate information was missing at random, which is a limitation of this study. A further limitation included the lack of menopause data as menopause data was only collected at Visit 1 of the ARIC study and could not be assumed to have remained constant till the index date, Visit 4 of the ARIC study.

Also, WC was only predicted from age 44 given this was the minimum age at which WC was recorded; therefore, cumulative excess WC duration measures were limited to a minimum exposure period of 9 years (Visit 1 to Visit 4). Duration of excess WC exposure is likely to be underestimated as earlier adulthood exposure prior to age 44 and related associations with cancer were not captured. A 9-year time span between Visit 1 to Visit 4 in middle to older adulthood may not allow enough time for variation in the degree of WC to show a different association than that at Visit 4. Analysis of interactions by race for Black participants was limited given the low sample size/event rate for some cancer sites such as bladder cancer, so associations in these groups could not be performed.

### Unanswered questions and future research

Future work should focus on analysing excess WC exposure in early adulthood given this study only analysed WC from age 44 onwards and mid to late adulthood WC is primarily analysed in the literature. A time-dependent continuous waist circumference-years measure using updated WC or cumulative average updated WC could be used in the future to analyse life course waist circumference-years and the related cancer risk. Future work should analyse the association between waist circumference-years and cancer incidence and other cohorts that have at least 3 WC measures to identify whether findings in this study are generalisable to other populations. Other metrics used to quantify excess adiposity exposure including waist-hip ratio, body-fatness percentage and magnetic resonance imaging could be used similarly to waist circumference-years to further analyse cumulative exposures by degree and duration of excess adiposity with cancer. Future studies should also consider stratifying by more ancestral groups in cohorts where such data are available to analyse associations between waist circumference-years or a single WC measure and cancer incidence by ancestry. Future studies could explore ancestry-specific WC thresholds and apply them to waist circumference-years measures to explore their association with cancer incidence.

## Conclusion

We identified significant positive associations between waist circumference-years and cancer incidence, for colorectal cancer in men, and colorectal, kidney, endometrial and postmenopausal breast cancer in women. Waist-circumference years did not give more information about cancer risk than WC and BMI, except for colorectal cancer in women where waist circumference-years had a significantly higher predictive performance than single BMI and WC measures. Single WC may be sufficient, but BMI remains the metric of preference given its routine measurement in clinical practice.

## Supplementary information

Supplementary material

## Data Availability

ARIC data can be requested via: https://sites.cscc.unc.edu/aric/distribution-agreements. Further information is available from the corresponding author upon request.

## Code availability

Code will be provided by the corresponding author upon reasonable request and approval by the ethics committee.
