# Supplementary material for: Waist circumference-years and cancer risk: a prospective study of the association and comparison of predictive performance with waist circumference and body mass index
Source: Br J Cancer. 2024 Oct 4;131(10):1623–34. doi: 10.1038/s41416-024-02860-y (PMC11554801; doi:10.1038/s41416-024-02860-y)
Supplement: Supplementary file 1 — Supplementary material [file 41416_2024_2860_MOESM1_ESM.docx]

**Supplementary**

1. **Figure S1: Diagram of the exposure and follow-up period of this study.** *The period of exposure is defined as the period from ARIC study entry at Visit 1 till the index date defined at Visit 4 in this study. In the exposure period, waist circumference-years exposure was calculated for any WC measurements greater than or equal to 102 cm in men and 88 cm in women. The period of cancer follow-up was from the index date (Visit 4) till the end of cancer follow-up (cancer outcome/administrative censoring 31^st^ December 2015).*
2. **Table S1: Waist circumference-years calculation hypothetical example of a male participant.** Example calculation of waist circumference-years at age 57 = prior degree of WC (0.1) x duration at that WC exposure = (0.1 x 1) = 0.1 waist circumference-years. The interval is the period between the prior and current reading. The degree of excess WC (cm) is the WC minus 101.9. WC measurements < 102cm would have a degree of excess WC of 0. The degree of WC is subtracted by 101.9 to include WC exposures at 102 cm. The duration of excess WC is the period in a degree of excess WC exposure ≥ 102 cm. Cumulative excess waist circumference degree is the cumulative sum of degree of excess waist circumference exposure and cumulative excess waist circumference duration is the cumulative sum of the duration of excess waist circumference exposure. **Abbreviation**: WC, waist circumference
3. **Table S2: Strengthening the Reporting of Observational studies in Epidemiology (STROBE) guidelines checklist.**
4. **Table S3: Summary of the exposure metrics in the ARIC cohort.**
5. **Table S4: Incidence of cancer (events/1000 Person-Years) according to cumulative waist circumference-years stratified by biological sex, race, smoking, HRT in the ARIC cohort.**
6. **Table S5: Hazard ratio of specific cancers related to waist circumference-years in the ARIC cohort.**
7. **Table S6: Comparison of the waist circumference degree and duration per 10 units and per 10 years respectively in the ARIC cohort.**
8. **Table S7: Comparison of the metrics by Akaike information criterion, ARIC cohort.**
9. **Table S8: Comparison of combined metrics using Harrell’s C-statistic, ARIC.**
10. **Table S9: Hazard ratio of specific cancers related to waist circumference years, single WC and single BMI by race in (a) men and (b) women.**
11. **Table S10: Hazard ratio of specific cancers related to waist circumference years, single WC and single BMI by smoking in (a) men and (b) women.**
12. **Table S11: Hazard ratio of cancers per standard deviation of waist circumference-years at Visit 4 and BMI at Visit 4, ARIC with additional adjustment for smoking pack-years.**
13. **Table S12: Hazard ratio of specific cancers related to waist circumference-years and baseline BMI by HRT status.**
14. **Table S13: Summary of the exposure metrics using lower waist circumference thresholds.**
15. **Table S14: Incidence of cancer (events/1000 Person-Years) according to cumulative waist circumference years stratified by biological sex, race, smoking, HRT in the ARIC cohort using lower waist circumference thresholds.**
16. **Table S15: Hazard ratio of specific cancers related to waist-circumference years in the ARIC cohort using lower waist circumference thresholds.**
17. **Table S16: Hazard ratio of specific cancers related to waist circumference years in the ARIC cohort using lower waist circumference thresholds.**
18. **Table S17: Comparison of the waist circumference degree and duration per unit standard deviation using lower waist circumference thresholds.**
19. **Table S18: Comparison of the waist circumference degree and duration per 10 units and per 10 years respectively using predicted BMI from cohort with at least 3 BMI measurements in the ARIC cohort using lower waist circumference thresholds.**
20. **Table S19: Comparison of the metrics calculated using WC predicted from the subgroup with at least 3 WC measurements by Akaike information criterion in the ARIC cohort using lower waist circumference thresholds.**
21. **Table S20a and S20b: Comparison of the waist circumference-years metric, single WC and single BMI each at Visit 4 using Harrell’s C-statistic, ARIC, using lower waist circumference thresholds.**
22. **Table S21: Summary of the exposure metrics from participants with at least 1 observed WC measurement.**
23. **Table S22: Incidence of cancer (events/1000 Person-Years) according to cumulative waist circumference years stratified by biological sex, race, smoking, HRT from participants with at least 1 observed WC measurement.**
24. **Table S23: Hazard ratio of specific cancers related to waist-circumference years derived using WC predicted from participants with at least 1 observed WC measurement.**
25. **Table S24: Hazard ratio of specific cancers related to waist circumference years derived using WC predicted from participants with at least 1 observed WC measurement.**
26. **Table S25: Comparison of the waist circumference degree and duration per unit standard deviation derived using WC predicted from participants with at least 1 observed WC measurement.**
27. **Table S26: Comparison of the waist circumference degree and duration per 10 units and per 10 years respectively derived using WC predicted from participants with at least 1 observed WC measurement.**
28. **Table S27: Comparison of the metrics calculated using WC predicted from the subgroup with at least 3 WC measurements by Akaike information criterion derived using WC predicted from participants with at least 1 observed WC measurement.**
29. **Table S28a S28b: Comparison of the waist circumference-years metric, single WC and single BMI each at Visit 4 using Harrell’s C-statistic**  **derived using WC predicted from participants with at least 1 observed WC measurement.**
30. **Table S29: Summary of the exposure metrics in White participants only.**
31. **Table S30: Incidence of cancer (events/1000 Person-Years) according to cumulative waist circumference years stratified by biological sex, smoking, HRT in White participants only.**
32. **Table S31: Hazard ratio of specific cancers related to waist-circumference years in White participants only.**
33. **Table S32: Hazard ratio of specific cancers related to waist circumference years in White participants only.**
34. **Table S33: Comparison of the waist circumference degree and duration per unit standard deviation in White participants only.**
35. **Table S34: Comparison of the waist circumference degree and duration per 10 units and per 10 years respectively stratified by biological sex in White participants only.**
36. **Table S35: Comparison of the metrics calculated using WC predicted from the subgroup with at least 3 WC measurements by Akaike information criterion in White participants only.**
37. **Table S36a and S36b: Comparison of the metrics calculated using WC predicted from the subgroup**  **in White participants only by Harrell’s C-index.**
38. **Table S37: Summary of the exposure metrics in Black participants only.**
39. **Table S38: Incidence of cancer (events/1000 Person-Years) according to cumulative waist circumference years stratified by biological sex, race, smoking, HRT in Black participants only.**
40. **Table S39: Hazard ratio of specific cancers related to waist-circumference years in Black participants only.**
41. **Table S40: Hazard ratio of specific cancers related to waist circumference years in Black participants only.**
42. **Table S41: Comparison of the waist circumference degree and duration per unit standard deviation in Black participants only.**
43. **Table S42: Comparison of the waist circumference degree and duration per 10 units and per 10 years respectively in Black participants only.**
44. **Table S43: Comparison of the metrics by Akaike information criterion in Black participants only.**
45. **Table S44a and S44b: Comparison of the waist circumference-years metric, single WC and single BMI each at Visit 4 using Harrell’s C-statistic in Black participants only.**

**Figure S1: Diagram of the exposure and follow-up period of this study.**

Time (years)

Start of follow-up in 1996-1998

(Index date)

(Visit 4)

Exposure period

Cancer follow-up period

End of cancer follow-up

31^st^ December 31^st^ 2015

(Cancer outcome/administrative censoring)

Study entry in 1987-1989

(Visit 1)

Immortal time

Waist circumference-years in women

**WC ≥ 88 cm in women**

**WC ≥ 102 cm in men**

Waist circumference-years in men

*The period of exposure is defined as the period from ARIC study entry at Visit 1 till the index date defined at Visit 4 in this study. In the exposure period, waist circumference-years exposure was calculated for any WC measurements greater than or equal to 102 cm in men and 88 cm in women. The period of cancer follow-up was from the index date (Visit 4) till the end of cancer follow-up (cancer outcome/administrative censoring 31^st^ December 2015).*

**Table S1: Waist circumference-years calculation hypothetical example of a male participant.**

| **Age (years)** | **Interval**  **(years)** | **WC (cm)** | **Degree of excess WC (cm)** | **Duration of excess WC (years)** | **Waist circumference-years**  **(cm-years)** | **Cumulative waist circumference-years**  **(cm-years)** | **Cumulative excess WC degree**  **(cm)** | **Cumulative excess WC duration**  **(years)** |
| --- | --- | --- | --- | --- | --- | --- | --- | --- |
| 55 | NA | 99.0 | 0.0 | NA | 0.0 | 0.0 | 0.0 | 0 |
| 56 | 1 | 102.0 | 0.1 | 0 | 0.0 | 0.0 | 0.1 | 0 |
| 57 | 1 | 104.0 | 2.1 | 1 | 0.1 | 0.1 | 2.2 | 1 |
| 58 | 1 | 103.0 | 1.1 | 1 | 2.1 | 2.2 | 3.3 | 2 |

Example calculation of waist circumference-years at age 57 = prior degree of WC (0.1) x duration at that WC exposure = (0.1 x 1) = 0.1 waist circumference-years. The interval is the period between the prior and current reading. The degree of excess WC (cm) is the WC minus 101.9. WC measurements < 102cm would have a degree of excess WC of 0. The degree of WC is subtracted by 101.9 to include WC exposures at 102 cm. The duration of excess WC is the period in a degree of excess WC exposure ≥ 102 cm. Cumulative excess waist circumference degree is the cumulative sum of degree of excess waist circumference exposure and cumulative excess waist circumference duration is the cumulative sum of the duration of excess waist circumference exposure.

**Abbreviation**: WC, waist circumference

**Table S2: Strengthening the Reporting of Observational studies in Epidemiology (STROBE) guidelines checklist.**

|  | Item No. | Recommendation | Page  No. | Relevant text from manuscript |
| --- | --- | --- | --- | --- |
| **Title and abstract** | 1 | (*a*) Indicate the study’s design with a commonly used term in the title or the abstract | 2 | “Prospective Atherosclerosis Risk in Communities Study” |
|  |  | (*b*) Provide in the abstract an informative and balanced summary of what was done and what was found | 2 | “Associations between cross-sectional and longitudinal BMI with cancer incidence have been explored, but the association and predictive performance of a cumulative central adiposity measure, waist circumference-years is lacking.” |
| Introduction | | | |  |
| Background/rationale | 2 | Explain the scientific background and rationale for the investigation being reported | 3 | “There is sufficient strength of evidence of an association between at least thirteen cancer types with body mass index (BMI); however, these findings are mainly based on a single BMI measure (1).”  “What has yet to be explored in the cancer literature which has formulated the aims of this paper, is whether a metric that combines the degree of excess WC with the duration of excess WC exposure, known as waist circumference-years, has an association with cancer incidence.” |
| Objectives | 3 | State specific objectives, including any prespecified hypotheses | 3 | “The aims of this study were to 1) explore the association between waist circumference years and cancer incidence, 2) explore the association between cumulative degree or duration of excess WC over adulthood with cancer incidence, 3) compare the predictive performance of waist circumference-years with single WC and BMI measures and 4) compare the predictive performance of measures of cumulative degree and cumulative duration of excess WC with cancer incidence.” |
| Methods | | | |  |
| Study design | 4 | Present key elements of study design early in the paper | 3,4 | “Atherosclerosis Risk in Communities (ARIC) study is a prospective cohort study” |
| Setting | 5 | Describe the setting, locations, and relevant dates, including periods of recruitment, exposure, follow-up, and data collection | 3,4,5,6 | Under methods section |
| Participants | 6 | (*a*) *Cohort study*—Give the eligibility criteria, and the sources and methods of selection of participants. Describe methods of follow-up  *Case-control study*—Give the eligibility criteria, and the sources and methods of case ascertainment and control selection. Give the rationale for the choice of cases and controls  *Cross-sectional study*—Give the eligibility criteria, and the sources and methods of selection of participants | 3,4 |  |
|  |  | (*b*) *Cohort study*—For matched studies, give matching criteria and number of exposed and unexposed  *Case-control study*—For matched studies, give matching criteria and the number of controls per case | NA |  |
| Variables | 7 | Clearly define all outcomes, exposures, predictors, potential confounders, and effect modifiers. Give diagnostic criteria, if applicable | 3,4 |  |
| Data sources/ measurement | 8* | For each variable of interest, give sources of data and details of methods of assessment (measurement). Describe comparability of assessment methods if there is more than one group | *4* |  |
| Bias | 9 | Describe any efforts to address potential sources of bias | 6 | “the main analysis was repeated including participants with at least 1 WC reading to identify any healthy volunteer bias in the main analysis through the selection of those with at least 3 WC measures.” |
| Study size | 10 | Explain how the study size was arrived at | 4 | “In the main analysis, we analysed cancer sites that fulfilled the criteria of having at least 10 events per candidate predictor parameter (EPP) separately” |

Continued on next page

| Quantitative variables | 11 | Explain how quantitative variables were handled in the analyses. If applicable, describe which groupings were chosen and why |  | " |
| --- | --- | --- | --- | --- |
| Statistical methods | 12 | (*a*) Describe all statistical methods, including those used to control for confounding | 5,6 | Statistical analysis section |
|  |  | (*b*) Describe any methods used to examine subgroups and interactions | 6 | Sensitivity analysis section |
|  |  | (*c*) Explain how missing data were addressed | 5 | “missing covariate data were assumed to be missing at random and were handled by multiple imputation to create ten imputed datasets” |
|  |  | (*d*) *Cohort study*—If applicable, explain how loss to follow-up was addressed  *Case-control study*—If applicable, explain how matching of cases and controls was addressed  *Cross-sectional study*—If applicable, describe analytical methods taking account of sampling strategy | 5 | “were followed through and censored on the incidence of cancer, death, or administrative censoring (31/12/2015).” |
|  |  | (*e*) Describe any sensitivity analyses |  |  |
| Results | | | | |
| Participants | 13* | (a) Report numbers of individuals at each stage of study—eg numbers potentially eligible, examined for eligibility, confirmed eligible, included in the study, completing follow-up, and analysed | 7 | Figure 1 |
|  |  | (b) Give reasons for non-participation at each stage | 7 | Figure 1 |
|  |  | (c) Consider use of a flow diagram | 7 | Figure 1 |
| Descriptive data | 14* | (a) Give characteristics of study participants (eg demographic, clinical, social) and information on exposures and potential confounders | 7 |  |
|  |  | (b) Indicate number of participants with missing data for each variable of interest | 7 | Table 1 |
|  |  | (c) *Cohort study*—Summarise follow-up time (eg, average and total amount) | 7 | “followed up for 13.7 (standard deviation [SD] 6.1) and 15.8 (SD 5.2) years respectively” |
| Outcome data | 15* | *Cohort study*—Report numbers of outcome events or summary measures over time |  | *Number of events displayed in Table 2* |
|  |  | *Case-control study—*Report numbers in each exposure category, or summary measures of exposure |  | *NA* |
|  |  | *Cross-sectional study—*Report numbers of outcome events or summary measures |  | *NA* |
| Main results | 16 | (*a*) Give unadjusted estimates and, if applicable, confounder-adjusted estimates and their precision (eg, 95% confidence interval). Make clear which confounders were adjusted for and why they were included | 7,8,9 | Under heading results |
|  |  | (*b*) Report category boundaries when continuous variables were categorized |  | Per standard deviation exposure was used. |
|  |  | (*c*) If relevant, consider translating estimates of relative risk into absolute risk for a meaningful time period | 7 | Incidence rates compared. |

| Other analyses | 17 | Report other analyses done—eg analyses of subgroups and interactions, and sensitivity analyses | 6 | Under heading sensitivity analysis |
| --- | --- | --- | --- | --- |
| Discussion | | | | |
| Key results | 18 | Summarise key results with reference to study objectives | 10 |  |
| Limitations | 19 | Discuss limitations of the study, taking into account sources of potential bias or imprecision. Discuss both direction and magnitude of any potential bias | 11 |  |
| Interpretation | 20 | Give a cautious overall interpretation of results considering objectives, limitations, multiplicity of analyses, results from similar studies, and other relevant evidence | 10,11,12 |  |
| Generalisability | 21 | Discuss the generalisability (external validity) of the study results | 12 | “A limitation of this study is that we were only able to analyse WC in one cohort, and therefore the generalisability of the findings need to be explored in other populations” |
| Other information | |  | | |
| Funding | 22 | Give the source of funding and the role of the funders for the present study and, if applicable, for the original study on which the present article is based | 13 | Under heading funding |

*Give information separately for cases and controls in case-control studies and, if applicable, for exposed and unexposed groups in cohort and cross-sectional studies.

**Note:** An Explanation and Elaboration article discusses each checklist item and gives methodological background and published examples of transparent reporting. The STROBE checklist is best used in conjunction with this article (freely available on the Web sites of PLoS Medicine at http://www.plosmedicine.org/, Annals of Internal Medicine at http://www.annals.org/, and Epidemiology at http://www.epidem.com/). Information on the STROBE Initiative is available at www.strobe-statement.org.

| **Characteristic** | **Men** |
| --- | --- |
| **Baseline BMI, kg/m^2^** | 28.50 (4.50) |
| **Baseline WC, cm** | 103.00 (12.00) |
| **Follow up, years** | 13.70 (6.10) |
| **Total cumulative waist circumference years, cm-years** | 32.00 (58.00) |
| **Total cumulative waist circumference degree, cm** | 16.00 (27.00) |
| **Total cumulative waist circumference duration, years** | 4.24 (4.04) |
|  | **Women** |
| **Baseline BMI, kg/m^2^** | 29.00 (6.00) |
| **Baseline WC, cm** | 101.00 (16.00) |
| **Follow up years, years** | 15.80 (5.20) |
| **Total cumulative waist circumference years, cm-years** | 101.00 (113.00) |
| **Total cumulative excess waist circumference degree, cm** | 48.00 (51.00) |
| **Total cumulative excess waist circumference duration, years** | 6.63 (3.41) |
| **Mean (SD)**  **Abbreviations:** N = number of participants; SD, standard deviation; BMI, body mass index, WC, waist circumference. | |

**Table S3: Summary of the exposure metrics in the ARIC cohort.**

**Table S4: Incidence of cancer (events/1000 Person-Years) according to cumulative waist circumference-years stratified by biological sex, race, smoking, HRT in the ARIC cohort.**

| **Men** | | | | | | | | | | | | |
| --- | --- | --- | --- | --- | --- | --- | --- | --- | --- | --- | --- | --- |
|  | **0 cm-years** | | | **≤100 cm-years** | | | **>100 cm-years** | | | **Baseline cohort** | | |
|  | **N** | **PYFU** | **IR (95% CI)** | **N** | **PYFU** | **IR (95% CI)** | **N** | **PYFU** | **IR (95% CI)** | **N** | **PYFU** | **IR (95% CI)** |
| **Whole sample** | 760 | 144018.80 | 5.28 (4.90,5.66) | 487 | 91142.26 | 5.34 (4.86,5.82) | 267 | 41240.44 | 6.47 (5.69,7.26) | 1514 | 276401.50 | 5.48 (5.20,5.76) |
| **Race** | | | | | | | | | | | | |
| **White** | 575 | 115509.90 | 4.98 (4.57,5.39) | 414 | 78456.35 | 5.28 (4.76,5.79) | 224 | 35864.09 | 6.25 (5.41,7.08) | 1213 | 229830.30 | 5.28 (4.98,5.58) |
| **Black** | 185 | 28508.96 | 6.49 (5.54,7.44) | 73 | 12685.91 | 5.75 (4.39,7.12) | 43 | 5376.35 | 8.00 (5.51,10.49) | 301 | 46571.23 | 6.46 (5.72,7.20) |
| **Smoking** | | | | | | | | | | | | |
| **Ever** | 555 | 98286.49 | 5.65 (5.17,6.12) | 374 | 66675.37 | 5.61 (5.03,6.19) | 195 | 30308.06 | 6.42   (5.50,  7.34) | 1124 | 195269.90 | 5.76 (5.42,6.09) |
| **Never** | 205 | 45732.34 | 4.48 (3.85,5.10) | 113 | 24466.90 | 4.61 (3.74,5.49) | 72 | 10932.38 | 6.62 (5.05,8.20) | 390 | 81131.62 | 4.81 (4.32,5.29) |
| **Women** | | | | | | | | | | | | |
| **Whole sample** | 233 | 94126.21 | 2.48 (2.15,2.80) | 378 | 134512.00 | 2.81 (2.52,3.10) | 628 | 180635.10 | 3.48 (3.20,3.75) | 1239 | 409273.30 | 3.03 (2.86,3.20) |
| **Race** | | | | | | | | | | | | |
| **White** | 198 | 80955.68 | 2.45 (2.10,2.79) | 298 | 103507.50 | 2.88 (2.55,3.21) | 433 | 116770.60 | 3.71 (3.35,4.06) | 929 | 301233.70 | 3.08 (2.88,3.28) |
| **Black** | 35 | 13170.53 | 2.66 (1.74,3.58) | 80 | 31004.52 | 2.58 (2.00,3.16) | 195 | 63864.56 | 3.05 (2.62,3.49) | 310 | 108039.60 | 2.87 (2.55,3.19) |
| **Smoking** | | | | | | | | | | | | |
| **Ever** | 143 | 46932.14 | 3.04 (2.53,3.55) | 211 | 63431.27 | 3.33 (2.87,3.78) | 309 | 77592.32 | 3.99 (3.54,4.44) | 663 | 187955.70 | 3.53 (3.26,3.80) |
| **Never** | 90 | 47194.08 | 1.92 (1.51,2.32) | 167 | 71080.70 | 2.35 (1.99,2.71) | 319 | 103042.80 | 3.09 (2.75,3.44) | 576 | 221317.60 | 2.60 (2.39,2.82) |
| **HRT** | | | | | | | | | | | | |
| **Ever** | 140 | 57134.06 | 2.44 (2.03,2.86) | 200 | 73550.28 | 2.72 (2.34,3.10) | 261 | 79655.35 | 3.28 (2.87,3.68) | 601 | 210339.70 | 2.86 (2.63,3.09) |
| **Never** | 94 | 36992.15 | 2.53 (2.00,3.06) | 178 | 60961.70 | 2.92 (2.48,3.36) | 367 | 100979.80 | 3.63 (3.26,4.01) | 638 | 198933.60 | 3.21 (2.96,3.46) |
| **Abbreviations: N, number of cancer events; PYFR, person-years of follow-up; IR, incidence rate of all cancers; CI, confidence interval, WC, waist circumference.** | | | | | | | | | | | | |

**Table S5: Hazard ratio of specific cancers related to waist circumference-years in the ARIC cohort.**

| **Outcomes** | **Cancers** | **Waist circumference-years (per 100 cm-years)** | | **Baseline WC (per 5 cm)** | | **Baseline BMI (per 5 kg/m2)** | |
| --- | --- | --- | --- | --- | --- | --- | --- |
|  |  | **Age-adjusted HR (95% CI)** | **MV-adjusted HR**  **(95% CI)** | **Age-adjusted HR**  **(95% CI)** | **MV-adjusted HR**  **(95% CI)** | **Age-adjusted HR**  **(95% CI)** | **MV-adjusted HR**  **(95% CI)** |
|  |  | **Men** | | | | | |
| All Cancers | 1,514 | 1.08 (1.03,1.14) | 1.09 (1.03,1.14) | 1.02 (0.99,1.04) | 1.02  (1.00,1.04) | 1.03 (0.97,1.09) | 1.03 (0.97,1.09) |
| OBR-cancers | 306 | 1.16 (1.05,1.28) | 1.16 (1.05,1.28) | 1.06 (1.01,1.11) | 1.06 (1.01,1.11) | 1.15  (1.02,1.30) | 1.15  (1.02,1.30) |
| NOBR-cancers | 1,208 | 1.06 (1.01,1.13) | 1.07 (1.01,1.13) | 1.01 (0.98,1.03) | 1.01 (0.98,1.03) | 1.00  (0.94,1.07) | 1.00  (0.94,1.07) |
| NOBR-cancers excluding lung and prostate | 426 | 1.17 (1.08,1.27) | 1.17 (1.07,1.27) | 1.06  (1.02,1.10) | 1.05  (1.01,1.10) | 1.13 (1.01,1.25) | 1.13 (1.01,1.25) |
| **Specific cancer sites** | | | | | | | |
| Colorectal | 126 | 1.25  (1.10,1.43) | 1.25 (1.10,1.43) | 1.10  (1.03,1.19) | 1.11 (1.03,1.19) | 1.31 (1.09,1.57) | 1.3  (1.09,1.57) |
| Pancreas | 45 | 1.05 (0.76,1.44) | 1.06 (0.77,1.46) | 1.05 (0.92,1.19) | 1.06  (0.93,1.20) | 1.19 (0.86,1.64) | 1.18 (0.86,1.63) |
| Kidney | 55 | 0.96 (0.69,1.32) | 0.97 (0.70,1.33) | 0.98 (0.88,1.11) | 0.99 (0.88,1.11) | 0.96 (0.71,1.31) | 0.96 (0.71,1.32) |
| Bladder | 53 | 1.15 (0.89,1.48) | 1.14 (0.87,1.48) | 1.09 (0.97,1.22) | 1.07  (0.95,1.20) | 1.20  (0.89,1.61) | 1.18 (0.88,1.59) |
| Lung | 211 | 1.04 (0.91,1.19) | 1.03 (0.89,1.18) | 0.95  (0.90,1.01) | 0.94  (0.88,1.00) | 0.74 (0.62,0.88) | 0.74 (0.62,0.88) |
| Prostate | 571 | 0.99  (0.90,1.08) | 1.00  (0.91,1.09) | 0.99 (0.95,1.03) | 1.00  (0.97,1.04) | 1.01 (0.92,1.11) | 1.01 (0.92,1.11) |
| Metastatic Prostate | 34 | 0.85 (0.53,1.35) | 0.87 (0.55,1.37) | 0.97 (0.83,1.12) | 0.99 (0.85,1.14) | 0.95  (0.64,1.40) | 0.95  (0.65,1.40) |

| **Women** | | | | | | | |
| --- | --- | --- | --- | --- | --- | --- | --- |
| All Cancers | 1,239 | 1.05 (1.02,1.08) | 1.06  (1.03,1.09) | 1.04 (1.02,1.06) | 1.04 (1.03,1.06) | 1.09  (1.05,1.14) | 1.11  (1.07,1.17) |
| OBR-cancers | 751 | 1.09 (1.05,1.12) | 1.09  (1.06,1.13) | 1.06 (1.04,1.08) | 1.06 (1.04,1.09) | 1.14  (1.08,1.21) | 1.16  (1.09,1.22) |
| NOBR-cancers | 488 | 0.99 (0.95,1.04) | 1.01  (0.96,1.05) | 1.01 (0.98,1.03) | 1.01 (0.99,1.04) | 1.01  (0.94,1.08) | 1.05  (0.97,1.13) |
| NOBR-cancers excluding lung | 336 | 1.00  (0.95,1.05) | 1.01  (0.96,1.07) | 1.01 (0.98,1.05) | 1.02 (0.99,1.06) | 1.05  (0.96,1.14) | 1.09  (0.99,1.18) |
| **Specific cancer sites** | | | | |  |  |  |
| Colorectal | 132 | 1.09 (1.01,1.17) | 1.08  (1.00,1.17) | 1.02 (0.97,1.08) | 1.02 (0.97,1.07) | 1.05  (0.91,1.20) | 1.02  (0.88,1.17) |
| Pancreas | 43 | 1.13  (1.00,1.27) | 1.08  (0.95,1.22) | 1.09  (1.00,1.19) | 1.06 (0.97,1.16) | 1.15  (0.92,1.44) | 1.03  (0.81,1.31) |
| Kidney | 41 | 1.15 (1.02,1.30) | 1.15  (1.01,1.30) | 1.13 (1.03,1.23) | 1.12 (1.03,1.23) | 1.33  (1.08,1.63) | 1.32  (1.06,1.65) |
| Lung | 152 | 0.97 (0.90,1.06) | 0.99  (0.91,1.08) | 0.99 (0.94,1.04) | 1.00  (0.95,1.05) | 0.92  (0.80,1.05) | 0.97  (0.84,1.11) |
| Endometrial | 76 | 1.26 (1.17,1.37) | 1.27  (1.17,1.38) | 1.2 (1.13,1.27) | 1.21 (1.14,1.29) | 1.51  (1.31,1.73) | 1.58  (1.36,1.83) |
| Ovarian | 40 | 0.97 (0.82,1.15) | 1.02  (0.86,1.21) | 1.00  (0.91,1.10) | 1.04 (0.94,1.14) | 0.92  (0.71,1.20) | 1.04  (0.79,1.36) |
| Post-menopausal breast | 357 | 1.04 (0.99,1.10) | 1.05  (1.00,1.11) | 1.04 (1.01,1.08) | 1.05 (1.02,1.09) | 1.11  (1.02,1.20) | 1.14  (1.05,1.23) |
| * Multivariable adjustment for baseline age, race, alcohol, smoking and hormone replacement therapy (in women).  **Abbreviations**: OBR, obesity-related; NOBR, non-obesity related; CI, confidence interval; HR, hazard ratio; BMI, body mass index; MV, multivariable, WC, waist circumference. | | | | | | | |

**Table S6: Comparison of the waist circumference degree and duration per 10 units and per 10 years respectively in the ARIC cohort.**

| **Outcome** | **Cumulative degree of excess WC**  **(per 10 cm)** | | | | **Cumulative duration of excess WC**  **(per 10 years)** | | | |
| --- | --- | --- | --- | --- | --- | --- | --- | --- |
|  | **Age-adjusted HR**  **(95% CI)** | | **MV-adjusted HR**  **(95% CI)** | | **Age-adjusted HR**  **(95% CI)** | **MV-adjusted HR**  **(95% CI)** | | |
| **Men** | | | | | | | | |
| **All Cancers** | 1.01 (1.00,1.02) | | 1.01 (1.00,1.02) | | 1.07 (0.97,1.18) | 1.06 (0.96,1.17) | | |
| **OBR-cancers** | 1.01 (1.00,1.03) | | 1.01 (1.00,1.03) | | 1.06 (0.85,1.32) | 1.06 (0.85,1.32) | | |
| **NOBR-cancers** | 1.01 (1.00,1.02) | | 1.01 (1.00,1.02) | | 1.07 (0.96,1.20) | 1.06 (0.95,1.19) | | |
| **NOBR-cancers excluding lung and prostate** | 1.01 (1.00,1.03) | | 1.02 (1.00,1.03) | | 1.20 (1.00,1.44) | 1.20 (1.00,1.44) | | |
| **Specific cancer sites** | | | | | | | | |
| Colorectal | 1.02 (1.00,1.04) | | 1.02 (0.99,1.04) | | 1.04 (0.75,1.45) | 1.04 (0.75,1.44) | | |
| Pancreas | 0.99 (0.94,1.04) | | 0.99 (0.94,1.04) | | 1.30 (0.74,2.3) | 1.27 (0.72,2.25) | | |
| Kidney | 1.00 (0.95,1.04) | | 1.00 (0.95,1.04) | | 0.85 (0.47,1.53) | 0.87 (0.48,1.57) | | |
| Bladder | 1.00 (0.96,1.04) | | 1.00 (0.96,1.04) | | 2.32 (1.43,3.78) | 2.28 (1.4,3.72) | | |
| Lung | 1.02 (1.01,1.04) | | 1.03 (1.01,1.05) | | 1.31 (1.02,1.68) | 1.25 (0.97,1.60) | | |
| Prostate | 1.00 (0.99,1.01) | | 1.00 (0.98,1.01) | | 0.90 (0.76,1.07) | 0.89 (0.75,1.06) | | |
| Metastatic Prostate | 0.98 (0.92,1.05) | | 0.98 (0.91,1.04) | | 1.12 (0.56,2.24) | 1.09 (0.54,2.18) | | |
| **Women** | | | | | | | | |
| **All Cancers** | 1.00 (1.00, 1.00) | 1.00 (1.00,1.01) | | 1.07 (0.96,1.19) | | | 1.10 (0.99,1.22) |  |
| **OBR-cancers** | 1.00 (1.00,1.01) | 1.00 (1.00,1.01) | | 1.16 (1.01,1.33) | | | 1.17 (1.02,1.35) |  |
| **NOBR-cancers** | 0.99 (0.99,1.00) | 1.00 (0.99, 1.00) | | 0.98 (0.83,1.15) | | | 1.03 (0.87,1.21) |  |
| **NOBR-cancers excluding lung** | 0.99 (0.98, 1.00) | 0.99 (0.99, 1.00) | | 0.96 (0.78,1.17) | | | 0.99 (0.81,1.21) |  |
| **Specific cancer sites** | | | | | | | |  |
| Colorectal | 1.02 (1.01,1.03) | 1.02 (1.00,1.03) | | 1.75 (1.25,2.45) | | | 1.73 (1.23,2.42) |  |
| Pancreas | 1.00 (0.98,1.03) | 1.00 (0.98,1.03) | | 1.33 (0.75,2.37) | | | 1.26 (0.71,2.23) |  |
| Kidney | 1.00 (0.97,1.02) | 1.00 (0.97,1.02) | | 1.60 (0.83,3.08) | | | 1.62 (0.84,3.11) |  |
| Lung | 1.00 (0.98,1.01) | 1.00 (0.98,1.01) | | 1.02 (0.76,1.37) | | | 1.10 (0.82,1.48) |  |
| Endometrial | 1.01 (0.99,1.02) | 1.01 (0.99,1.02) | | 0.89 (0.55,1.44) | | | 0.91 (0.56,1.48) |  |
| Ovarian | 0.99 (0.96,1.02) | 0.99 (0.96,1.02) | | 1.22 (0.66,2.23) | | | 1.32 (0.71,2.43) |  |
| Post-menopausal breast | 1.00 (0.99,1.00) | 1.00 (0.99, 1.00) | | 1.10 (0.90,1.35) | | | 1.11 (0.91,1.37) |  |
| * Multivariable adjustment for baseline age, baseline WC, race, alcohol, smoking and HRT (in women).  * Degree of excess WC is the cumulative sum of the number of WC units ≥ 102 cm in men and ≥ 88 cm in women over the exposure period.  * Duration of excess WC is the cumulative sum of the duration of WC above the threshold over the exposure period.  **Abbreviations**: WC, waist circumference; OBR, obesity-related; NOBR, non-obesity related; CI, confidence interval; HR, hazard ratio; BMI, body mass index; MV, multivariable. | | | | | | | |  |

**Table S7: Comparison of the metrics by Akaike information criterion, ARIC cohort.**

|  |  | |  | |  | | **AIC** | | | | |
| --- | --- | --- | --- | --- | --- | --- | --- | --- | --- | --- | --- |
| **Characteristic** | **MV-adjusted waist circumference-years** | **MV-adjusted single**  **WC** | | **MV-adjusted single BMI** | | **MV-adjusted waist circumference -years with single WC** | | **MV-adjusted waist circumference -years with single BMI** | **MV-adjusted single WC -years with single BMI** | **MV-adjusted cumulative degree of excess WC** | **MV-adjusted cumulative duration of excess WC** |
| **Men** | | | | | | | | | | | |
| **All cancers** | 22833.26 | 22840.23 | | 22841.91 | | 22832.01 | | 22834.09 | 22840.98 | 22833.55 | 22838.93 |
| **OBR-cancers** | 4603.54 | 4605.24 | | 4606.09 | | 4605.46 | | 4605.33 | 4607.23 | 4603.32 | 4606.22 |
| **NOBR-cancers** | 18233.99 | 18237.95 | | 18238.40 | | 18231.47 | | 18234.02 | 18238.30 | 18234.30 | 18237.03 |
| **NOBR-cancers excluding lung and prostate** | 6475.66 | 6480.77 | | 6481.86 | | 6477.48 | | 6477.61 | 6482.76 | 6475.71 | 6477.09 |
| **Specific cancer sites** | | | | | | | | | | | |
| Colorectal | 1878.32 | 1879.79 | | 1879.74 | | 1879.73 | | 1879.89 | 1881.43 | 1878.21 | 1882.26 |
| Pancreas | 669.19 | 668.63 | | 668.30 | | 669.92 | | 670.41 | 670.25 | 669.14 | 667.96 |
| Kidney | 848.05 | 848.07 | | 848.05 | | 850.04 | | 850.05 | 850.04 | 848.05 | 847.91 |
| Lung | 3127.15 | 3123.29 | | 3114.64 | | 3100.52 | | 3118.07 | 3109.30 | 3127.20 | 3127.09 |
| Prostate | 8554.11 | 8554.10 | | 8554.02 | | 8555.83 | | 8556.04 | 8555.84 | 8554.10 | 8553.54 |
| **Women** | | | | | | | | | | | |
| **All cancers** | 19356.48 | 19350.28 | | 19351.62 | | 19353.20 | | 19353.20 | 19351.67 | 19356.00 | 19352.05 |
| **OBR-cancers** | 11734.82 | 11731.76 | | 11735.89 | | 11734.54 | | 11734.54 | 11733.66 | 11734.35 | 11732.16 |
| **NOBR-cancers** | 7590.19 | 7589.28 | | 7588.73 | | 7589.15 | | 7589.15 | 7590.64 | 7590.17 | 7589.40 |
| **NOBR-cancers excluding lung** | 5274.83 | 5273.59 | | 5271.75 | | 5270.80 | | 5270.80 | 5272.78 | 5274.80 | 5274.36 |
| **Specific cancer sites** | | | | | | | | | | | |
| Colorectal | 2056.47 | 2059.86 | | 2060.23 | | 2053.89 | | 2053.89 | 2061.29 | 2056.55 | 2052.64 |
| Pancreas | 652.78 | 652.57 | | 654.01 | | 653.55 | | 653.55 | 650.75 | 652.79 | 652.07 |
| Kidney | 644.09 | 641.65 | | 642.20 | | 644.20 | | 644.20 | 643.55 | 643.93 | 640.31 |
| Lung | 2276.55 | 2276.60 | | 2276.37 | | 2278.29 | | 2278.29 | 2277.34 | 2276.55 | 2276.43 |
| Endometrial | 1180.25 | 1174.33 | | 1175.88 | | 1176.58 | | 1176.58 | 1175.55 | 1179.64 | 1194.98 |
| Ovarian | 648.57 | 648.17 | | 648.58 | | 650.57 | | 650.57 | 649.46 | 648.55 | 647.44 |
| Post-menopausal breast cancer | 5620.77 | 5615.66 | | 5616.01 | | 5617.68 | | 5617.68 | 5617.34 | 5620.60 | 5616.53 |
| * Multivariable adjustment for baseline age, race, alcohol, smoking and hormone replacement therapy (in women).  **Abbreviations**: SE, standard error; OBR, obesity-related; NOBR, non-obesity related; BMI, body mass index; AIC, Akaike information criterion, WC, waist circumference. | | | | | | | | | | | |

**Table S8: Comparison of combined metrics using Harrell’s C-statistic, ARIC.**

| **Characteristic** | **WC-years with single WC** | **Difference in**  **c-statistic between**  **WC-years with**  **single WC and WC-years (95% CI)** | **WC -years with single BMI** | **Difference in c-statistic between WC-years with**  **single BMI vs WC-years (95% CI)** | **Single WC with single BMI** | **Difference in c-statistic between**  **single BMI with single WC and WC-years (95% CI)** |
| --- | --- | --- | --- | --- | --- | --- |
| **Men** | | | | | | |
| **All cancers** | 0.589  (0.579,  0.598) | 0.001  (-0.004,  0.007) | 0.589  (0.579,  0.598) | 0.001  (-0.005,  0.007) | 0.586 (0.577,  0.595) | -0.002   (-0.007,  0.003) |
| **OR-cancers** | 0.581  (0.562, 0.601) | 0.001  (-0.004,  0.005) | 0.581  (0.562,  0.601) | 0.000  (-0.003,  0.003) | 0.578 (0.558,  0.597) | -0.002  (-0.013,  0.010) |
| **NOR-cancers** | 0.595  (0.584, 0.607) | 0.001  (-0.006,  0.009) | 0.595  (0.584,  0.606) | 0.002   (-0.007,  0.010) | 0.592 (0.581,  0.603) | -0.000   (-0.007,  0.007) |
| **NOR-cancers excluding lung and prostate** | 0.600  (0.582, 0.618) | -0.000  (-0.001,  0.001) | 0.600  (0.582,  0.618) | -0.001  (-0.003,  0.002) | 0.600 (0.582,  0.618) | -0.000   (-0.008,  0.007) |
| Colorectal | 0.638 (0.607, 0.670) | 0.001  (-0.007,  0.009) | 0.638  (0.607,  0.670) | 0.000  (-0.009,  0.010) | 0.629  (0.599,  0.660) | -0.007   (-0.025,  0.019) |
| Pancreas | 0.643 (0.595, 0.695) | 0.014  (-0.029,  0.057) | 0.643  (0.595,  0.695) | 0.025   (-0.028,  0.079) | 0.644 (0.595,  0.695) | 0.026   (-0.018,  0.071) |
| Kidney | 0.653 (0.605, 0.705) | -0.000  (-0.001,  0.000) | 0.653  (0.606,  0.704) | -0.001  (-0.004,  0.002) | 0.653 (0.605,  0.704) | -0.000   (-0.006,  0.006) |
| Bladder | 0.637 (0.595, 0.682) | 0.003  (-0.025,  0.031) | 0.637  (0.593,  0.684) | -0.001  (-0.026,  0.024) | 0.638 (0.595,  0.685) | 0.002  (-0.025,  0.029) |
| Lung | 0.749 (0.727, 0.771) | 0.013  (-0.001,  0.028) | 0.749  (0.727,  0.771) | 0.027  (0.007,  0.047) | 0.739 (0.717,  0.761) | 0.023  (0.005,  0.041) |
| Prostate | 0.589 (0.574, 0.604) | 0.000  (-0.001,  0.002) | 0.589  (0.574,  0.604) | 0.001   (-0.002,  0.003) | 0.588 (0.574,  0.603) | 0.000  (-0.003,  0.003) |
| Metastatic Prostate | 0.541  (0.491, 0.597) | -0.031  (-0.130,  0.068) | 0.541  (0.491,  0.597) | -0.032  (-0.117,  0.053) | 0.588 (0.535,  0.646) | 0.012  (-0.068,  0.092) |
| **Women** | | | | | | |
| **All cancers** | 0.577 (0.566, 0.587) | 0.003  (-0.004,  0.011) | 0.577  (0.566,  0.587) | 0.002  (-0.005,  0.009) | 0.577 (0.566,  0.588) | 0.002  (-0.007,  0.011) |
| **OR-cancers** | 0.576 (0.563, 0.588) | 0.001  (-0.007,  0.009) | 0.576  (0.563,  0.588) | -0.001   (-0.007,  0.005) | 0.568 (0.556,  0.581) | -0.003  (-0.013,  0.008) |
| **NOR-cancers** | 0.629 (0.613, 0.646) | 0.002  (-0.003,  0.006) | 0.629  (0.614,  0.646) | 0.002  (-0.003,  0.007) | 0.629 (0.613,  0.645) | 0.001  (-0.002,  0.005) |
| **NOR-cancers excluding lung** | 0.592 (0.574, 0.610) | 0.005  (-0.005,  0.016) | 0.592  (0.574,  0.610) | 0.014   (-0.002,  0.030) | 0.589 (0.571,  0.607) | 0.013  (-0.001,  0.026) |
| Colorectal | 0.631  (0.601, 0.662) | 0.010  (-0.019,  0.039) | 0.631  (0.600,  0.662) | 0.012  (-0.023,  0.048) | 0.586 (0.556,  0.618) | -0.032  (-0.062,  -0.002) |
| Pancreas | 0.634  (0.575, 0.698) | 0.002  (-0.012,  0.016) | 0.634  (0.581,  0.692) | 0.012  (-0.023,  0.047) | 0.614 (0.565,0.667) | 0.028  (-0.032,  0.088) |
| Kidney | 0.655 (0.595,  0.720) | 0.009  (-0.027,  0.045) | 0.655  (0.596,  0.720) | 0.002  (-0.014,  0.018) | 0.655 (0.595,0.720) | 0.008  (-0.019,  0.035) |
| Lung | 0.761  (0.734,  0.790) | 0.001  (-0.001,  0.002) | 0.761  (0.733,  0.790) | 0.001  (-0.002,  0.003) | 0.761 (0.733,  0.789) | 0.003  (-0.002,  0.008) |
| Endometrial | 0.698 (0.652,  0.749) | 0.021  (-0.005,  0.048) | 0.698  (0.651,  0.749) | 0.012  (-0.011,  0.035) | 0.697 (0.650,  0.746) | 0.020  (-0.010,  0.049) |
| Ovarian | 0.621 (0.563,  0.684) | 0.006  (-0.036,  0.048) | 0.621  (0.567,  0.680) | -0.000  (-0.005,  0.004) | 0.620 (0.563,  0.683) | 0.003   (-0.040,  0.047) |
| Post-menopausal breast cancer | 0.583 (0.566,  0.602) | 0.006  (-0.010,  0.021) | 0.583  (0.566,  0.602) | 0.007  (-0.007,  0.021) | 0.584 (0.566,  0.602) | 0.007  (-0.005,  0.019) |
| * Multivariable adjustment for baseline age, race, alcohol, smoking and HRT (in women).  Key: Green – significant difference in C-statistic.  Abbreviations: SE, standard error; OBR, obesity-related; NOBR, non-obesity related; BMI, body mass index; CI, confidence interval; MV, multivariable-adjusted; WC, waist circumference. | | | | | | |

**Table S9: Hazard ratio of specific cancers related to waist circumference years, single WC and single BMI by race in (a) men and (b) women.**

**(a)**

| **Outcomes** | **Cancer events in White Men** | **Cancer events in Black Men** | **MV-adjusted HR (95% CI)** | | | | | | | | | |
| --- | --- | --- | --- | --- | --- | --- | --- | --- | --- | --- | --- | --- |
|  |  |  | **Waist circumference-years (per SD)** | | | **Baseline WC (per SD)** | | | **Baseline BMI (per SD)** | | | |
|  |  |  | **White** | **Black** | **Interaction of Black vs. White** | **White** | **Black** | **Interaction of Black vs. White** | **White** | **Black** | **Interaction of Black vs. White** |  |
| **Men** | | | | | | | | | | | | |
| All Cancers | 1,213 | 301 | 1.08 (1.03,1.14) | 1.10 (1.00,1.21) | 1.02 (0.91,1.14) | 1.04 (0.98,1.11) | 1.03 (0.93,1.14) | 0.99 (0.87,1.11) | 1.01 (0.96,1.07) | 1.03 (0.93,1.14) | 1.02 (0.90,1.14) |  |
| OBR-cancers | 248 | 58 | 1.14 (1.03,1.26) | 1.19 (0.99,1.43) | 1.04 (0.84,1.29) | 1.13 (0.99,1.28) | 1.19 (0.95,1.48) | 1.05 (0.82,1.36) | 1.12 (0.99,1.27) | 1.14 (0.91,1.43) | 1.02 (0.79,1.32) |  |
| NOBR-cancers | 965 | 243 | 1.06 (1.00,1.13) | 1.08 (0.96,1.20) | 1.01 (0.89,1.15) | 1.02 (0.96,1.09) | 0.99 (0.88,1.12) | 0.97 (0.85,1.11) | 0.99 (0.93,1.06) | 1.01 (0.90,1.13) | 1.02 (0.89,1.16) |  |
| NOBR cancers excluding lung and prostate | 373 | 53 | 1.14 (1.05,1.24) | 1.23 (1.02,1.48) | 1.07 (0.87,1.32) | 1.11 (1.00,1.23) | 1.15 (0.91,1.46) | 1.04 (0.8,1.34) | 1.10 (1.00,1.22) | 1.09 (0.85,1.39) | 0.99 (0.76,1.28) |  |
| **Specific cancer sites** | | | | | |  |  |  |  |  |  |  |
| Colorectal | 98 | 28 | 1.18 (1.02,1.37) | 1.37 (1.13,1.66) | 1.16 (0.91,1.48) | 1.15 (0.95,1.41) | 1.51 (1.16,1.98) | 1.31 (0.94,1.83) | 1.14 (0.94,1.39) | 1.52 (1.17,1.98) | 1.34 (0.97,1.85) |  |
| Kidney | 46 | 9 | 1.01 (0.75,1.35) | 0.64 (0.17,2.37) | 0.64 (0.17,2.43) | 0.96 (0.71,1.30) | 1.00 (0.55,1.82) | 1.03 (0.53,2.03) | 0.97 (0.71,1.31) | 0.94 (0.5,1.74) | 0.97 (0.49,1.93) |  |
| Bladder | 47 | 6 | 1.16 (0.91,1.47) | 0.38 (0.03,5.27) | 0.33 (0.02,4.60) | 1.26 (0.95,1.67) | 0.65 (0.28,1.53) | 0.52 (0.21,1.27) | 1.21 (0.92,1.59) | 0.85 (0.38,1.9) | 0.7 (0.3,1.64) |  |
| Pancreas | 33 | 12 | 1.11 (0.82,1.50) | 0.82 (0.35,1.91) | 0.74 (0.30,1.81) | 1.25 (0.89,1.75) | 0.88 (0.50,1.54) | 0.70 (0.36,1.35) | 1.32 (0.96,1.81) | 0.79 (0.44,1.41) | 0.6 (0.31,1.15) |  |
| Lung | 178 | 33 | 1.00 (0.87,1.16) | 1.17 (0.90,1.53) | 1.17 (0.87,1.58) | 0.83 (0.71,0.97) | 0.99 (0.72,1.37) | 1.19 (0.83,1.71) | 0.73 (0.62,0.86) | 0.89 (0.64,1.25) | 1.23 (0.84,1.78) |  |
| Prostate | 178 | 157 | 1.01 (0.92,1.11) | 0.98 (0.83,1.16) | 0.97 (0.80,1.17) | 1.03 (0.93,1.13) | 0.94 (0.81,1.09) | 0.91 (0.76,1.09) | 1.00 (0.91,1.11) | 0.99 (0.86,1.15) | 0.99 (0.83,1.18) |  |
| Metastatic Prostate | 22 | 12 | 0.66 (0.33,1.34) | 1.13 (0.73,1.77) | 1.71 (0.74,3.93) | 0.83 (0.53,1.30) | 1.18 (0.73,1.91) | 1.42 (0.74,2.74) | 0.82 (0.52,1.3) | 1.16 (0.72,1.87) | 1.42 (0.73,2.76) |  |
| * Multivariable adjustment for baseline age, race, alcohol, smoking and HRT (in women).  Abbreviations: OR, obesity-related; NOR, non-obesity related; CI, confidence interval; HR, hazard ratio; BMI, body mass index; MV, multivariable; WC, waist circumference. | | | | | | | | | | | |  |

| **Outcomes** | **Cancer events in White Women** | **Cancer events in Black Women** | **MV-adjusted HR (95% CI)** | | | | | | | | | |
| --- | --- | --- | --- | --- | --- | --- | --- | --- | --- | --- | --- | --- |
|  |  |  | **Waist circumference-years (per SD)** | | | **Baseline WC (per SD)** | | | **Baseline BMI (per SD)** | | | |
|  |  |  | **White** | **Black** | **Interaction of Black vs. White** | **White** | **Black** | **Interaction of Black vs. White** | **White** | **Black** | **Interaction of Black vs. White** |  |
| **Women** | | | | | | | | | | | | |
| All Cancers | 929 | 310 | 1.16 (1.09,1.23) | 1.08 (0.98,1.19) | 0.93 (0.83,1.04) | 1.17 (1.09,1.25) | 1.1 (0.99,1.22) | 0.94 (0.83,1.07) | 1.17 (1.09,1.25) | 1.08 (0.98,1.19) | 0.92 (0.82,1.04) |  |
| OBR-cancers | 539 | 212 | 1.24 (1.14,1.33) | 1.14 (1.02,1.27) | 0.92 (0.81,1.05) | 1.25 (1.15,1.36) | 1.14 (1.01,1.3) | 0.91 (0.79,1.07) | 1.24 (1.14,1.35) | 1.11 (0.98,1.25) | 0.89 (0.77,1.03) |  |
| NOBR-cancers | 390 | 98 | 1.05 (0.94,1.16) | 0.96 (0.8,1.16) | 0.92 (0.75,1.13) | 1.06 (0.95,1.17) | 1.01 (0.84,1.23) | 0.96 (0.77,1.19) | 1.07 (0.96,1.19) | 1.02 (0.85,1.23) | 0.96 (0.77,1.18) |  |
| NOBR cancers excluding lung | 266 | 70 | 1.05 (0.93,1.19) | 0.98 (0.8,1.22) | 0.94 (0.73,1.19) | 1.07 (0.94,1.21) | 1.06 (0.85,1.33) | 0.99 (0.77,1.28) | 1.12 (0.98,1.27) | 1.06 (0.85,1.31) | 0.95 (0.74,1.22) |  |
| **Specific cancer sites** | | | | | |  |  |  |  |  |  |  |
| Colorectal | 85 | 47 | 1.27 (1.06,1.53) | 1.04 (0.81,1.34) | 0.82 (0.6,1.11) | 1.12 (0.9,1.39) | 0.97 (0.73,1.29) | 0.87 (0.61,1.24) | 1.09 (0.87,1.37) | 0.94 (0.71,1.24) | 0.86 (0.6,1.23) |  |
| Pancreas | 22 | 21 | 1.26 (0.88,1.8) | 1.13 (0.8,1.6) | 0.9 (0.55,1.47) | 1.31 (0.86,1.99) | 1.11 (0.73,1.68) | 0.85 (0.47,1.52) | 1.26 (0.82,1.92) | 0.86 (0.56,1.34) | 0.69 (0.37,1.26) |  |
| Kidney | 26 | 15 | 1.47 (1.09,1.98) | 1.12 (0.73,1.73) | 0.76 (0.46,1.28) | 1.49 (1.03,2.15) | 1.39 (0.88,2.19) | 0.93 (0.52,1.67) | 1.43 (1,2.07) | 1.35 (0.9,2.03) | 0.94 (0.55,1.62) |  |
| Lung | 124 | 28 | 1.03 (0.86,1.24) | 0.91 (0.64,1.31) | 0.88 (0.59,1.32) | 1.03 (0.85,1.23) | 0.91 (0.63,1.31) | 0.88 (0.59,1.33) | 0.96 (0.79,1.17) | 0.93 (0.66,1.33) | 0.97 (0.65,1.45) |  |
| Endometrial | 62 | 14 | 1.57 (1.32,1.87) | 1.74 (1.28,2.38) | 1.11 (0.78,1.57) | 1.85 (1.47,2.31) | 1.88 (1.2,2.94) | 1.02 (0.62,1.68) | 1.78 (1.44,2.21) | 1.78 (1.22,2.59) | 1 (0.65,1.54) |  |
| Ovarian | 36 | 4 | 1.07 (0.75,1.52) | 0.78 (0.26,2.34) | 0.73 (0.23,2.3) | 1.13 (0.81,1.59) | 1.01 (0.38,2.66) | 0.89 (0.32,2.49) | 1.01 (0.7,1.45) | 1.28 (0.56,2.93) | 1.27 (0.52,3.14) |  |
| Post-menopausal breast | 267 | 90 | 1.15 (1.02,1.29) | 1.1 (0.92,1.31) | 0.95 (0.77,1.17) | 1.19 (1.06,1.35) | 1.13 (0.92,1.37) | 0.94 (0.75,1.19) | 1.21 (1.07,1.37) | 1.05 (0.87,1.27) | 0.86 (0.69,1.08) |  |
| * Multivariable adjustment for baseline age, race, alcohol, smoking and HRT (in women).  Abbreviations: OR, obesity-related; NOR, non-obesity related; CI, confidence interval; HR, hazard ratio; BMI, body mass index; MV, multivariable; WC, waist circumference. | | | | | | | | | | | |  |

(b)

**Table S10: Hazard ratio of specific cancers related to waist circumference years, single WC and single BMI by smoking in (a) men and (b) women.**

**(a)**

| **Outcomes** | **Cancer Incidence of ever smokers** | **Cancer**  **Incidence of**  **never smokers** | **MV-adjusted HR (95% CI)** | | | | | | | | |  |
| --- | --- | --- | --- | --- | --- | --- | --- | --- | --- | --- | --- | --- |
|  |  |  | **Waist circumference-years (per SD)** | | | **Baseline WC (per SD)** | | | **BMI (per SD)** | | |  |
|  |  |  | **Ever smoker** | **Never smoker** | **Interaction** | **Ever smoker** | **Never smoker** | **Interaction** | **Ever smoker** | **Never smoker** | **Interaction** |  |
| **Men** | | | | | | | | | | | | |
| **All Cancers** | 1,125 | 389 | 1.07 (1.02,1.13) | 1.11 (1.03,1.2) | 0.93 (0.83,1.05) | 1.02 (0.96,1.08) | 1.09 (0.99,1.20) | 0.93 (0.83,1.05) | 0.98 (0.92,1.04) | 1.14 (1.04,1.26) | 0.86 (0.76,0.96) |  |
| **OBR-cancers** | 212 | 94 | 1.09 (0.96,1.23) | 1.23 (1.09,1.39) | 0.83 (0.66,1.04) | 1.07 (0.94,1.23) | 1.30 (1.08,1.56) | 0.83 (0.66,1.04) | 1.04 (0.91,1.19) | 1.34 (1.11,1.61) | 0.78 (0.62,0.98) |  |
| **NOBR-cancers** | 913 | 295 | 1.07 (1.01,1.14) | 1.05 (0.95,1.17) | 0.98 (0.86,1.12) | 1.01 (0.95,1.08) | 1.03 (0.92,1.16) | 0.98 (0.86,1.12) | 0.97 (0.90,1.03) | 1.08 (0.97,1.22) | 0.89 (0.78,1.02) |  |
| **NOBR cancers excluding lung and prostate** | 319 | 107 | 1.15 (1.04,1.26) | 1.17 (1.03,1.34) | 0.95 (0.76,1.17) | 1.10 (0.99,1.23) | 1.17 (0.97,1.41) | 0.95 (0.76,1.17) | 1.07 (0.96,1.19) | 1.22 (1.01,1.47) | 0.88 (0.71,1.09) |  |
| **Specific cancer sites** | | | | | | | | | | | | |
| **Colorectal** | 88 | 38 | 1.06 (0.88,1.29) | 1.38 (1.21,1.56) | 0.54 (0.4,0.74) | 0.99 (0.8,1.22) | 1.83 (1.46,2.28) | 0.54 (0.40,0.74) | 0.98 (0.79,1.21) | 1.92 (1.51,2.44) | 0.51 (0.37,0.7) |  |
| **Kidney** | 32 | 23 | 0.98 (0.67,1.42) | 0.96 (0.61,1.5) | 1.00 (0.58,1.73) | 0.97 (0.68,1.38) | 0.97 (0.64,1.47) | 1.00 (0.58,1.73) | 0.92 (0.64,1.31) | 1.03 (0.68,1.58) | 0.89 (0.51,1.54) |  |
| **Bladder** | 47 | 6 | 1.15 (0.90,1.47) | 0.82 (0.26,2.55) | 1.53 (0.61,3.82) | 1.22 (0.92,1.61) | 0.80 (0.33,1.92) | 1.53 (0.61,3.82) | 1.19 (0.91,1.56) | 0.88 (0.36,2.16) | 1.36 (0.53,3.47) |  |
| **Pancreas** | 35 | 10 | 1.03 (0.73,1.45) | 1.12 (0.67,1.87) | 0.99 (0.50,1.95) | 1.12 (0.81,1.56) | 1.14 (0.63,2.07) | 0.99 (0.50,1.95) | 1.11 (0.80,1.53) | 1.30 (0.73,2.33) | 0.85 (0.44,1.65) |  |
| **Lung** | 202 | 9 | 1.01 (0.88,1.16) | 1.24 (0.81,1.89) | 0.60 (0.32,1.11) | 0.84 (0.73,0.97) | 1.41 (0.78,2.57) | 0.60 (0.32,1.11) | 0.73 (0.63,0.85) | 1.49 (0.82,2.69) | 0.49 (0.27,0.91) |  |
| **Prostate** | 392 | 179 | 1.03 (0.93,1.14) | 0.94 (0.81,1.11) | 1.09 (0.91,1.30) | 1.02 (0.93,1.13) | 0.94 (0.81,1.09) | 1.09 (0.91,1.30) | 1.01 (0.92,1.11) | 0.98 (0.85,1.14) | 1.03 (0.86,1.23) |  |
| **Metastatic prostate** | 24 | 10 | 0.55 (0.24,1.27) | 1.20 (0.83,1.73) | 0.56 (0.30,1.08) | 0.79 (0.52,1.20) | 1.40 (0.86,2.29) | 0.56 (0.30,1.08) | 0.79 (0.51,1.21) | 1.39 (0.82,2.36) | 0.57 (0.29,1.12) |  |
| Abbreviations: OBR, obesity-related; NOBR, non-obesity related; CI, confidence interval; HR, hazard ratio; BMI, body mass index; MV, multivariable; SD, standard deviation; N, number of cancer cases | | | | | | | | | | | |  |

**(b)**

| **Outcomes** | **Cancer Incidence of ever smokers** | **Cancer**  **Incidence of**  **never smokers** | **MV-adjusted HR (95% CI)** | | | | | | | | |  |
| --- | --- | --- | --- | --- | --- | --- | --- | --- | --- | --- | --- | --- |
|  |  |  | **Waist circumference-years (per SD)** | | | **Baseline WC (per SD)** | | | **BMI (per SD)** | | |  |
|  |  |  | **Ever smoker** | **Never smoker** | **Interaction** | **Ever smoker** | **Never smoker** | **Interaction** | **Ever smoker** | **Never smoker** | **Interaction** |  |
| **Women** | | | | | | | | | | | | |
| **All Cancers** | 663 | 576 | 1.1 (1.02,1.18) | 1.17 (1.09,1.25) | 0.91 (0.81,1.01) | 1.1 (1.02,1.18) | 1.21 (1.12,1.32) | 0.91 (0.81,1.01) | 1.09 (1.01,1.18) | 1.2 (1.1,1.29) | 0.91 (0.82,1.02) |  |
| **OBR-cancers** | 356 | 395 | 1.2 (1.1,1.32) | 1.2 (1.1,1.3) | 1.00 (0.87,1.15) | 1.22 (1.1,1.34) | 1.22 (1.1,1.34) | 1 (0.87,1.15) | 1.21 (1.1,1.33) | 1.18 (1.07,1.29) | 1.03 (0.9,1.18) |  |
| **NOBR-cancers** | 307 | 181 | 0.97 (0.86,1.09) | 1.1 (0.97,1.26) | 0.81 (0.67,0.97) | 0.97 (0.86,1.09) | 1.2 (1.04,1.39) | 0.81 (0.67,0.97) | 0.96 (0.85,1.08) | 1.23 (1.07,1.41) | 0.78 (0.65,0.93) |  |
| **NOBR cancers excluding lung** | 179 | 157 | 0.97 (0.83,1.13) | 1.1 (0.95,1.26) | 0.83 (0.67,1.03) | 0.98 (0.85,1.14) | 1.18 (1.01,1.38) | 0.83 (0.67,1.03) | 1.01 (0.87,1.18) | 1.21 (1.04,1.41) | 0.84 (0.68,1.03) |  |
| **Specific cancer sites** | | | | | | | | | | | | |
| **Colorectal** | 66 | 66 | 1.08 (0.85,1.37) | 1.24 (1.03,1.5) | 0.82 (0.59,1.15) | 0.96 (0.75,1.23) | 1.17 (0.92,1.49) | 0.82 (0.59,1.15) | 0.95 (0.73,1.22) | 1.1 (0.87,1.4) | 0.86 (0.61,1.2) |  |
| **Pancreas** | 18 | 25 | 0.93 (0.57,1.53) | 1.32 (1,1.76) | 0.8 (0.45,1.42) | 1.06 (0.68,1.66) | 1.33 (0.91,1.96) | 0.8 (0.45,1.42) | 0.88 (0.54,1.42) | 1.15 (0.79,1.68) | 0.76 (0.42,1.38) |  |
| **Kidney** | 21 | 20 | 1.42 (1.01,2) | 1.25 (0.87,1.79) | 1.17 (0.67,2.05) | 1.56 (1.07,2.27) | 1.33 (0.87,2.03) | 1.17 (0.67,2.05) | 1.57 (1.11,2.21) | 1.21 (0.8,1.82) | 1.3 (0.77,2.19) |  |
| **Lung** | 128 | 24 | 0.97 (0.81,1.17) | 1.14 (0.8,1.6) | 0.71 (0.46,1.09) | 0.95 (0.79,1.14) | 1.33 (0.9,1.97) | 0.71 (0.46,1.09) | 0.88 (0.73,1.07) | 1.36 (0.94,1.95) | 0.65 (0.43,0.98) |  |
| **Endometrial** | 33 | 43 | 1.63 (1.28,2.07) | 1.59 (1.31,1.93) | 1.07 (0.72,1.58) | 1.92 (1.43,2.57) | 1.8 (1.37,2.36) | 1.07 (0.72,1.58) | 1.89 (1.45,2.47) | 1.69 (1.32,2.17) | 1.12 (0.78,1.59) |  |
| **Ovarian** | 17 | 23 | 1.23 (0.79,1.91) | 0.88 (0.53,1.44) | 1.28 (0.68,2.4) | 1.28 (0.8,2.03) | 1 (0.64,1.55) | 1.28 (0.68,2.4) | 1.17 (0.72,1.91) | 0.95 (0.6,1.51) | 1.23 (0.64,2.38) |  |
| **Post-menopausal breast** | 166 | 191 | 1.16 (1.01,1.34) | 1.11 (0.98,1.27) | 1.04 (0.85,1.27) | 1.2 (1.04,1.39) | 1.15 (1,1.33) | 1.04 (0.85,1.27) | 1.21 (1.05,1.4) | 1.12 (0.97,1.29) | 1.09 (0.89,1.32) |  |
| **Abbreviations**: OBR, obesity-related; NOBR, non-obesity related; CI, confidence interval; HR, hazard ratio; BMI, body mass index; MV, multivariable; SD, standard deviation; N, number of cancer cases | | | | | | | | | | | |  |

**Table S11: Hazard ratio of cancers per standard deviation of waist circumference-years at Visit 4 and BMI at Visit 4, ARIC with additional adjustment for smoking pack-years.**

| **Outcomes** | **Cancer events** | **MV-adjusted HR (95% CI)** | | |
| --- | --- | --- | --- | --- |
|  |  | **Waist circumference-years (per SD)** | **Single WC (per SD)** | **Single BMI (per SD)** |
| **Men** | | | | |
| **All Cancers** | 1,514 | 1.08 (1.02,1.14) | 1.02 (0.99,1.04) | 1.03 (0.97,1.09) |
| **OR-cancers** | 306 | 1.16 (1.05,1.29) | 1.06 (1.01,1.11) | 1.16 (1.02,1.31) |
| **NOR-cancers** | 1,208 | 1.05 (0.99,1.12) | 1 (0.98,1.03) | 1 (0.94,1.07) |
| **NOR cancers excluding lung and prostate** | 426 | 1.16 (1.06,1.27) | 1.04 (1,1.09) | 1.11 (1,1.24) |
| **Specific cancer sites** | | | | |
| **Colorectal** | 126 | 1.26 (1.11,1.44) | 1.11 (1.04,1.2) | 1.32 (1.1,1.59) |
| **Pancreas** | 45 | 1.05 (0.76,1.46) | 1.05 (0.92,1.19) | 1.16 (0.84,1.61) |
| **Kidney** | 55 | 0.95 (0.69,1.31) | 0.98 (0.88,1.11) | 0.96 (0.7,1.31) |
| **Bladder** | 53 | 1.12 (0.85,1.46) | 1.06 (0.95,1.2) | 1.17 (0.87,1.58) |
| **Lung** | 211 | 0.94 (0.8,1.11) | 0.92 (0.86,0.98) | 0.73 (0.61,0.87) |
| **Prostate** | 571 | 1 (0.91,1.1) | 1 (0.97,1.04) | 1.02 (0.93,1.12) |
| **Metastatic Prostate** | 34 | 0.9 (0.57,1.41) | 0.99 (0.85,1.15) | 0.94 (0.63,1.39) |
| **Women** | | | | |
| **All Cancers** | 1,239 | 1.14 (1.08,1.2) | 1.15 (1.09,1.22) | 1.15 (1.09,1.22) |
| **OR-cancers** | 751 | 1.21 (1.13,1.29) | 1.22 (1.14,1.32) | 1.21 (1.12,1.3) |
| **NOR-cancers** | 488 | 1.03 (0.94,1.13) | 1.05 (0.95,1.15) | 1.06 (0.97,1.17) |
| **NOR cancers excluding lung** | 336 | 1.04 (0.93,1.16) | 1.07 (0.96,1.2) | 1.11 (0.99,1.24) |
| **Specific cancer sites** | | | | |
| **Colorectal** | 132 | 1.19 (1.01,1.39) | 1.07 (0.9,1.28) | 1.03 (0.85,1.23) |
| **Pancreas** | 43 | 1.17 (0.89,1.53) | 1.21 (0.89,1.65) | 1.06 (0.77,1.46) |
| **Kidney** | 41 | 1.32 (1.02,1.7) | 1.46 (1.09,1.95) | 1.42 (1.08,1.88) |
| **Lung** | 152 | 0.99 (0.84,1.18) | 0.98 (0.83,1.17) | 0.95 (0.8,1.14) |
| **Endometrial** | 76 | 1.72 (1.46,2.01) | 2.01 (1.63,2.48) | 1.92 (1.58,2.32) |
| **Ovarian** | 40 | 1.07 (0.76,1.49) | 1.15 (0.83,1.59) | 1.06 (0.76,1.5) |
| **Post-menopausal breast** | 357 | 1.12 (1.01,1.24) | 1.17 (1.05,1.3) | 1.16 (1.04,1.29) |
| * Multivariable adjustment for baseline age, race, alcohol, smoking and HRT (in women).  Abbreviations: OR, obesity-related; NOR, non-obesity related; CI, confidence interval; HR, hazard ratio; BMI, body mass index; MV, multivariable; WC, waist circumference. | | | | |

**Table S12: Hazard ratio of specific cancers related to waist circumference-years and baseline BMI by HRT status.**

| **Outcomes** | **Cancer Incidence ever HRT users** | **Cancer Incidence n ever HRT users** | **MV-adjusted HR (95% CI)** | | | | | | | | |
| --- | --- | --- | --- | --- | --- | --- | --- | --- | --- | --- | --- |
|  |  |  | **Waist circumference-years (per SD)** | | | **Baseline WC (per SD)** | | | **Baseline BMI (per SD)** | | |
|  |  |  | **Ever HRT user** | **Never HRT User** | **Interaction** | **Ever HRT user** | **Never HRT User** | **Interaction** | **Ever HRT user** | **Never HRT User** | **Interaction** |
| **Women** | | | | | | | | | | | |
| **All Cancers** | 599 | 640 | 1.15 (1.05,1.26) | 1.12 (1.05,1.2) | 0.99 (0.89,1.11) | 1.14 (1.05,1.24) | 1.15 (1.07,1.24) | 0.99 (0.89,1.11) | 1.13 (1.04,1.23) | 1.15 (1.07,1.23) | 0.99 (0.89,1.1) |
| **OBR-cancers** | 371 | 380 | 1.16 (1.04,1.3) | 1.22 (1.13,1.32) | 0.89 (0.78,1.03) | 1.14 (1.03,1.27) | 1.28 (1.17,1.4) | 0.89 (0.78,1.03) | 1.12 (1,1.24) | 1.25 (1.14,1.36) | 0.89 (0.78,1.03) |
| **NOBR-cancers** | 228 | 260 | 1.13 (0.98,1.31) | 0.97 (0.86,1.08) | 1.19 (0.99,1.42) | 1.15 (1.01,1.32) | 0.97 (0.86,1.1) | 1.19 (0.99,1.42) | 1.15 (1.01,1.33) | 0.99 (0.87,1.12) | 1.17 (0.98,1.4) |
| **NOBR cancers excluding lung** | 162 | 174 | 1.12 (0.94,1.32) | 0.99 (0.86,1.13) | 1.17 (0.94,1.46) | 1.16 (0.99,1.37) | 0.99 (0.86,1.15) | 1.17 (0.94,1.46) | 1.2 (1.02,1.41) | 1.03 (0.89,1.19) | 1.16 (0.94,1.44) |
| **Specific cancer sites** | | | | | | | | | | | |
| **Colorectal** | 66 | 66 | 1.06 (0.8,1.4) | 1.23 (1.03,1.47) | 0.85 (0.6,1.2) | 0.97 (0.74,1.26) | 1.14 (0.91,1.43) | 0.85 (0.60,1.2) | 0.94 (0.71,1.25) | 1.08 (0.87,1.35) | 0.87 (0.62,1.24) |
| **Pancreas** | 17 | 26 | 1.28 (0.79,2.09) | 1.14 (0.84,1.55) | 1 (0.54,1.84) | 1.2 (0.72,1.98) | 1.21 (0.84,1.73) | 1 (0.54,1.84) | 1.01 (0.59,1.74) | 1.04 (0.72,1.49) | 0.99 (0.53,1.87) |
| **Kidney** | 21 | 20 | 1.32 (0.83,2.11) | 1.33 (0.98,1.8) | 0.8 (0.44,1.42) | 1.26 (0.79,2) | 1.59 (1.1,2.29) | 0.8 (0.44,1.42) | 1.12 (0.69,1.82) | 1.57 (1.13,2.17) | 0.72 (0.41,1.27) |
| **Lung** | 66 | 86 | 1.17 (0.91,1.52) | 0.92 (0.74,1.14) | 1.21 (0.87,1.67) | 1.12 (0.87,1.43) | 0.93 (0.75,1.15) | 1.21 (0.87,1.67) | 1.04 (0.8,1.36) | 0.9 (0.72,1.12) | 1.17 (0.83,1.64) |
| **Endometrial** | 30 | 46 | 1.25 (0.82,1.89) | 1.68 (1.43,1.98) | 0.58 (0.38,0.91) | 1.29 (0.89,1.87) | 2.22 (1.74,2.84) | 0.58 (0.38,0.91) | 1.27 (0.88,1.85) | 2.04 (1.64,2.54) | 0.63 (0.41,0.96) |
| **Ovarian** | 30 | 17 | 1.35 (0.9,2.04) | 0.74 (0.4,1.36) | 1.35 (0.71,2.58) | 1.27 (0.83,1.95) | 0.95 (0.59,1.55) | 1.35 (0.71,2.58) | 1.07 (0.67,1.72) | 1.02 (0.63,1.66) | 1.07 (0.55,2.09) |
| **Post-menopausal breast cancer** | 183 | 174 | 1.12 (0.95,1.32) | 1.14 (1.01,1.29) | 0.99 (0.8,1.21) | 1.17 (1.01,1.35) | 1.18 (1.03,1.36) | 0.99 (0.8,1.21) | 1.16 (1,1.35) | 1.16 (1.01,1.33) | 1.00 (0.82,1.23) |
| **Abbreviations**: OBR, obesity-related; NOBR, non-obesity related; CI, confidence interval; HR, hazard ratio; BMI, body mass index; MV, multivariable; SD, standard deviation; N, number of cancer cases | | | | | | | | | | | |

**Sensitivity analysis using lower waist circumference thresholds**

**Table S13: Summary of the exposure metrics using lower waist circumference thresholds.**

| **Characteristic** | **Men** |
| --- | --- |
| **Baseline BMI, kg/m^2^** | 28.50 (4.50) |
| **Baseline WC, cm** | 103.00 (12.00) |
| **Follow up years** | 13.70 (6.10) |
| **Total cumulative waist circumference years** | 100.00 (86.00) |
| **Total cumulative waist circumference degree** | 47.00 (39.00) |
| **Total cumulative waist circumference duration** | 7.85 (2.60) |
|  | **Women** |
| **Baseline BMI, kg/m^2^** | 29.00 (6.00) |
| **Baseline WC, cm** | 101.00 (16.00) |
| **Follow up years** | 15.80 (5.20) |
| **Total cumulative waist circumference years** | 187.00 (133.00) |
| **Total cumulative waist circumference degree** | 88.00 (59.00) |
| **Total cumulative waist circumference duration** | 8.41 (1.74) |
| Mean (SD)  **Abbreviations**: N = number of participants; SD, standard deviation; BMI, body mass index, WC, waist circumference. | |

**Table S14: Incidence of cancer (events/1000 Person-Years) according to cumulative waist circumference years stratified by biological sex, race, smoking, HRT in the ARIC cohort using lower waist circumference thresholds.**

| **Men** | | | | | | | | | | | | | |
| --- | --- | --- | --- | --- | --- | --- | --- | --- | --- | --- | --- | --- | --- |
|  | **0 cm-years** | | | **≤100 cm-years** | | | **>100 cm-years** | | | **Baseline cohort** | | | |
|  | **N** | **PYFU** | **IR (95% CI)** | **N** | **PYFU** | **IR (95% CI)** | **N** | **PYFU** | **IR (95% CI)** | **N** | **PYFU** | **IR (95% CI)** |  |
| **Whole sample** | 760 | 144018.80 | 5.28  (4.90,5.66) | 487 | 91142.26 | 5.34   (4.86,5.82) | 267 | 41240.44 | 6.47 (5.69,7.26) | 1514 | 276401.5 | 5.48 (5.2,5.76) |  |
| **Race** | | | | | | | | | | | | | |
| **White** | 575 | 115509.90 | 4.98   (4.57,5.39) | 414 | 78456.35 | 5.28  (4.76,5.79) | 224 | 35864.09 | 6.25 (5.41,7.08) | 1213 | 229830.30 | 5.28 (4.98,5.58) |  |
| **Black** | 185 | 28508.96 | 6.49 (5.54,7.44) | 73 | 12685.91 | 5.75   (4.39,7.12) | 43 | 5376.353 | 8.00 (5.51,10.49) | 301 | 46571.23 | 6.46 (5.72,7.20) |  |
| **Smoking** | | | | | | | | | | | | | |
| **Ever** | 555 | 98286.49 | 5.65 (5.17,6.12) | 374 | 66675.37 | 5.61 (5.03,6.19) | 1945 | 30308.06 | 6.42 (5.50,7.34) | 1124 | 195269.90 | 5.76 (5.42,6.09) |  |
| **Never** | 205 | 45732.34 | 4.48 (3.85,5.1) | 113 | 24466.90 | 4.61 (3.74,5.49) | 73 | 10932.38 | 6.62 (5.05,8.2) | 390 | 81131.62 | 4.81 (4.32,5.29) |  |
| **Women** | | | | | | | | | | | | | |
| **Whole sample** | 29 | 13364.22 | 2.17 (1.34,3.00) | 198 | 76245.85 | 2.60 (2.23,2.97) | 1012 | 319663.30 | 3.17 (2.97,3.36) | 1239 | 409273.30 | 3.03 (2.86,3.20) |  |
| **Race** | | | | | | | | | | | | | |
| **White** | 25 | 12084.78 | 2.07 (1.21,2.93) | 171 | 65638.09 | 2.61 (2.21,3.00) | 733 | 223510.80 | 3.28 (3.04,3.52) | 929 | 301233.70 | 3.08 (2.88,3.28) |  |
| **Black** | 4 | 1279.44 | 3.13 (-0.45,6.70) | 27 | 10607.76 | 2.55 (1.53,3.56) | 279 | 96152.42 | 2.90 (2.56,3.25) | 310 | 108039.60 | 2.87 (2.55,3.19) |  |
| **Smoking** | | | | | | | | | | | | | |
| **Ever** | 20 | 7011.63 | 2.85 (1.52,4.18) | 123 | 38105.35 | 3.22 (2.63,3.80) | 520 | 142838.80 | 3.64 (3.33,3.96) | 663 | 187955.70 | 3.53 (3.26,3.80) |  |
| **Never** | 9 | 6352.60 | 1.42 (0.40,2.44) | 75 | 38140.49 | 1.98 (1.52,2.44) | 492 | 176824.50 | 2.78 (2.53,3.03) | 576 | 221317.60 | 2.60 (2.39,2.82) |  |
| **HRT** | | | | | | | | | | | | | |
| **Ever** | 18 | 7662.71 | 2.32 (1.17,3.47) | 124 | 48648.14 | 2.55 (2.09,3.01) | 459 | 154028.80 | 2.98 (2.7,3.25) | 601 | 210339.70 | 2.86 (2.63,3.09) |  |
| **Never** | 11 | 5701.51 | 1.96 (0.7,3.21) | 74 | 27597.70 | 2.68 (2.05,3.31) | 553 | 165634.40 | 3.34 (3.06,3.62) | 638 | 198933.60 | 3.21 (2.96,3.46) |  |
| **Abbreviations**: N, number of cancer events; PYFR, person-years of follow-up; IR, incidence rate of all cancers | | | | | | | | | | | | | |

**Table S15: Hazard ratio of specific cancers related to waist-circumference years in the ARIC cohort using lower waist circumference thresholds.**

| **Outcomes** | **Cancers** | **Waist circumference-years (per SD)** | | **Baseline WC**  **(per SD)** | | **Baseline BMI**  **(per SD)** | |
| --- | --- | --- | --- | --- | --- | --- | --- |
|  |  | **Age-adjusted HR (95% CI)** | **MV-adjusted HR**  **(95% CI)** | **Age-adjusted HR**  **(95% CI)** | **MV-adjusted HR**  **(95% CI)** | **Age-adjusted HR**  **(95% CI)** | **MV-adjusted HR**  **(95% CI)** |
|  |  | **Men** | | | | | |
| All Cancers | 1,514 | 1.07 (1.02,1.12) | 1.07 (1.02,1.12) | 1.04 (0.99,1.09) | 1.04 (0.99,1.10) | 1.03 (0.98,1.08) | 1.03 (0.98,1.08) |
| OBR-cancers | 306 | 1.13 (1.02,1.26) | 1.14 (1.03,1.26) | 1.13 (1.02,1.27) | 1.14 (1.03,1.28) | 1.13 (1.02,1.26) | 1.13 (1.02,1.26) |
| NOBR-cancers | 1,208 | 1.05 (1.00,1.11) | 1.05 (1.00,1.11) | 1.02 (0.96,1.07) | 1.02 (0.96,1.08) | 1.00 (0.94,1.06) | 1.00 (0.95,1.06) |
| NOBR-cancers excluding lung and prostate | 426 | 1.18 (1.09,1.29) | 1.17 (1.08,1.28) | 1.14 (1.04,1.25) | 1.12 (1.02,1.23) | 1.11 (1.01,1.22) | 1.11 (1.01,1.22) |
| **Specific cancer sites** | | | | | | | |
| Colorectal | 126 | 1.22 (1.05,1.42) | 1.23 (1.06,1.43) | 1.25 (1.06,1.48) | 1.27 (1.07,1.49) | 1.27 (1.08,1.49) | 1.26 (1.08,1.48) |
| Pancreas | 45 | 1.07 (0.80,1.43) | 1.08 (0.81,1.44) | 1.11 (0.83,1.48) | 1.13 (0.85,1.51) | 1.16 (0.88,1.54) | 1.16 (0.88,1.53) |
| Kidney | 55 | 1.01 (0.76,1.33) | 1.02 (0.78,1.35) | 0.96 (0.74,1.26) | 0.98 (0.75,1.28) | 0.97 (0.74,1.27) | 0.97 (0.74,1.27) |
| Lung | 53 | 0.99 (0.87,1.13) | 0.96 (0.84,1.10) | 0.89 (0.78,1.03) | 0.87 (0.75, 1.00) | 0.77 (0.66,0.89) | 0.77 (0.66,0.89) |
| Prostate | 571 | 0.98 (0.90,1.06) | 1.00 (0.92,1.08) | 0.97 (0.90,1.06) | 1.00 (0.93,1.09) | 1.01 (0.93,1.10) | 1.01 (0.93,1.10) |

| **Women** | | | | | | | |
| --- | --- | --- | --- | --- | --- | --- | --- |
| All Cancers | 1,239 | 1.12 (1.06,1.18) | 1.14 (1.08,1.21) | 1.13 (1.07,1.19) | 1.15 (1.09,1.22) | 1.12 (1.06,1.18) | 1.15 (1.08,1.21) |
| OBR-cancers | 751 | 1.21 (1.13,1.29) | 1.22 (1.14,1.31) | 1.21 (1.13,1.29) | 1.22 (1.13,1.31) | 1.18 (1.11,1.27) | 1.20 (1.12,1.29) |
| NOBR-cancers | 488 | 0.99 (0.90,1.09) | 1.03 (0.94,1.13) | 1.02 (0.93,1.11) | 1.05 (0.96,1.15) | 1.01 (0.92,1.10) | 1.06 (0.97,1.16) |
| NOBR-cancers excluding lung | 336 | 1.01 (0.90,1.12) | 1.04 (0.93,1.16) | 1.04 (0.93,1.16) | 1.07 (0.96,1.19) | 1.06 (0.95,1.18) | 1.11 (0.99,1.24) |
| **Specific cancer sites** | | | | |  |  |  |
| Colorectal | 132 | 1.21 (1.03,1.43) | 1.20 (1.01,1.42) | 1.08 (0.91,1.28) | 1.06 (0.89,1.26) | 1.06 (0.89,1.25) | 1.02 (0.86,1.22) |
| Pancreas | 43 | 1.34 (1.03,1.74) | 1.22 (0.92,1.61) | 1.33 (1.00,1.77) | 1.21 (0.90,1.62) | 1.19 (0.9,1.58) | 1.04 (0.76,1.40) |
| Kidney | 41 | 1.42 (1.07,1.87) | 1.41 (1.06,1.88) | 1.47 (1.11,1.94) | 1.46 (1.10,1.95) | 1.43 (1.10,1.85) | 1.42 (1.08,1.87) |
| Lung | 152 | 0.95 (0.81,1.12) | 1.00 (0.84,1.18) | 0.97 (0.82,1.14) | 1.00 (0.85,1.18) | 0.89 (0.75,1.06) | 0.96 (0.81,1.14) |
| Endometrial | 76 | 1.73 (1.43,2.09) | 1.76 (1.45,2.13) | 1.80 (1.48,2.18) | 1.85 (1.51,2.27) | 1.67 (1.00.4,2) | 1.78 (1.47,2.14) |
| Ovarian | 40 | 0.98 (0.70,1.38) | 1.12 (0.79,1.56) | 1.00 (0.74,1.37) | 1.12 (0.81,1.54) | 0.91 (0.65,1.26) | 1.05 (0.75,1.47) |
| Post-menopausal breast | 357 | 1.11 (1.00,1.23) | 1.14 (1.02,1.26) | 1.15 (1.04,1.27) | 1.18 (1.06,1.31) | 1.14 (1.03,1.26) | 1.17 (1.06,1.30) |
| * Multivariable adjustment for baseline age, race, alcohol, smoking and hormone replacement therapy (in women).  **Abbreviations**: OBR, obesity-related; NOBR, non-obesity related; CI, confidence interval; HR, hazard ratio; BMI, body mass index; MV, multivariable, WC, waist circumference. | | | | | | | |

**Table S16: Hazard ratio of specific cancers related to waist circumference years in the ARIC cohort using lower waist circumference thresholds.**

| **Outcomes** | **Cancers** | **Waist circumference-years**  **(per 100 cm-years)** | | **Baseline WC**  **(per 5 cm)** | | **Baseline BMI**  **(per 5 kg/m2)** | |
| --- | --- | --- | --- | --- | --- | --- | --- |
|  |  | **Age-adjusted HR**  **(95% CI)** | **MV-adjusted HR**  **(95% CI)** | **Age-adjusted HR (95% CI)** | **MV-adjusted HR**  **(95% CI)** | **Age-adjusted HR (95% CI)** | **MV-adjusted HR**  **(95% CI)** |
|  |  | **Men** | | | | | |
| All Cancers | 1,514 | 1.04 (1.01,1.07) | 1.04 (1.01,1.08) | 1.02 (0.99,1.04) | 1.02 (1.00,1.04) | 1.03 (0.97,1.09) | 1.03 (0.97,1.09) |
| OBR-cancers | 306 | 1.08 (1.01,1.16) | 1.09 (1.02,1.16) | 1.06 (1.01,1.11) | 1.06 (1.01,1.11) | 1.15 (1.02,1.30) | 1.15 (1.02,1.30) |
| NOBR-cancers | 1,208 | 1.03 (1.00,1.07) | 1.03 (1.00,1.07) | 1.01 (0.98,1.03) | 1.01 (0.98,1.03) | 1.00 (0.94,1.07) | 1.00 (0.94,1.07) |
| NOBR-cancers excluding lung and prostate | 426 | 1.11 (1.05,1.17) | 1.11 (1.05,1.17) | 1.06 (1.02,1.10) | 1.05 (1.01,1.1) | 1.13 (1.01,1.25) | 1.13 (1.01,1.25) |
| **Specific cancer sites** | | | | | | | |
| Colorectal | 126 | 1.13 (1.03,1.25) | 1.14 (1.04,1.25) | 1.10 (1.03,1.19) | 1.11 (1.03,1.19) | 1.31 (1.09,1.57) | 1.30 (1.09,1.57) |
| Pancreas | 45 | 1.04 (0.87,1.25) | 1.05 (0.88,1.26) | 1.05 (0.92,1.19) | 1.06 (0.93,1.20) | 1.19 (0.86,1.64) | 1.18 (0.86,1.63) |
| Kidney | 55 | 1.01 (0.84,1.20) | 1.01 (0.85,1.21) | 0.98 (0.88,1.11) | 0.99 (0.88,1.11) | 0.96 (0.71,1.31) | 0.96 (0.71,1.32) |
| Lung | 211 | 0.99 (0.92,1.08) | 0.98 (0.90,1.06) | 0.95 (0.90,1.01) | 0.94 (0.88, 1.00) | 0.74 (0.62,0.88) | 0.74 (0.62,0.88) |
| Prostate | 571 | 0.99 (0.93,1.04) | 1.00 (0.95,1.05) | 0.99 (0.95,1.03) | 1.00 (0.97,1.04) | 1.01 (0.92,1.11) | 1.01 (0.92,1.11) |

| **Women** | | | | | | | |
| --- | --- | --- | --- | --- | --- | --- | --- |
| All Cancers | 1,239 | 1.04 (1.02,1.07) | 1.05 (1.03,1.08) | 1.04 (1.02,1.06) | 1.04 (1.03,1.06) | 1.09 (1.05,1.14) | 1.11 (1.07,1.17) |
| OBR-cancers | 751 | 1.07 (1.05,1.10) | 1.08 (1.05,1.11) | 1.06 (1.04,1.08) | 1.06 (1.04,1.09) | 1.14 (1.08,1.21) | 1.16 (1.09,1.22) |
| NOBR-cancers | 488 | 1.00 (0.96,1.03) | 1.01 (0.98,1.05) | 1.01 (0.98,1.03) | 1.01 (0.99,1.04) | 1.01 (0.94,1.08) | 1.05 (0.97,1.13) |
| NOBR-cancers excluding lung | 336 | 1.00 (0.96,1.05) | 1.02 (0.97,1.06) | 1.01 (0.98,1.05) | 1.02 (0.99,1.06) | 1.05 (0.96,1.14) | 1.09 (0.99,1.18) |
| **Specific cancer sites** | | | | |  |  |  |
| Colorectal | 132 | 1.08 (1.01,1.15) | 1.07 (1.00,1.14) | 1.02 (0.97,1.08) | 1.02 (0.97,1.07) | 1.05 (0.91,1.20) | 1.02 (0.88,1.17) |
| Pancreas | 43 | 1.12 (1.01,1.24) | 1.08 (0.97,1.20) | 1.09 (1.00,1.19) | 1.06 (0.97,1.16) | 1.15 (0.92,1.44) | 1.03 (0.81,1.31) |
| Kidney | 41 | 1.14 (1.03,1.27) | 1.14 (1.02,1.27) | 1.13 (1.03,1.23) | 1.12 (1.03,1.23) | 1.33 (1.08,1.63) | 1.32 (1.06,1.65) |
| Lung | 152 | 0.98 (0.92,1.05) | 1.00 (0.94,1.07) | 0.99 (0.94,1.04) | 1.00 (0.95,1.05) | 0.92 (0.80,1.05) | 0.97 (0.84,1.11) |
| Endometrial | 76 | 1.23 (1.15,1.33) | 1.24 (1.15,1.34) | 1.2 (1.13,1.27) | 1.21 (1.14,1.29) | 1.51 (1.31,1.73) | 1.58 (1.36,1.83) |
| Ovarian | 40 | 0.99 (0.87,1.13) | 1.04 (0.92,1.19) | 1.00 (0.91,1.10) | 1.04 (0.94,1.14) | 0.92 (0.71,1.20) | 1.04 (0.79,1.36) |
| Post-menopausal breast cancer | 357 | 1.04 (1.00,1.08) | 1.05 (1.01,1.09) | 1.04 (1.01,1.08) | 1.05 (1.02,1.09) | 1.11 (1.02,1.20) | 1.14 (1.05,1.23) |
| * Multivariable adjustment for baseline age, baseline WC, race, alcohol, smoking and hormone replacement therapy (in women).  **Abbreviations**: OBR, obesity-related; NOBR, non-obesity related; CI, confidence interval; HR, hazard ratio; BMI, body mass index; MV, multivariable, WC, waist circumference. | | | | | | | |

**Table S17: Comparison of the waist circumference degree and duration per unit standard deviation using lower waist circumference thresholds.**

| **Outcomes** |  | **Cumulative degree of excess WC (per SD)** | | **Cumulative duration of excess WC (per SD)** | |
| --- | --- | --- | --- | --- | --- |
|  |  | **Age-adjusted HR (95% CI)** | **MV-adjusted HR (95% CI)** | **Age-adjusted HR (95% CI)** | **MV-adjusted HR (95% CI)** |
|  | **Cancers** | **Men** | | | |
| All Cancers | 1,514 | 1.15 (1.04,1.27) | 1.14 (1.04,1.26) | 0.97 (0.91,1.05) | 0.98 (0.91,1.06) |
| OBR-cancers | 306 | 1.09 (0.87,1.36) | 1.08 (0.86,1.36) | 0.94 (0.80,1.11) | 0.95 (0.81,1.12) |
| NOBR-cancers | 1,208 | 1.17 (1.04,1.31) | 1.16 (1.04,1.29) | 0.98 (0.91,1.06) | 0.99 (0.91,1.07) |
| NOBR-cancers excluding lung and prostate | 426 | 1.3 (1.08,1.56) | 1.32 (1.10,1.58) | 1.01 (0.88,1.16) | 1.00 (0.87,1.15) |
| **Specific cancer sites** | | | | | |
| Colorectal | 126 | 1.09 (0.78,1.53) | 1.08 (0.77,1.50) | 0.91 (0.71,1.16) | 0.93 (0.72,1.19) |
| Pancreas | 45 | 0.90 (0.48,1.69) | 0.88 (0.47,1.66) | 0.76 (0.51,1.13) | 0.79 (0.53,1.16) |
| Kidney | 55 | 1.20 (0.69,2.09) | 1.21 (0.70,2.10) | 1.77 (1.07,2.92) | 1.79 (1.08,2.98) |
| Lung | 211 | 1.36 (1.06,1.73) | 1.33 (1.03,1.71) | 0.97 (0.81,1.15) | 0.95 (0.8,1.13) |
| Prostate | 571 | 1.00 (0.84,1.19) | 0.98 (0.82,1.16) | 0.98 (0.87,1.10) | 1.01 (0.9,1.14) |

| **Women** | | | | | |
| --- | --- | --- | --- | --- | --- |
| All Cancers | 1,239 | 1.01 (0.90,1.14) | 1.04 (0.93,1.17) | 1.04 (0.93,1.15) | 1.06 (0.95,1.18) |
| OBR-cancers | 751 | 1.10 (0.95,1.27) | 1.11 (0.95,1.29) | 1.01 (0.87,1.16) | 1.01 (0.88,1.17) |
| NOBR-cancers | 488 | 0.90 (0.74,1.09) | 0.95 (0.79,1.16) | 1.09 (0.93,1.28) | 1.13 (0.96,1.33) |
| NOBR-cancers excluding lung | 336 | 0.89 (0.71,1.13) | 0.93 (0.74,1.17) | 1.06 (0.87,1.28) | 1.08 (0.89,1.31) |
| **Specific cancer sites** | | | |  |  |
| Colorectal | 132 | 1.67 (1.20,2.32) | 1.64 (1.18,2.29) | 1.13 (0.81,1.57) | 1.12 (0.80,1.57) |
| Pancreas | 43 | 1.25 (0.71,2.20) | 1.16 (0.65,2.07) | 1.43 (0.66,3.09) | 1.38 (0.65,2.96) |
| Kidney | 41 | 1.04 (0.56,1.92) | 1.06 (0.57,1.95) | 1.13 (0.53,2.40) | 1.16 (0.54,2.46) |
| Lung | 152 | 0.92 (0.65,1.30) | 1.00 (0.71,1.40) | 1.17 (0.87,1.57) | 1.25 (0.93,1.69) |
| Endometrial | 76 | 1.14 (0.73,1.76) | 1.12 (0.72,1.74) | 0.75 (0.46,1.22) | 0.76 (0.47,1.25) |
| Ovarian | 40 | 0.92 (0.45,1.87) | 1.02 (0.50,2.07) | 1.06 (0.60,1.89) | 1.09 (0.61,1.96) |
| Post-menopausal breast cancer | 357 | 0.91 (0.73,1.14) | 0.92 (0.74,1.16) | 0.98 (0.81,1.19) | 0.98 (0.81,1.20) |
| * Multivariable adjustment for baseline age, baseline WC, race, alcohol, smoking and HRT (in women).  * Degree of excess WC is the cumulative sum of the number of WC units ≥ 90 cm in men and ≥ 76 cm in women over the exposure period.  * Duration of excess WC is the cumulative sum of the duration of WC above the threshold over the exposure period.  **Abbreviations**: OBR, obesity-related; NOBR, non-obesity related; CI, confidence interval; HR, hazard ratio; BMI, body mass index; MV, multivariable, WC, waist circumference. | | | | | |

**Table S18: Comparison of the waist circumference degree and duration per 10 units and per 10 years respectively using predicted BMI from cohort with at least 3 BMI measurements in the ARIC cohort using lower waist circumference thresholds.**

| **Outcome** | **Cumulative degree of excess WC**  **(per 10 cm)** | | | | **Cumulative duration of excess WC**  **(per 10 years)** | | |  |
| --- | --- | --- | --- | --- | --- | --- | --- | --- |
|  | **Age-adjusted HR**  **(95% CI)** | | **MV-adjusted HR**  **(95% CI)** | | **Age-adjusted HR**  **(95% CI)** | **MV-adjusted HR**  **(95% CI)** | |  |
| **Men** | | | | | | | |  |
| **All Cancers** | 1.00 (1.00,1.01) | | 1.00 (1.00,1.01) | | 1.01 (0.94,1.09) | 1.02 (0.95,1.10) | |  |
| **OBR-cancers** | 1.01 (1.00,1.01) | | 1.01 (1.00,1.01) | | 1.07 (0.90,1.27) | 1.09 (0.91,1.29) | |  |
| **NOBR-cancers** | 1.00 (1.00,1.01) | | 1.00 (1.00,1.01) | | 1.00 (0.92,1.09) | 1.01 (0.93,1.10) | |  |
| **NOBR-cancers excluding lung and prostate** | 1.01 (1.00,1.02) | | 1.01 (1.00,1.01) | | 1.13 (0.98,1.32) | 1.11 (0.95,1.29) | |  |
| **Specific cancer sites** | | | | | | | |  |
| Colorectal | 1.01 (1.00,1.02) | | 1.01 (1.00,1.02) | | 1.12 (0.86,1.47) | 1.16 (0.88,1.52) | |  |
| Pancreas | 1.00 (0.99,1.02) | | 1.00 (0.99,1.02) | | 0.87 (0.58,1.31) | 0.91 (0.61,1.37) | |  |
| Kidney | 1.00 (0.98,1.02) | | 1.00 (0.99,1.02) | | 1.59 (0.91,2.77) | 1.63 (0.93,2.87) | |  |
| Lung | 1.00 (0.99,1.01) | | 1.00 (0.99,1.01) | | 0.89 (0.74,1.06) | 0.85 (0.71,1.02) | |  |
| Prostate | 1.00 (0.99, 1.00) | | 1.00 (0.99, 1.00) | | 0.96 (0.85,1.09) | 1.01 (0.89,1.14) | |  |
| **Women** | | | | | | | |  |
| **All Cancers** | 1.00 (1.00,1.01) | 1.00 (1.00,1.01) | | 1.21 (1.05,1.4) | | | 1.25 (1.08,1.45) | |
| **OBR-cancers** | 1.01 (1.00,1.01) | 1.01 (1.00,1.01) | | 1.27 (1.05,1.55) | | | 1.28 (1.06,1.56) | |
| **NOBR-cancers** | 1.00 (1.00, 1.00) | 1.00 (1.00, 1.00) | | 1.13 (0.91,1.4) | | | 1.22 (0.98,1.52) | |
| **NOBR-cancers excluding lung** | 1.00 (1.00, 1.00) | 1.00 (1.00,1.01) | | 1.11 (0.86,1.44) | | | 1.18 (0.91,1.53) | |
| **Specific cancer sites** | | | | | | | | |
| Colorectal | 1.01 (1.00,1.01) | 1.01 (1.00,1.01) | | 1.27 (0.80,2.00) | | | 1.23 (0.78,1.96) | |
| Pancreas | 1.01 (1.00,1.02) | 1.01 (1.00,1.02) | | 2.31 (0.75,7.10) | | | 1.92 (0.64,5.79) | |
| Kidney | 1.01 (1.00,1.02) | 1.01 (1.00,1.02) | | 2.04 (0.67,6.22) | | | 2.01 (0.66,6.15) | |
| Lung | 1.00 (0.99, 1.00) | 1.00 (0.99,1.01) | | 1.16 (0.78,1.71) | | | 1.30 (0.87,1.95) | |
| Endometrial | 1.02 (1.01,1.03) | 1.02 (1.01,1.03) | | 1.65 (0.79,3.44) | | | 1.70 (0.81,3.56) | |
| Ovarian | 1.00 (0.99,1.01) | 1.00 (0.99,1.02) | | 1.08 (0.50,2.33) | | | 1.26 (0.57,2.79) | |
| Post-menopausal breast cancer | 1.00 (1.00,1.01) | 1.00 (1.00,1.01) | | 1.16 (0.89,1.51) | | | 1.19 (0.91,1.55) | |
| * Multivariable adjustment for baseline age, baseline WC, race, alcohol, smoking and HRT (in women).  * Degree of excess WC is the cumulative sum of the number of WC units ≥ 90 cm in men and ≥ 76 cm in women over the exposure period.  * Duration of excess WC is the cumulative sum of the duration of WC above the threshold over the exposure period.  **Abbreviations**: OBR, obesity-related; NOBR, non-obesity related; CI, confidence interval; HR, hazard ratio; BMI, body mass index; MV, multivariable, WC, waist circumference. | | | | | | | | |

**Table S19: Comparison of the metrics calculated using WC predicted from the subgroup with at least 3 WC measurements by Akaike information criterion in the ARIC cohort using lower waist circumference thresholds.**

|  |  | |  | |  | | **AIC** | | | | |
| --- | --- | --- | --- | --- | --- | --- | --- | --- | --- | --- | --- |
| **Characteristic** | **MV-adjusted waist circumference-years** | **MV-adjusted single**  **WC** | | **MV-adjusted single BMI** | | **MV-adjusted waist circumference-years with single WC** | | **MV-adjusted waist circumference -years with single BMI** | **MV-adjusted single WC -years with single BMI** | **MV-adjusted cumulative degree of excess WC** | **MV-adjusted cumulative duration of excess WC** |
| **Men** | | | | | | | | | | | |
| **All cancers** | 22835.54 | 22840.23 | | 22841.91 | | 22833.58 | | 22835.58 | 22840.98 | 22835.62 | 22842.65 |
| **OBR-cancers** | 4605.25 | 4605.24 | | 4606.09 | | 4607.06 | | 4606.86 | 4607.23 | 4605.05 | 4610.01 |
| **NOBR-cancers** | 18234.96 | 18237.95 | | 18238.40 | | 18231.03 | | 18233.50 | 18238.30 | 18235.10 | 18238.36 |
| **NOBR-cancers excluding lung and prostate** | 6474.24 | 6480.77 | | 6481.86 | | 6474.84 | | 6474.56 | 6482.76 | 6474.32 | 6484.56 |
| **Specific cancer sites** | | | | | | | | | | | |
| Colorectal | 1880.59 | 1879.79 | | 1879.74 | | 1881.39 | | 1881.62 | 1881.43 | 1880.41 | 1886.11 |
| Pancreas | 669.03 | 668.63 | | 668.30 | | 670.04 | | 670.47 | 670.25 | 669.01 | 669.12 |
| Kidney | 848.08 | 848.07 | | 848.05 | | 849.72 | | 849.70 | 850.04 | 848.07 | 844.50 |
| Lung | 3126.95 | 3123.29 | | 3114.64 | | 3103.15 | | 3120.53 | 3109.3 | 3126.89 | 3124.41 |
| Prostate | 8554.11 | 8554.1 | | 8554.02 | | 8555.79 | | 8556.04 | 8555.84 | 8554.11 | 8554.08 |
| **Women** | | | | | | | | | | | |
| **All cancers** | 19353.25 | 19350.28 | | 19351.62 | | 19352.14 | | 19352.14 | 19351.67 | 19352.92 | 19363.42 |
| **OBR-cancers** | 11732.31 | 11731.76 | | 11735.89 | | 11733.05 | | 11733.05 | 11733.66 | 11732.04 | 11753.51 |
| **NOBR-cancers** | 7589.89 | 7589.28 | | 7588.73 | | 7590.26 | | 7590.26 | 7590.64 | 7589.86 | 7586.91 |
| **NOBR-cancers excluding lung** | 5274.49 | 5273.59 | | 5271.75 | | 5272.14 | | 5272.14 | 5272.78 | 5274.45 | 5273.38 |
| **Specific cancer sites** | | | | | | | | | | | |
| Colorectal | 2056.28 | 2059.86 | | 2060.23 | | 2052.96 | | 2052.96 | 2061.29 | 2056.41 | 2059.45 |
| Pancreas | 652.32 | 652.57 | | 654.01 | | 652.36 | | 652.36 | 650.75 | 652.33 | 652.28 |
| Kidney | 642.90 | 641.65 | | 642.20 | | 643.93 | | 643.93 | 643.55 | 642.79 | 645.94 |
| Lung | 2276.60 | 2276.60 | | 2276.37 | | 2277.98 | | 2277.98 | 2277.34 | 2276.60 | 2274.80 |
| Endometrial | 1180.05 | 1174.33 | | 1175.88 | | 1176.68 | | 1176.68 | 1175.55 | 1179.57 | 1205.12 |
| Ovarian | 648.26 | 648.17 | | 648.58 | | 650.11 | | 650.11 | 649.46 | 648.25 | 648.30 |
| Post-menopausal breast cancer | 5619.66 | 5615.66 | | 5616.01 | | 5617.97 | | 5617.97 | 5617.34 | 5619.52 | 5623.27 |
| * Multivariable adjustment for baseline age, race, alcohol, smoking and hormone replacement therapy (in women).  **Abbreviations**: SE, standard error; OBR, obesity-related; NOBR, non-obesity related; BMI, body mass index; AIC, Akaike information criterion, WC, waist circumference. | | | | | | | | | | | |

| **Table S20a and S20b: Comparison of the waist circumference-years metric, single WC and single BMI each at Visit 4 using Harrell’s C-statistic, ARIC,**  **using lower waist circumference thresholds.** | | | | | | | | |
| --- | --- | --- | --- | --- | --- | --- | --- | --- |
| 1. **Harrell’s C-statistic (95% CI)** | | | | | | | | |
| **Characteristic** | **WC-years** | **Single WC** | **Difference in c-statistic between single WC vs WC-years** | **Single**  **BMI** | **Difference in**  **C-statistic between**  **single BMI vs WC-years** | **Difference in C**  **-statistic between single BMI vs single WC** | **WC-years with single WC** | **Difference in c-statistic between WC-years with**  **single WC and WC-years** |
| **Men** | | | | | | | | |
| **All cancers** | 0.585  (0.576,  0.595) | 0.585 (0.575,  0.594) | -0.000  (-0.006,  0.006) | 0.584 (0.575,  0.594) | -0.001  (-0.009,  0.007) | -0.001  (-0.007,  0.006) | 0.589 (0.580,  0.599) | 0.002  (-0.004,  0.008) |
| **OBR-cancers** | 0.581 (0.562,  0.601) | 0.580 (0.560,  0.599) | -0.001  (-0.010,  0.007) | 0.578 (0.558,  0.598) | -0.003  (-0.014,  0.007) | -0.002  (-0.011,  0.007) | 0.580 (0.561,  0.600) | -0.001  (-0.006,  0.005) |
| **NOBR-cancers** | 0.594 (0.583,  0.606) | 0.593 (0.582,  0.605) | -0.001  (-0.005,  0.004) | 0.592 (0.581,  0.602) | -0.003   (-0.009,  0.004) | -0.002  (-0.009,  0.005) | 0.597 (0.586,  0.608) | 0.001  (-0.005,  0.005) |
| **NOBR-cancers excluding lung and prostate** | 0.603 (0.586,  0.621) | 0.600 (0.583,  0.618) | -0.003  (-0.009,  0.003) | 0.600 (0.582,  0.618) | -0.003  (-0.011,  0.005) | -0.000  (-0.005,  0.004) | 0.604 (0.586,  0.622) | 0.002  (-0.004,  0.007) |
| **Specific cancer sites** | | | | | | | | |
| Colorectal | 0.634 (0.604,  0.666) | 0.630 (0.600,  0.662) | -0.004  (-0.02,  0.008) | 0.629 (0.599,  0.661) | -0.005   (-0.023,  0.013) | -0.001  (0.014,  0.011) | 0.632 (0.602,  0.664) | -0.002  (-0.011,  0.006) |
| Kidney | 0.647  (0.598,  0.700) | 0.643 (0.595,  0.695) | -0.004  (-0.02,  0.01) | 0.644 (0.595,  0.696) | -0.003  (-0.015,  0.008) | 0.001  (-0.003,  0.004) | 0.650  (0.601,  0.703) | -0.001  (-0.016,  0.014) |
| Pancreas | 0.621 (0.580,  0.663) | 0.627  (0.590,  0.670) | 0.006  (-0.018,  0.030) | 0.638 (0.596,  0.684) | 0.017  (-0.017,  0.052) | 0.011  (-0.009,  0.031) | 0.634 (0.589,  0.682) | -0.002  (-0.040,  0.037) |
| Lung | 0.724  (0.703,  0.746) | 0.730  (0.710,  0.750) | 0.006  (-0.002,  0.014) | 0.739 (0.717,  0.761) | 0.015  (0.000,  0.029) | 0.009  (0.001,  0.017) | 0.747 (0.726,  0.769) | 0.008  (-0.006,  0.022) |
| Prostate | 0.588 (0.573,  0.603) | 0.588  (0.570,  0.600) | 0.000  (-0.001  0.001) | 0.588 (0.574,  0.603) | 0.000  (-0.002,  0.002) | 0.000  (-0.001,  0.001) | 0.589 (0.574,  0.604) | 0.000  (-0.001,  0.002) |
| **Women** | | | | | | | | |
| **All cancers** | 0.576 (0.565,  0.587) | 0.578 (0.567,  0.589) | 0.002  (-0.004,  0.008) | 0.577 (0.567,  0.588) | 0.0014  (-0.006,  0.008) | -0.000  (-0.010,  0.008) | 0.577 (0.567,  0.587) | 0.002  (-0.006,  0.009) |
| **OBR-cancers** | 0.578 (0.565,  0.590) | 0.575 (0.562,  0.587) | -0.003  (-0.014,  0.008) | 0.568 (0.556,  0.581) | -0.009  (-0.020,  0.002) | -0.006  (-0.015,  0.002) | 0.577 (0.564,  0.589) | 0.001  (-0.007,  0.008) |
| **NOBR-cancers** | 0.628 (0.612,  0.645) | 0.629 (0.613,  0.645) | 0.001  (-0.002,  0.003) | 0.629 (0.613,  0.646) | 0.001  (-0.002,  0.004) | 0.001  (-0.001,  0.002) | 0.630 (0.614,  0.647) | 0.002  (-0.002,  0.006) |
| **NOBR-cancers excluding lung** | 0.578 (0.560,  0.597) | 0.583 (0.565,  0.601) | 0.004  (-0.002,  0.011) | 0.589 (0.571,  0.607) | 0.011   (-0.000,  0.021) | 0.006  (0.000,  0.012) | 0.592 (0.574,  0.611) | 0.005  (-0.005,  0.015) |
| **Specific cancer sites** | | | | | | | | |
| Colorectal | 0.613 (0.583,  0.645) | 0.586 (0.556,  0.617) | -0.028  (-0.052,  -0.004) | 0.582  (0.552,  0.613) | -0.031   (-0.064,  0.002) | -0.004  (-0.018,  0.011) | 0.627 (0.597,  0.658) | 0.011  (-0.020,  0.042) |
| Pancreas | 0.624 (0.566,  0.688) | 0.627 (0.569,  0.690) | 0.003  (-0.016,  0.021) | 0.620 (0.564,  0.681) | -0.004  (-0.033,  0.025) | -0.007  (-0.038,  0.025) | 0.637 (0.578,  0.702) | 0.002  (-0.012,  0.016) |
| Kidney | 0.650 (0.593,  0.712) | 0.659 (0.600,  0.725) | 0.010  (-0.021,  0.040) | 0.652 (0.593,  0.716) | 0.002   (-0.014,  0.018) | -0.008  (-0.035,  0.020) | 0.653 (0.594,  0.717) | 0.010  (-0.020,  0.043) |
| Lung | 0.762  (0.734,  0.790) | 0.762 (0.735,  0.791) | 0.000  (-0.001,  0.002) | 0.762 (0.734,  0.791) | 0.000   (-0.001,  0.002) | -0.000  (-0.002,  0.000) | 0.762 (0.735,  0.791) | 0.001  (-0.001,  0.002) |
| Endometrial | 0.687 (0.638,  0.739) | 0.710 (0.663,  0.759) | 0.023  (-0.008,  0.054) | 0.696 (0.651,  0.745) | 0.010   (-0.020,  0.041) | -0.013  (-0.037,  0.010) | 0.699 (0.653,  0.748) | 0.021   (-0.004,  0.046) |
| Ovarian | 0.625 (0.571,  0.686) | 0.629 (0.573,  0.691) | 0.004  (-0.017,  0.024) | 0.625 (0.570,  0.685) | -0.000  (-0.012,  0.011) | -0.004  (-0.024,  0.016) | 0.626 (0.568,  0.689) | 0.007   (-0.034,  0.048) |
| Post-menopausal breast cancer | 0.561 (0.558,  0.595) | 0.582 (0.565,  0.601) | 0.006  (-0.006,  0.018) | 0.583  (0.565,  0.601) | 0.007  (-0.005,  0.019) | 0.000  (-0.008,  0.009) | 0.582 (0.565,  0.601) | 0.006  (-0.010,  0.021) |
| **Key: Green – significant difference in C-statistic.**  **Abbreviations**: SE, standard error; OBR, obesity-related; NOBR, non-obesity related; BMI, body mass index; CI, confidence interval; MV, multivariable-adjusted, WC, waist circumference. | | | | | | | | |

| 1. **Harrell’s C-statistic (95% CI)** | | | | | | | |
| --- | --- | --- | --- | --- | --- | --- | --- |
| **Characteristic** | **WC -years with single BMI** | **Difference in**  **c-statistic between WC-years with**  **single BMI vs WC-years** | **Single WC with single BMI** | **Difference in**  **c-statistic between**  **single BMI with single WC and WC-years** | **Cumulative degree of excess WC** | **Cumulative duration of excess WC** | **Difference in c-statistic between**  **cumulative WC duration and WC degree** |
| **All cancers** | 0.589 (0.579,  0.599) | 0.002  (-0.004,  0.007) | 0.591 (0.581,  0.600) | 0.000  (-0.006,  0.006) | 0.586 (0.577,  0.596) | 0.585  (0.575,  0.594) | -0.002  (-0.008,  0.005) |
| **OBR-cancers** | 0.580 (0.561,  0.600) | -0.001   (-0.004,  0.003) | 0.578 (0.558,  0.597) | -0.002  (-0.010,  0.007) | 0.581 (0.562,  0.601) | 0.568  (0.550,  0.587) | -0.013  (-0.032,  0.006) |
| **NOBR-cancers** | 0.597 (0.586,  0.608) | 0.002  (-0.004,  0.0076) | 0.593 (0.582,  0.604) | -0.000   (-0.006,  0.006) | 0.594 (0.583,  0.605) | 0.592  (0.582,  0.603) | -0.002  (-0.009,  0.006) |
| **NOBR-cancers excluding lung and prostate** | 0.604 (0.586,  0.622) | 0.000  (-0.004,  0.005) | 0.600 (0.582,  0.618) | -0.003  (-0.010,  0.003) | 0.603 (0.586,  0.621) | 0.596  (0.579,  0.614) | -0.007  (-0.019,  0.006) |
| **Specific cancer sites** | | | | | | | |
| Colorectal | 0.632 (0.602,  0.664) | -0.002  (-0.013,  0.010) | 0.629 (0.599,  0.660) | -0.004  (-0.018,  0.010) | 0.634 (0.604,  0.666) | 0.626  (0.596,  0.657) | -0.008  (-0.035,  0.018) |
| Kidney | 0.650 (0.602,  0.702) | 0.003  (-0.011,  0.018) | 0.644 (0.595,  0.695) | -0.003  (-0.014,  0.009) | 0.647 (0.598,  0.700) | 0.670  (0.623,  0.720) | 0.023  (-0.006,  0.052) |
| Pancreas | 0.634  (0.590,  0.680) | 0.013  (-0.040,  0.066) | 0.638 (0.595,  0.685) | 0.017  (-0.022,  0.056) | 0.621 (0.580,  0.666) | 0.572  (0.529,  0.618) | -0.049  (-0.105,  0.006) |
| Lung | 0.747  (0.730,  0.770) | 0.023  (0.003,  0.043) | 0.739 (0.717,  0.761) | 0.020  (0.005,  0.036) | 0.724 (0.703,  0.746) | 0.727  (0.707,  0.749) | 0.003  (-0.003,  0.009) |
| Prostate | 0.588  (0.570,  0.600) | 0.001  (-0.002,  0.003) | 0.588 (0.574,0.603) | 0.000  (-0.003,  0.003) | 0.588 (0.573,  0.603) | 0.588  (0.573,  0.603) | 0.000  (-0.001,  0.001) |
| **Women** | | | | | | | |
| **All cancers** | 0.578 (0.566,  0.588) | 0.002  (-0.003,  0.007) | 0.577 (0.567,  0.587) | 0.002  (-0.004,  0.008) | 0.576 (0.565,  0.587) | 0.577  (0.566,  0.588) | 0.001  (-0.009,  0.010) |
| **OBR-cancers** | 0.577  (0.564,  0.589) | -0.001  (-0.006,  0.004) | 0.568 (0.556,0.581) | -0.003   (-0.014,  0.007) | 0.578 (0.566,  0.591) | 0.576  (0.563,  0.588) | -0.002  (-0.015,  0.011) |
| **NOBR-cancers** | 0.630 (0.614,  0.646) | 0.002  (-0.003,  0.007) | 0.629 (0.613,  0.646) | 0.001  (-0.002,  0.005) | 0.628 (0.612,  0.645) | 0.629  (0.613,  0.646) | 0.001  (-0.001,  0.003) |
| **NOBR-cancers excluding lung** | 0.592  (0.574,  0.610) | 0.014  (-0.002,  0.023) | 0.589 (0.571,  0.607) | 0.012  (-0.000,  0.025) | 0.579 (0.560,  0.597) | 0.580  (0.562,  0.598) | 0.001  (-0.004,  0.006) |
| **Specific cancer sites** | | | | | | | |
| Colorectal | 0.627 (0.597,  0.658) | 0.014  (-0.025,  0.052) | 0.582 (0.552,  0.613) | -0.031  (-0.066,  0.003) | 0.612 (0.582,  0.644) | 0.614  (0.583,  0.646) | 0.001  (-0.026,  0.028) |
| Pancreas | 0.637 (0.583,  0.696) | 0.013  (-0.022,  0.048) | 0.620 (0.570,  0.674) | 0.028  (-0.031,  0.087) | 0.624 (0.566,  0.688) | 0.634  (0.576,  0.698) | 0.010  (-0.032,  0.052) |
| Kidney | 0.653 (0.594,  0.717) | 0.003   (-0.012,  0.018) | 0.652 (0.593,  0.717) | 0.009  (-0.017,  0.035) | 0.651 (0.594,  0.713) | 0.656  (0.593,  0.724) | 0.005  (-0.052,  0.062) |
| Lung | 0.762 (0.735,  0.791) | 0.001  (-0.001,  0.003) | 0.762 (0.734,  0.791) | 0.003  (-0.002,  0.009) | 0.762 (0.734,  0.790) | 0.763  (0.736,  0.792) | 0.002  (-0.001,  0.004) |
| Endometrial | 0.699 (0.652,  0.748) | 0.012  (-0.009,  0.033) | 0.696 (0.651,  0.745) | 0.019   (-0.010,  0.049) | 0.689 (0.641,  0.740) | 0.674  (0.632,  0.719) | -0.015  (-0.046,  0.017) |
| Ovarian | 0.626 (0.571,  0.686) | 0.000  (-0.005,  0.005) | 0.625 (0.5675,0.6888) | 0.001  (-0.042,  0.044) | 0.626 (0.571,  0.686) | 0.648  (0.596,  0.706) | 0.023  (-0.009,  0.054) |
| Post-menopausal breast cancer | 0.582 (0.565,  0.601) | 0.006  (-0.008,  0.020) | 0.583 (0.565,  0.601) | 0.007  (-0.005,  0.019) | 0.576 (0.559,  0.595) | 0.584  (0.566,  0.603) | 0.008  (-0.006,  0.022) |
| **Key: Green – significant difference in C-statistic.**  **Abbreviations**: SE, standard error; OBR, obesity-related; NOBR, non-obesity related; BMI, body mass index; CI, confidence interval; MV, multivariable-adjusted, WC, waist circumference. | | | | | | | |

**Sensitivity analysis using WC predicted from participants with at least 1 observed WC measurements**

**Table S21: Summary of the exposure metrics from participants with at least 1 observed WC measurement.**

| **Characteristic** | **Men (N = 4,568)** |
| --- | --- |
| **Baseline BMI, kg/m^2^** | 28.50 (4.50) |
| **Baseline WC, cm** | 103.00 (12.00) |
| **Follow up years** | 13.70 (6.10) |
| **Total cumulative waist circumference years** | 32.00 (57.00) |
| **Total cumulative waist circumference degree** | 16.00 (27.00) |
| **Total cumulative waist circumference duration** | 4.23 (4.04) |
|  | **Women (N = 5,691)** |
| **Baseline BMI, kg/m^2^** | 29.00 (6.00) |
| **Baseline WC, cm** | 101.00 (16.00) |
| **Follow up years** | 15.70 (5.20) |
| **Total cumulative waist circumference years** | 101.00 (113.00) |
| **Total cumulative waist circumference degree** | 48.00 (51.00) |
| **Total cumulative waist circumference duration** | 6.64 (3.41) |
| Mean (SD)  **Abbreviations**: N = number of participants; SD, standard deviation; BMI, body mass index, WC, waist circumference. | |

**Table S22: Incidence of cancer (events/1000 Person-Years) according to cumulative waist circumference years stratified by biological sex, race, smoking, HRT from participants with at least 1 observed WC measurement.**

| **Men** | | | | | | | | | | | | | |
| --- | --- | --- | --- | --- | --- | --- | --- | --- | --- | --- | --- | --- | --- |
|  | **0 Waist circumference-years** | | | **≤100 Waist circumference-years** | | | **>100 Waist circumference-years** | | | **Baseline cohort** | | | |
|  | **N** | **PYFU** | **IR (95% CI)** | **N** | **PYFU** | **IR (95% CI)** | **N** | **PYFU** | **IR (95% CI)** | **N** | **PYFU** | **IR (95% CI)** |  |
| **Whole sample** | 760 | 144018.8 | 5.28 (4.9,5.66) | 487 | 91142.26 | 5.34 (4.86,5.82) | 267 | 41240.44 | 6.47 (5.69,7.26) | 1514 | 276401.50 | 5.48 (5.20,5.76) |  |
| Race | | | | | | | | | | | | | |
| **White** | 575 | 115509.9 | 4.98 (4.57,5.39) | 414 | 78456.35 | 5.28 (4.76,5.79) | 224 | 35864.09 | 6.25 (5.41,7.08) | 1213 | 229830.30 | 5.28 (4.98,5.58) |  |
| **Black** | 185 | 28508.96 | 6.49 (5.54,7.44) | 73 | 12685.91 | 5.75 (4.39,7.12) | 43 | 5376.35 | 8 (5.51,10.49) | 301 | 46571.23 | 6.46 (5.72,7.20) |  |
| **Smoking** | | | | | | | | | | | | | |
| **Ever** | 555 | 98286.49 | 5.65 (5.17,6.12) | 374 | 66675.37 | 5.61 (5.03,6.19) | 195 | 30308.06 | 6.42 (5.5,7.34) | 1124 | 195269.90 | 5.76 (5.42,6.09) |  |
| **Never** | 205 | 45732.34 | 4.48 (3.85,5.10) | 113 | 24466.90 | 4.61 (3.74,5.49) | 72 | 10932.38 | 6.62 (5.05,8.2) | 390 | 81131.62 | 4.81 (4.32,5.29) |  |
| **Women** | | | | | | | | | | | | | |
| **Whole sample** | 231 | 94461.93 | 2.45 (2.12,2.77) | 383 | 135977.40 | 2.82 (2.53,3.10) | 632 | 182495.90 | 3.46 (3.19,3.74) | 1246 | 412935.30 | 3.02 (2.85,3.19) |  |
| **Race** | | | | | | | | | | | | | |
| **White** | 197 | 81181.09 | 2.43 (2.08,2.77) | 300 | 104031.80 | 2.88 (2.55,3.22) | 434 | 117254.50 | 3.7 (3.35,4.05) | 931 | 302467.40 | 3.08 (2.88,3.28) |  |
| **Black** | 34 | 13280.84 | 2.56 (1.66,3.46) | 83 | 31945.64 | 2.60 (2.02,3.17) | 198 | 65241.41 | 3.03 (2.60,3.47) | 315 | 110467.90 | 2.85 (2.53,3.17) |  |
| **Smoking** | | | | | | | | | | | | | |
| **Ever** | 141 | 47211.10 | 3 (2.49,3.50) | 212 | 63933.13 | 3.32 (2.86,3.77) | 311 | 78267.09 | 3.97 (3.53,4.42) | 664 | 189411.30 | 3.51 (3.24,3.78) |  |
| **Never** | 90 | 47250.83 | 1.9 (1.49,2.3) | 171 | 72044.27 | 2.37 (2.01,2.74) | 321 | 104228.90 | 3.08 (2.74,3.42) | 582 | 223524.00 | 2.6 (2.39,2.82) |  |
| **HRT** | | | | | | | | | | | | | |
| **Ever** | 140 | 57252.50 | 2.44 (2.03,2.85) | 197 | 74414.44 | 2.65 (2.27,3.03) | 264 | 80365.92 | 3.28 (2.88,3.68) | 600 | 212032.90 | 2.83 (2.60,3.06) |  |
| **Never** | 91 | 37209.43 | 2.46 (1.94,2.98) | 186 | 61562.96 | 3.02 (2.57,3.46) | 369 | 102130.00 | 3.61 (3.24,3.98) | 646 | 200902.40 | 3.21 (2.96,3.46) |  |
| **Abbreviations**: N, number of cancer events; PYFR, person-years of follow-up; IR, incidence rate of all cancers, WC, waist circumference. | | | | | | | | | | | | | |

**Table S23: Hazard ratio of specific cancers related to waist-circumference years derived using WC predicted from participants with at least 1 observed WC measurement.**

| **Outcomes** | **Cancers** | **Waist circumference-years**  **(per SD)** | | **Baseline WC**  **(per SD)** | | **Baseline BMI**  **(per SD)** | |
| --- | --- | --- | --- | --- | --- | --- | --- |
|  |  | **Age-adjusted HR (95% CI)** | **MV-adjusted HR**  **(95% CI)** | **Age-adjusted HR**  **(95% CI)** | **MV-adjusted HR**  **(95% CI)** | **Age-adjusted HR**  **(95% CI)** | **MV-adjusted HR**  **(95% CI)** |
|  |  | **Men** | | | | | |
| All Cancers | 1,521 | 1.07 (1.03,1.12) | 1.13 (1.06,1.21) | 1.03 (0.98,1.08) | 1.12 (0.99,1.26) | 1.02 (0.97,1.07) | 1.02 (0.97,1.07) |
| OBR-cancers | 307 | 1.14 (1.04,1.25) | 1.14 (0.99,1.30) | 1.13 (1.01,1.26) | 1.15 (0.88,1.51) | 1.12 (1.01,1.25) | 1.12(1.00,1.25) |
| NOBR-cancers | 1,214 | 1.06 (1.00,1.11) | 1.13 (1.05,1.22) | 1.01 (0.95,1.07) | 1.11 (0.97,1.27) | 0.99 (0.94,1.05) | 0.99 (0.94,1.05) |
| NOBR-cancers excluding lung and prostate | 427 | 1.15 (1.07,1.24) | 1.18 (1.05,1.32) | 1.13 (1.03,1.25) | 1.13 (0.90,1.43) | 1.11 (1.01,1.21) | 1.10(1.00,1.21) |
| **Specific cancer sites** | | | | | | | |
| Colorectal | 126 | 1.23 (1.09,1.38) | 1.16 (0.96,1.40) | 1.25 (1.06,1.48) | 1.13 (0.74,1.72) | 1.26 (1.08,1.49) | 1.26 (1.07,1.48) |
| Pancreas | 45 | 1.04 (0.78,1.39) | 0.88 (0.56,1.38) | 1.11 (0.83,1.48) | 0.94 (0.46,1.89) | 1.16 (0.88,1.54) | 1.15 (0.87,1.53) |
| Kidney | 55 | 0.96 (0.72,1.29) | 0.98 (0.65,1.48) | 0.96 (0.74,1.26) | 1.02 (0.53,1.95) | 0.97 (0.74,1.27) | 0.97 (0.74,1.27) |
| Bladder | 53 | 1.13 (0.90,1.42) | 1.02 (0.70,1.50) | 1.21 (0.93,1.57) | 1.15 (0.6,2.21) | 1.17 (0.90,1.52) | 1.15 (0.89,1.49) |
| Lung | 212 | 1.03 (0.91,1.17) | 1.47 (1.24,1.74) | 0.89 (0.77,1.02) | 1.54 (1.12,2.11) | 0.76 (0.65,0.89) | 0.76 (0.65,0.88) |
| Prostate | 575 | 0.98 (0.90,1.07) | 0.98 (0.87,1.11) | 0.97 (0.89,1.05) | 0.96 (0.79,1.18) | 1.00 (0.92,1.09) | 1.00 (0.92,1.09) |
| Metastatic prostate | 34 | 0.86 (0.56,1.31) | 0.82 (0.46,1.48) | 0.92 (0.65,1.30) | 1.07 (0.47,2.42) | 0.95 (0.68,1.35) | 0.95 (0.68,1.34) |

| **Women** | | | | | | | |
| --- | --- | --- | --- | --- | --- | --- | --- |
| All Cancers | 1,246 | 1.10 (1.05,1.16) | 1.03 (0.95,1.13) | 1.13 (1.07,1.19) | 1.10 (0.97,1.25) | 1.11 (1.06,1.17) | 1.14 (1.08,1.21) |
| OBR-cancers | 758 | 1.18 (1.11,1.26) | 1.12 (1.00,1.25) | 1.20 (1.12,1.29) | 1.20 (1.02,1.41) | 1.18 (1.1,1.26) | 1.19 (1.11,1.28) |
| NOBR-cancers | 488 | 0.98 (0.89,1.07) | 0.91 (0.78,1.05) | 1.02 (0.93,1.11) | 0.97 (0.79,1.19) | 1.01 (0.92,1.10) | 1.06 (0.97,1.16) |
| NOBR-cancers excluding lung | 336 | 0.99 (0.89,1.10) | 0.85 (0.71,1.02) | 1.04 (0.93,1.16) | 0.88 (0.69,1.13) | 1.06 (0.95,1.18) | 1.11 (0.99,1.24) |
| **Specific cancer sites** | | | | |  |  |  |
| Colorectal | 134 | 1.18 (1.01,1.36) | 1.47 (1.14,1.89) | 1.07 (0.90,1.26) | 1.23 (0.83,1.82) | 1.04 (0.88,1.23) | 1.00 (0.84,1.20) |
| Pancreas | 43 | 1.27 (1.00,1.61) | 1.42 (0.93,2.15) | 1.33 (1.00,1.77) | 2.22 (1.12,4.40) | 1.19 (0.90,1.58) | 1.03 (0.76,1.40) |
| Kidney | 42 | 1.34 (1.06,1.71) | 1.05 (0.67,1.64) | 1.48 (1.13,1.95) | 1.32 (0.67,2.60) | 1.44 (1.12,1.86) | 1.42 (1.08,1.85) |
| Lung | 152 | 0.94 (0.80,1.11) | 1.03 (0.79,1.35) | 0.97 (0.82,1.14) | 1.22 (0.83,1.79) | 0.89 (0.75,1.06) | 0.96 (0.81,1.14) |
| Endometrial | 77 | 1.61 (1.38,1.88) | 1.24 (0.92,1.67) | 1.79 (1.48,2.18) | 1.49 (0.89,2.49) | 1.67 (1.40,1.99) | 1.78 (1.48,2.14) |
| Ovarian | 40 | 0.93 (0.66,1.31) | 1.03 (0.59,1.79) | 1.00 (0.73,1.37) | 1.51 (0.70,3.23) | 0.90 (0.65,1.26) | 1.05 (0.75,1.47) |
| Post-menopausal breast | 359 | 1.09 (0.99,1.20) | 0.96 (0.81,1.13) | 1.15 (1.04,1.27) | 1.12 (0.88,1.42) | 1.13 (1.03,1.25) | 1.17 (1.06,1.30) |
| * Multivariable adjustment for baseline age, race, alcohol, smoking and hormone replacement therapy (in women).  **Abbreviations**: OBR, obesity-related; NOBR, non-obesity related; CI, confidence interval; HR, hazard ratio; BMI, body mass index; MV, multivariable, WC, waist circumference. | | | | | | | |

**Table S24: Hazard ratio of specific cancers related to waist circumference years derived using WC predicted from participants with at least 1 observed WC measurement.**

| **Outcomes** | **Cancers** | **Waist circumference-years (per 100 units)** | | **Baseline WC (per 5 cm)** | | **Baseline BMI (per 5 kg/m2)** | |
| --- | --- | --- | --- | --- | --- | --- | --- |
|  |  | **Age-adjusted HR (95% CI)** | **MV-adjusted HR**  **(95% CI)** | **Age-adjusted HR**  **(95% CI)** | **MV-adjusted HR**  **(95% CI)** | **Age-adjusted HR**  **(95% CI)** | **MV-adjusted HR**  **(95% CI)** |
|  |  | **Men** | | | | | |
| All Cancers | 1,521 | 1.08 (1.03,1.14) | 1.15 (1.07,1.24) | 1.03 (0.98,1.07) | 1.09 (0.98,1.22) | 1.02 (0.96,1.08) | 1.02 (0.97,1.08) |
| OBR-cancers | 307 | 1.16 (1.05,1.28) | 1.15 (0.99,1.34) | 1.11 (1.01,1.22) | 1.12 (0.89,1.42) | 1.14 (1.01,1.29) | 1.14 (1.01,1.29) |
| NOBR-cancers | 1,214 | 1.06 (1.00,1.12) | 1.15 (1.05,1.24) | 1.01 (0.96,1.06) | 1.08 (0.96,1.22) | 0.99 (0.93,1.06) | 1.00 (0.93,1.06) |
| NOBR-cancers excluding lung and prostate | 427 | 1.17 (1.07,1.27) | 1.2 (1.05,1.36) | 1.12 (1.03,1.21) | 1.11 (0.91,1.36) | 1.12 (1.01,1.25) | 1.12 (1.01,1.25) |
| **Specific cancer sites** | | | | | | | |
| Colorectal | 126 | 1.25 (1.10,1.43) | 1.18 (0.95,1.46) | 1.22 (1.05,1.41) | 1.11 (0.77,1.60) | 1.31 (1.09,1.57) | 1.30 (1.09,1.56) |
| Pancreas | 45 | 1.05 (0.76,1.44) | 0.86 (0.52,1.42) | 1.10 (0.85,1.41) | 0.93 (0.50,1.72) | 1.19 (0.86,1.64) | 1.18 (0.86,1.63) |
| Kidney | 55 | 0.96 (0.69,1.32) | 0.98 (0.62,1.55) | 0.97 (0.77,1.23) | 1.02 (0.58,1.8) | 0.96 (0.71,1.31) | 0.96 (0.70,1.32) |
| Bladder | 53 | 1.15 (0.89,1.48) | 1.02 (0.67,1.57) | 1.18 (0.94,1.48) | 1.12 (0.63,1.98) | 1.20 (0.89,1.61) | 1.18 (0.88,1.59) |
| Lung | 212 | 1.04 (0.90,1.19) | 1.54 (1.27,1.85) | 0.90 (0.79,1.02) | 1.44 (1.09,1.91) | 0.73 (0.62,0.87) | 0.73 (0.62,0.87) |
| Prostate | 575 | 0.98 (0.89,1.08) | 0.98 (0.86,1.12) | 0.97 (0.90,1.04) | 0.96 (0.81,1.14) | 1.00 (0.91,1.10) | 1.01 (0.92,1.10) |
| Metastatic Prostate | 34 | 0.85 (0.53,1.35) | 0.81 (0.42,1.54) | 0.93 (0.69,1.26) | 1.05 (0.51,2.14) | 0.95 (0.64,1.4) | 0.95 (0.65,1.40) |

| **Women** | | | | | | | |
| --- | --- | --- | --- | --- | --- | --- | --- |
| All Cancers | 1,246 | 1.05 (1.02,1.08) | 1.02 (0.97,1.06) | 1.08 (1.04,1.11) | 1.06 (0.98,1.15) | 1.09 (1.04,1.14) | 1.11 (1.06,1.16) |
| OBR-cancers | 758 | 1.09 (1.05,1.12) | 1.06 (1.00,1.12) | 1.12 (1.07,1.17) | 1.12 (1.01,1.24) | 1.14 (1.08,1.2) | 1.15 (1.09,1.22) |
| NOBR-cancers | 488 | 0.99 (0.95,1.03) | 0.95 (0.88,1.03) | 1.01 (0.96,1.07) | 0.98 (0.86,1.11) | 1.00 (0.93,1.08) | 1.05 (0.97,1.13) |
| NOBR-cancers excluding lung | 336 | 1.00 (0.95,1.05) | 0.92 (0.84,1.01) | 1.02 (0.96,1.09) | 0.93 (0.80,1.08) | 1.05 (0.96,1.14) | 1.08 (0.99,1.18) |
| **Specific cancer sites** | | | | | | | |
| Colorectal | 134 | 1.08 (1.01,1.17) | 1.21 (1.07,1.37) | 1.04 (0.94,1.15) | 1.13 (0.89,1.44) | 1.03 (0.90,1.18) | 1.00 (0.87,1.15) |
| Pancreas | 43 | 1.13 (1.00,1.27) | 1.19 (0.96,1.47) | 1.19 (1.00,1.42) | 1.63 (1.07,2.49) | 1.15 (0.92,1.44) | 1.03 (0.81,1.31) |
| Kidney | 42 | 1.16 (1.03,1.30) | 1.02 (0.82,1.28) | 1.27 (1.08,1.51) | 1.19 (0.78,1.80) | 1.34 (1.09,1.64) | 1.32 (1.06,1.63) |
| Lung | 152 | 0.97 (0.90,1.05) | 1.02 (0.89,1.16) | 0.98 (0.89,1.08) | 1.13 (0.89,1.43) | 0.91 (0.80,1.05) | 0.97 (0.84,1.11) |
| Endometrial | 77 | 1.27 (1.17,1.37) | 1.11 (0.96,1.29) | 1.43 (1.27,1.61) | 1.28 (0.93,1.75) | 1.5 (1.31,1.73) | 1.58 (1.36,1.83) |
| Ovarian | 40 | 0.97 (0.82,1.14) | 1.02 (0.77,1.34) | 1.00 (0.83,1.21) | 1.29 (0.81,2.05) | 0.92 (0.71,1.20) | 1.04 (0.79,1.36) |
| Post-menopausal breast | 359 | 1.04 (0.99,1.09) | 0.98 (0.90,1.06) | 1.09 (1.02,1.16) | 1.07 (0.92,1.24) | 1.10 (1.02,1.19) | 1.14 (1.05,1.23) |
| * Multivariable adjustment for baseline age, race, alcohol, smoking and hormone replacement therapy (in women).  **Abbreviations**: OBR, obesity-related; NOBR, non-obesity related; CI, confidence interval; HR, hazard ratio; BMI, body mass index; MV, multivariable, WC, waist circumference. | | | | | | | |

**Table S25: Comparison of the waist circumference degree and duration per unit standard deviation derived using WC predicted from participants with at least 1 observed WC measurement.**

| **Outcomes** |  | **Cumulative degree of excess WC (per SD)** | | **Cumulative duration of excess WC (per SD)** | |
| --- | --- | --- | --- | --- | --- |
|  |  | **Age-adjusted HR (95% CI)** | **MV-adjusted HR (95% CI)** | **Age-adjusted HR (95% CI)** | **MV-adjusted HR (95% CI)** |
|  | **Cancers** | **Men** | | | |
| All Cancers | 1,521 | 1.12 (1.05,1.21) | 1.12 (1.04,1.20) | 1.06 (0.98,1.14) | 1.05 (0.98,1.14) |
| OBR-cancers | 307 | 1.14 (0.98,1.32) | 1.13 (0.97,1.31) | 1.05 (0.89,1.25) | 1.05 (0.89,1.25) |
| NOBR-cancers | 1,214 | 1.12 (1.03,1.21) | 1.11 (1.03,1.21) | 1.06 (0.97,1.16) | 1.05 (0.97,1.15) |
| NOBR-cancers excluding lung and prostate | 427 | 1.15 (1.01,1.31) | 1.17 (1.03,1.33) | 1.15 (1.00,1.32) | 1.15 (1.00,1.33) |
| **Specific cancer sites** | | | | | |
| Colorectal | 126 | 1.19 (0.97,1.47) | 1.17 (0.95,1.44) | 1.03 (0.80,1.32) | 1.03 (0.80,1.32) |
| Pancreas | 45 | 0.93 (0.58,1.49) | 0.91 (0.56,1.48) | 1.22 (0.79,1.87) | 1.19 (0.77,1.85) |
| Kidney | 55 | 0.98 (0.63,1.53) | 0.97 (0.63,1.51) | 0.89 (0.57,1.39) | 0.90 (0.58,1.42) |
| Bladder | 53 | 0.97 (0.64,1.47) | 1.00 (0.66,1.53) | 1.9 (1.31,2.75) | 1.88 (1.30,2.72) |
| Lung | 212 | 1.27 (1.07,1.51) | 1.32 (1.10,1.59) | 1.24 (1.03,1.50) | 1.20 (0.99,1.45) |
| Prostate | 575 | 1.02 (0.90,1.16) | 0.99 (0.87,1.12) | 0.93 (0.81,1.06) | 0.92 (0.81,1.05) |
| Metastatic Prostate | 34 | 0.83 (0.44,1.56) | 0.79 (0.42,1.48) | 1.09 (0.64,1.85) | 1.07 (0.63,1.82) |

| **Women** | | | | | |
| --- | --- | --- | --- | --- | --- |
| All Cancers | 1,246 | 1.00 (0.90,1.11) | 1.01 (0.92,1.12) | 1.06 (0.97,1.16) | 1.08 (0.99,1.18) |
| OBR-cancers | 758 | 1.07 (0.95,1.22) | 1.08 (0.95,1.22) | 1.13 (1.01,1.27) | 1.14 (1.02,1.29) |
| NOBR-cancers | 488 | 0.88 (0.74,1.05) | 0.91 (0.77,1.08) | 0.98 (0.86,1.13) | 1.02 (0.89,1.17) |
| NOBR-cancers excluding lung | 336 | 0.88 (0.71,1.08) | 0.89 (0.73,1.10) | 0.97 (0.82,1.14) | 0.99 (0.84,1.17) |
| **Specific cancer sites** | | | | | |
| Colorectal | 134 | 1.45 (1.11,1.9) | 1.43 (1.09,1.88) | 1.60 (1.22,2.10) | 1.58 (1.20,2.08) |
| Pancreas | 43 | 1.10 (0.67,1.79) | 1.04 (0.63,1.72) | 1.29 (0.80,2.07) | 1.23 (0.77,1.96) |
| Kidney | 42 | 0.97 (0.58,1.64) | 0.98 (0.58,1.65) | 1.47 (0.86,2.52) | 1.48 (0.87,2.52) |
| Lung | 152 | 0.90 (0.66,1.23) | 0.94 (0.69,1.28) | 1.02 (0.80,1.30) | 1.08 (0.84,1.38) |
| Endometrial | 77 | 1.20 (0.85,1.70) | 1.19 (0.84,1.68) | 0.93 (0.63,1.38) | 0.95 (0.63,1.41) |
| Ovarian | 40 | 0.78 (0.40,1.52) | 0.85 (0.44,1.64) | 1.18 (0.72,1.94) | 1.26 (0.76,2.09) |
| Post-menopausal breast | 359 | 0.90 (0.74,1.10) | 0.91 (0.75,1.11) | 1.08 (0.92,1.27) | 1.10 (0.93,1.29) |
| * Multivariable adjustment for baseline age, baseline WC, race, alcohol, smoking and HRT (in women).  * Degree of excess WC is the cumulative sum of the number of WC units ≥ 102 cm in men and ≥ 88 cm in women over the exposure period.  * Duration of excess WC is the cumulative sum of the duration of WC above the threshold over the exposure period.  **Abbreviations**: OBR, obesity-related; NOBR, non-obesity related; CI, confidence interval; HR, hazard ratio; BMI, body mass index; MV, multivariable, WC, waist circumference. | | | | | |

**Table S26: Comparison of the waist circumference degree and duration per 10 units and per 10 years respectively derived using WC predicted from participants with at least 1 observed WC measurement.**

| **Outcome** | **Cumulative degree of excess WC (per 10 cm)** | | | | **Cumulative duration of excess WC (per 10 years)** | | | |
| --- | --- | --- | --- | --- | --- | --- | --- | --- |
|  | **Age-adjusted HR (95% CI)** | | **MV-adjusted HR (95% CI)** | | **Age-adjusted HR (95% CI)** | | **MV-adjusted HR (95% CI)** | |
| **Men** | | | | | | | | |
| **All Cancers** | 1.01 (1.00,1.02) | | 1.01 (1.00,1.02) | | 1.08 (0.98,1.19) | | 1.07 (0.97,1.18) | |
| **OBR-cancers** | 1.01 (1.00,1.03) | | 1.01 (1.00,1.03) | | 1.07 (0.86,1.34) | | 1.07 (0.86,1.34) | |
| **NOBR-cancers** | 1.01 (1.00,1.02) | | 1.01 (1.00,1.02) | | 1.08 (0.97,1.21) | | 1.07 (0.96,1.20) | |
| **NOBR-cancers excluding lung and prostate** | 1.01 (1.00,1.03) | | 1.02 (1.00,1.03) | | 1.20 (1.00,1.45) | | 1.20 (1.00,1.45) | |
| **Specific cancer sites** | | | | | | | | |
| Colorectal | 1.02 (1.00,1.04) | | 1.02 (0.99,1.04) | | 1.04 (0.74,1.44) | | 1.03 (0.75,1.44) | |
| Pancreas | 0.99 (0.94,1.04) | | 0.99 (0.94,1.04) | | 1.29 (0.73,2.28) | | 1.26 (0.71,2.24) | |
| Kidney | 1.00 (0.95,1.04) | | 1.00 (0.95,1.04) | | 0.86 (0.48,1.55) | | 0.88 (0.49,1.58) | |
| Bladder | 1.00 (0.96,1.04) | | 1.00 (0.96,1.04) | | 2.32 (1.43,3.77) | | 2.28 (1.4,3.71) | |
| Lung | 1.03 (1.01,1.04) | | 1.03 (1.01,1.05) | | 1.33 (1.03,1.70) | | 1.27 (0.99,1.62) | |
| Prostate | 1.00 (0.99,1.02) | | 1.00 (0.99,1.01) | | 0.91 (0.76,1.08) | | 0.90 (0.76,1.07) | |
| Metastatic prostate | 0.98 (0.92,1.05) | | 0.98 (0.91,1.04) | | 1.12 (0.56,2.24) | | 1.09 (0.54,2.19) | |
| **Women** | | | | | | | | |
| **All Cancers** | 1.00 (1.00,1.00) | 1.00 (1.00,1.01) | | 1.07 (0.97,1.20) | | 1.10 (0.99,1.23) | |  |
| **OBR-cancers** | 1.00 (1.00,1.01) | 1.00 (1.00,1.01) | | 1.17 (1.01,1.34) | | 1.18 (1.02,1.36) | |  |
| **NOBR-cancers** | 0.99 (0.99,1.00) | 1.00 (0.99,1.00) | | 0.98 (0.83,1.15) | | 1.03 (0.87,1.21) | |  |
| **NOBR-cancers excluding lung** | 0.99 (0.98,1.00) | 0.99 (0.99,1.00) | | 0.96 (0.78,1.17) | | 0.99 (0.81,1.21) | |  |
| **Specific cancer sites** | | | | | | | |  |
| Colorectal | 1.02 (1.00,1.03) | 1.02 (1.00,1.03) | | 1.77 (1.27,2.47) | | 1.74 (1.25,2.43) | |  |
| Pancreas | 1.00 (0.98,1.03) | 1.00 (0.98,1.03) | | 1.36 (0.76,2.42) | | 1.29 (0.73,2.28) | |  |
| Kidney | 1.00 (0.97,1.02) | 1.00 (0.97,1.02) | | 1.60 (0.83,3.09) | | 1.61 (0.84,3.09) | |  |
| Lung | 1.00 (0.98,1.01) | 1.00 (0.98,1.01) | | 1.02 (0.76,1.37) | | 1.10 (0.81,1.48) | |  |
| Endometrial | 1.01 (0.99,1.03) | 1.01 (0.99,1.02) | | 0.91 (0.56,1.48) | | 0.94 (0.57,1.52) | |  |
| Ovarian | 0.99 (0.96,1.02) | 0.99 (0.96,1.02) | | 1.22 (0.67,2.24) | | 1.32 (0.71,2.45) | |  |
| Post-menopausal breast | 1.00 (0.99,1.00) | 1.00 (0.99,1.00) | | 1.1 (0.90,1.34) | | 1.12 (0.91,1.37) | |  |
| * Multivariable adjustment for baseline age, baseline WC, race, alcohol, smoking and HRT (in women).  * Degree of excess WC is the cumulative sum of the number of WC units ≥ 102 cm in men and ≥ 88 cm in women over the exposure period.  * Duration of excess WC is the cumulative sum of the duration of WC above the threshold over the exposure period.  **Abbreviations**: OBR, obesity-related; NOBR, non-obesity related; CI, confidence interval; HR, hazard ratio; BMI, body mass index; MV, multivariable, WC, waist circumference. | | | | | | | |  |

**Table S27: Comparison of the metrics calculated using WC predicted from the subgroup with at least 3 WC measurements by Akaike information criterion derived using WC predicted from participants with at least 1 observed WC measurement.**

|  |  | |  | |  | | **AIC** | | | | |
| --- | --- | --- | --- | --- | --- | --- | --- | --- | --- | --- | --- |
| **Characteristic** | **MV-adjusted waist circumference-years** | **MV-adjusted single**  **WC** | | **MV-adjusted single BMI** | | **MV-adjusted waist circumference -years with single WC** | | **MV-adjusted waist circumference -years with single BMI** | **MV-adjusted single WC -years with single BMI** | **MV-adjusted cumulative degree of excess WC** | **MV-adjusted cumulative duration of excess WC** |
| **Men** | | | | | | | | | | | |
| **All cancers** | 22951.11 | 22958.44 | | 22959.77 | | 22948.77 | | 22951.18 | 22959.12 | 22951.42 | 22956.73 |
| **OBR-cancers** | 4621.81 | 4623.87 | | 4624.80 | | 4623.80 | | 4623.71 | 4625.87 | 4621.60 | 4624.48 |
| **NOBR-cancers** | 18333.79 | 18337.74 | | 18337.90 | | 18330.24 | | 18332.96 | 18338.12 | 18334.10 | 18336.77 |
| **NOBR-cancers excluding lung and prostate** | 6494.28 | 6499.64 | | 6500.65 | | 6496.03 | | 6496.18 | 6501.63 | 6494.34 | 6495.81 |
| **Specific cancer sites** | | | | | | | | | | | |
| Colorectal | 1880.20 | 1881.69 | | 1881.64 | | 1881.62 | | 1881.77 | 1883.33 | 1880.09 | 1884.20 |
| Pancreas | 669.66 | 669.10 | | 668.77 | | 670.40 | | 670.88 | 670.72 | 669.61 | 668.48 |
| Kidney | 848.82 | 848.84 | | 848.82 | | 850.81 | | 850.82 | 850.81 | 848.82 | 848.69 |
| Bladder | 816.59 | 816.08 | | 816.21 | | 818.19 | | 818.08 | 818.07 | 816.54 | 808.65 |
| Lung | 3146.88 | 3142.39 | | 3133.67 | | 3119.08 | | 3136.47 | 3129.14 | 3146.93 | 3146.78 |
| Prostate | 8615.80 | 8615.81 | | 8615.81 | | 8617.69 | | 8617.80 | 8617.59 | 8615.78 | 8615.11 |
| Metastatic Prostate | 525.26 | 525.67 | | 525.63 | | 527.16 | | 527.03 | 527.62 | 525.29 | 525.70 |
| **Women** | | | | | | | | | | | |
| **All cancers** | 19482.64 | 19476.64 | | 19478.41 | | 19479.86 | | 19479.86 | 19478.18 | 19482.17 | 19477.82 |
| **OBR-cancers** | 11853.21 | 11850.49 | | 11855.21 | | 11853.36 | | 11853.36 | 11852.46 | 11852.75 | 11850.08 |
| **NOBR-cancers** | 7595.76 | 7594.87 | | 7594.32 | | 7594.66 | | 7594.66 | 7596.23 | 7595.74 | 7594.97 |
| **NOBR-cancers excluding lung** | 5279.23 | 5278.02 | | 5276.23 | | 5275.18 | | 5275.18 | 5277.26 | 5279.21 | 5278.75 |
| **Specific cancer sites** | | | | | | | | | | | |
| Colorectal | 2088.78 | 2091.68 | | 2091.91 | | 2085.54 | | 2085.54 | 2092.84 | 2088.85 | 2084.65 |
| Pancreas | 653.68 | 653.46 | | 654.88 | | 654.44 | | 654.44 | 651.64 | 653.69 | 652.81 |
| Kidney | 658.16 | 656.03 | | 656.59 | | 658.55 | | 658.55 | 657.94 | 657.99 | 654.75 |
| Lung | 2277.61 | 2277.66 | | 2277.43 | | 2279.37 | | 2279.37 | 2278.44 | 2277.61 | 2277.48 |
| Endometrial | 1196.52 | 1191.84 | | 1193.41 | | 1193.54 | | 1193.54 | 1193.02 | 1195.93 | 1212.30 |
| Ovarian | 648.84 | 648.44 | | 648.85 | | 650.84 | | 650.84 | 649.73 | 648.82 | 647.67 |
| Post-menopausal breast cancer | 5660.48 | 5655.28 | | 5655.87 | | 5657.60 | | 5657.60 | 5657.05 | 5660.30 | 5656.11 |
| * Multivariable adjustment for baseline age, race, alcohol, smoking and HRT (in women).  **Abbreviations**: SE, standard error; OBR, obesity-related; NOBR, non-obesity related; BMI, body mass index; AIC, Akaike information criterion, WC, waist circumference. | | | | | | | | | | | |

| **Table S28a S28b: Comparison of the waist circumference-years metric, single WC and single BMI each at Visit 4 using Harrell’s C-statistic**  **derived using WC predicted from participants with at least 1 observed WC measurement.** | | | | | | | | |
| --- | --- | --- | --- | --- | --- | --- | --- | --- |
| 1. **Harrell’s C-statistic (95% CI)** | | | | | | | | |
| **Characteristic** | **WC-years** | **Single WC** | **Difference in c-statistic between single WC vs WC-years** | **Single**  **BMI** | **Difference in c-statistic between**  **single BMI vs WC-years** | **Difference in c-statistic between single BMI vs single WC** | **WC-years with single WC** | **Difference in c-statistic between WC-years with**  **single WC and WC-years** |
| **Men** | | | | | | | | |
| **All cancers** | 0.587  (0.578,  0.597) | 0.584 (0.574,  0.593) | -0.003  (-0.009,  0.002) | 0.583  (0.574,  0.593) | -0.004  (-0.010,  0.002) | -0.000  (-0.007,  0.006) | 0.577  (0.568,  0.586) | 0.001   (-0.003,  0.004) |
| **OBR-cancers** | 0.582  (0.562,  0.602) | 0.580 (0.561,  0.599) | -0.002  (-0.013,  0.009) | 0.578  (0.558,  0.598) | -0.004  (-0.015,  0.008) | -0.002  (-0.010,  0.006) | 0.582 (0.562,  0.602) | 0.001  (-0.003,  0.004) |
| **NOBR-cancers** | 0.593 (0.583,  0.604) | 0.592 (0.581,  0.603) | -0.001  (-0.010,  0.007) | 0.592  (0.581,  0.604) | -0.001  (-0.007,  0.005) | 0.001  (-0.007,  0.007) | 0.595 (0.584,  0.606) | 0.001  (-0.006,  0.008) |
| **NOBR-cancers excluding lung and prostate** | 0.600  (0.582,  0.618) | 0.600 (0.582,  0.618) | -0.000  (-0.008,  0.007) | 0.600  (0.58,  0.618) | -0.001  (-0.008,  0.007) | -0.000  (-0.005,  0.004) | 0.600 (0.582,  0.618) | -0.000  (-0.002,  0.002) |
| **Specific cancer sites** | | | | | | | | |
| Colorectal | 0.638 (0.607,  0.670) | 0.630 (0.600,  0.662) | -0.007   (-0.025,  0.010) | 0.629  (0.599,  0.661) | -0.008   (-0.028,  0.011) | -0.001  (-0.014,  0.011) | 0.6381 (0.6078,  0.67) | 0.001  (-0.007,  0.009) |
| Pancreas | 0.612 (0.569,  0.658) | 0.623 (0.580,  0.668) | 0.010  (-0.022,  0.043) | 0.634  (0.591,  0.679) | 0.021   (-0.017,  0.060) | 0.011  (-0.008,  0.030) | 0.633 (0.591,  0.678) | 0.009  (-0.032,  0.050) |
| Kidney | 0.644 (0.596,  0.696) | 0.643 (0.594  0.695) | -0.001   (-0.005,  0.003) | 0.643  (0.595,  0.695) | -0.000   (-0.005,  0.005) | 0.001   (-0.003,  0.004) | 0.643 (0.595,  0.695) | -0.000   (-0.001,  0.001) |
| Bladder | 0.654 (0.606,  0.707) | 0.657 (0.609,  0.709) | 0.003  (-0.024,  0.030) | 0.653  (0.606,  0.703) | -0.002   (-0.028,  0.025) | -0.005  (-0.019,  0.010) | 0.653 (0.605,  0.705) | 0.003  (-0.025,  0.030) |
| Lung | 0.720 (0.699,  0.741) | 0.729 (0.708,  0.751) | 0.009  (-0.003,  0.021) | 0.738  (0.716,  0.760) | 0.018  (0.001  0.035) | 0.009  (0.001,  0.017) | 0.748 (0.727,  0.770) | 0.015   (-0.000,  0.030) |
| Prostate | 0.589 (0.574,  0.604) | 0.589 (0.574,  0.604) | 0.000  (-0.001,  0.001) | 0.589  (0.575,  0.604) | 0.000  (-0.002,  0.003) | 0.000  (-0.002,  0.003) | 0.588 (0.574,  0.603) | 0.000  (-0.001  ,0.001) |
| Metastatic prostate | 0.573 (0.525,  0.626) | 0.584 (0.534,  0.640) | 0.011  (-0.034,  0.056) | 0.589  (0.535,  0.647) | 0.015  (-0.046,  0.076) | 0.004  (-0.030,  0.039) | 0.542 (0.492,  0.598) | -0.031  (-0.131,  0.068) |
| **Women** | | | | | | | | |
| **All cancers** | 0.576  (0.565,  0.587) | 0.578 (0.567,  0.589) | 0.002  (-0.004,  0.008) | 0.577  (0.567,  0.588) | 0.001  (-0.006,  0.008) | -0.000   (-0.009,  0.008) | 0.577 (0.567,  0.587) | 0.002  (-0.006,  0.009) |
| **OBR-cancers** | 0.578 (0.565,  0.590) | 0.575 (0.562,  0.587) | -0.003   (-0.014,  0.008) | 0.568  (0.556,  0.581) | -0.009  (-0.020,  0.002) | -0.006  (-0.015,  0.002) | 0.577 (0.564,  0.589) | 0.001  (-0.007,  0.008) |
| **NOBR-cancers** | 0.628 (0.612,  0.645) | 0.629 (0.613,  0.645) | 0.001  (-0.002,  0.003) | 0.629  (0.613,  0.646) | 0.001  (-0.002,  0.004) | 0.001  (-0.001,  0.002) | 0.630 (0.614,  0.647) | 0.002  (-0.002,  0.006) |
| **NOBR-cancers excluding lung** | 0.578 (0.560,  0.597) | 0.583 (0.565,  0.601) | 0.004  (-0.002,  0.011) | 0.590  (0.571,  0.607) | 0.011  (-0.000,  0.021) | 0.006  (0.000,  0.012) | 0.592 (0.574,  0.611) | 0.005  (-0.005,  0.015) |
| **Specific cancer sites** | | | | | | | | |
| Colorectal | 0.613 (0.583,  0.645) | 0.586 (0.556,  0.617) | -0.028  (-0.052,  -0.004) | 0.582  (0.552,  0.613) | -0.031  (-0.065,  0.002) | -0.004  (-0.018,  0.011) | 0.627 (0.597,  0.658) | 0.011  (-0.020,  0.042) |
| Pancreas | 0.624 (0.566,  0.688) | 0.627 (0.569,  0.690) | 0.003  (-0.016,  0.021) | 0.620  (0.564,  0.681) | -0.004   (-0.033,  0.025) | -0.007  (-0.038,  0.025) | 0.637 (0.578,  0.702) | 0.002  (-0.012,  0.016) |
| Kidney | 0.650  (0.593,  0.712) | 0.659 (0.600,  0.725) | 0.010   (-0.021,  0.040) | 0.652  (0.593,  0.716) | 0.002  (-0.014,  0.018) | -0.008  (-0.035,  0.020) | 0.653 (0.594,  0.717) | 0.010   (-0.023,  0.046) |
| Lung | 0.762 (0.734,  0.790) | 0.762 (0.735,  0.791) | 0.000  (-0.001,  0.002) | 0.762  (0.734,  0.791) | 0.000  (-0.001,  0.002) | -0.000   (-0.002,  0.002) | 0.762 (0.735,  0.791) | 0.001  (-0.001,  0.002) |
| Endometrial | 0.687 (0.638,  0.739) | 0.710 (0.663,  0.759) | 0.023   (-0.008,  0.054) | 0.696  (0.651,  0.745) | 0.010   (-0.022,  0.041) | -0.013   (-0.037,  0.010) | 0.699 (0.653,  0.748) | 0.021  (-0.004,  0.046) |
| Ovarian | 0.625 (0.571,  0.686) | 0.629 (0.573,  0.691) | 0.004   (-0.017,  0.024) | 0.625  (0.570,  0.685) | -0.000   (-0.012,  0.011) | -0.004   (-0.024,  0.016) | 0.626 (0.568,  0.689) | 0.007   (-0.034,  0.048) |
| Post-menopausal breast cancer | 0.576 (0.558,0.5945) | 0.582 (0.565,  0.601) | 0.006  (-0.006,  0.018) | 0.583  (0.565,  0.601) | 0.006  (-0.005,  0.019) | 0.000  (-0.008,  0.009) | 0.582 (0.565,  0.601) | 0.006   (-0.010,  0.021) |

5

| 1. **Harrell’s C-statistic (95% CI)** | | | | | | | |
| --- | --- | --- | --- | --- | --- | --- | --- |
| **Characteristic** | **WC -years with single BMI** | **Difference in c-statistic between WC-years with**  **single BMI vs WC-years** | **Single WC with single BMI** | **Difference in c-statistic between**  **single BMI with single WC and WC-years** | **Cumulative degree of excess WC** | **Cumulative duration of excess WC** | **Difference in c-statistic between**  **cumulative WC duration and WC degree** |
| **All cancers** | 0.577 (0.567,  0.587) | 0.001  (-0.004,  0.006) | 0.584  (0.573,  0.595) | -0.003  (-0.008,  0.002) | 0.586 (0.576,  0.596) | 0.583  (0.574,  0.593) | -0.003  (-0.011,  0.006) |
| **OBR-cancers** | 0.582 (0.562,  0.602) | 0.000  (-0.002,  0.002) | 0.578 (0.559,  0.597) | -0.002  (-0.013,  0.010) | 0.582 (0.562,  0.602) | 0.574  (0.554,  0.593) | -0.008   (-0.021,  0.005) |
| **NOBR-cancers** | 0.595 (0.584,  0.605) | 0.001   (-0.004,  0.006) | 0.593 (0.582,  0.604) | 0.001   (-0.007,  0.008) | 0.593 (0.582,  0.604) | 0.592  (0.580,  0.604) | -0.001  (-0.011,  0.009) |
| **NOBR-cancers excluding lung and prostate** | 0.600 (0.582,  0.618) | -0.001   (-0.003,  0.002) | 0.600 (0.582,  0.618) | -0.000  (-0.008,  0.007) | 0.600 (0.582,  0.618) | 0.599  (0.581,  0.617) | -0.001  (-0.010,  0.007) |
|  | | | | | | | |
| Colorectal | 0.638 (0.608,  0.670) | 0.001   (-0.009,  0.100) | 0.629 (0.599,  0.661) | -0.007  (-0.025,  0.011) | 0.638 (0.607,  0.670) | 0.629  (0.598,  0.661) | -0.009  (-0.028,  0.010) |
| Pancreas | 0.633 (0.590,  0.680) | 0.021  (-0.029,  0.071) | 0.634 (0.590,  0.680) | 0.021   (-0.020,  0.061) | 0.615 (0.571,  0.662) | 0.643  (0.601,  0.689) | 0.029  (-0.021,  0.078) |
| Kidney | 0.643 (0.595,  0.695) | -0.001   (-0.004,  0.003) | 0.643 (0.595,  0.695) | 0.000   (-0.006,  0.006) | 0.644 (0.595,  0.696) | 0.640  (0.592,  0.693) | -0.003  (-0.014,  0.007) |
| Bladder | 0.653 (0.606,  0.704) | -0.001  (-0.025,  0.023) | 0.653 (0.605,  0.704) | 0.002   (-0.025,  0.029) | 0.655 (0.606,  0.707) | 0.707  (0.657,  0.760) | 0.052  (0.003,  0.101) |
| Lung | 0.748 (0.726,  0.771) | 0.028  (0.008,  0.048) | 0.738 (0.716,  0.760) | 0.023  (0.005,  0.041) | 0.720 (0.699,  0.741) | 0.722  (0.702,  0.744) | 0.002  (-0.001^,^  0.006) |
| Prostate | 0.588 (0.574,  0.603) | -0.001  (-0.003,  0.002) | 0.589 (0.575,  0.604) | -0.001  (-0.000,  0.002) | 0.590 (0.574,  0.604) | 0.588  (0.574,  0.603) | -0.001  (-0.004,  0.003) |
| Metastatic prostate | 0.542 (0.492,  0.597) | -0.031   (-0.117,  0.054) | 0.589 (0.536,  0.647) | 0.012  (-0.066,  0.091) | 0.573 (0.525,  0.626) | 0.551  (0.510,  0.595) | -0.023  (-0.100,  0.054) |
| **Women** | | | | | | | |
| **All cancers** | 0.577 (0.566,  0.588) | 0.002  (-0.003,  0.007) | 0.577 (0.567,  0.587) | 0.002  (-0.004,  0.008) | 0.576 (0.565,  0.587) | 0.577  (0.566,  0.588) | 0.001  (-0.009,  0.010) |
| **OBR-cancers** | 0.577 (0.564,  0.589) | -0.001  (-0.006,  0.004) | 0.568 (0.556,  0.581) | -0.003  (-0.014,  0.007) | 0.578 (0.565,  0.590) | 0.576  (0.563,  0.588) | -0.002  (-0.015,  0.011) |
| **NOBR-cancers** | 0.630 (0.614,  0.646) | 0.002  (-0.003,  0.007) | 0.629 (0.613,  0.646) | 0.001  (-0.002,  0.005) | 0.630 (0.612,  0.645) | 0.629  (0.613,  0.646) | 0.001  (-0.002,  0.003) |
| **NOBR-cancers excluding lung** | 0.592 (0.574,  0.610) | 0.014  (-0.002,  0.029) | 0.590 (0.571,  0.607) | 0.012  (-0.000,  0.025) | 0.579 (0.560,  0.597) | 0.580  (0.562,  0.598) | 0.001  (-0.004,  0.006) |
| **Specific cancer sites** | | | | | | | |
| Colorectal | 0.627 (0.597,  0.658) | 0.014  (-0.025,  0.052) | 0.582 (0.552,  0.613) | -0.031  (-0.066,  0.003) | 0.612 (0.582,  0.644) | 0.614  (0.583,  0.646) | 0.001  (-0.026,  0.029) |
| Pancreas | 0.637 (0.583,  0.696) | 0.013  (-0.022,  0.048) | 0.620 (0.570,  0.674) | 0.028  (-0.031,  0.087) | 0.624 (0.566,  0.688) | 0.634  (0.576,  0.698) | 0.010  (-0.032,  0.056) |
| Kidney | 0.653 (0.594,  0.717) | 0.003  (-0.012,  0.018) | 0.652 (0.593,  0.717) | 0.009  (-0.017,  0.035) | 0.651 (0.594,  0.713) | 0.656  (0.593,  0.724) | 0.005  (-0.052,  0.062) |
| Lung | 0.762 (0.735,  0.791) | 0.001  (-0.001,  0.003) | 0.762 (0.734,  0.791) | 0.003  (-0.002,  0.008) | 0.762 (0.734,  0.790) | 0.763  (0.736,  0.792) | 0.002  (-0.001,  0.004) |
| Endometrial | 0.699 (0.652,  0.748) | 0.012  (-0.009,  0.033) | 0.696 (0.651,  0.745) | 0.019  (-0.011,  0.049) | 0.689 (0.641,  0.740) | 0.674  (0.632,  0.719) | -0.015  (-0.047,  0.017) |
| Ovarian | 0.626 (0.571,  0.686) | 0.000  (-0.005,  0.005) | 0.625 (0.568,  0.689) | 0.001  (-0.042,  0.044) | 0.626 (0.571,  0.686) | 0.648  (0.596,  0.706) | 0.023  (-0.009,  0.055) |
| Post-menopausal breast cancer | 0.582 (0.565,  0.601) | 0.006  (-0.008,  0.020) | 0.583 (0.565,  0.601) | 0.007  (-0.005,  0.019) | 0.576 (0.559,  0.595) | 0.584  (0.566,  0.603) | 0.008  (-0.006,  0.022) |
| **Key: Green – significant difference in C-statistic.**  **Abbreviations**: SE, standard error; OBR, obesity-related; NOBR, non-obesity related; BMI, body mass index; CI, confidence interval; MV, multivariable-adjusted, WC, waist circumference. | | | | | | | |

**Analysis with White participants only**

**Table S29: Summary of the exposure metrics in White participants only.**

| **Characteristic** | **Men** |
| --- | --- |
| **Baseline BMI, kg/m^2^** | 28.50 (4.40) |
| **Baseline WC, cm** | 104.00 (11.00) |
| **Follow up years** | 13.90 (6.00) |
| **Total cumulative waist circumference years** | 33.00 (57.00) |
| **Total cumulative waist circumference degree** | 16.00 (27.00) |
| **Total cumulative waist circumference duration** | 4.38 (4.04) |
|  | **Women** |
| **Baseline BMI, kg/m^2^** | 28.20 (5.90) |
| **Baseline WC, cm** | 100.00 (16.00) |
| **Follow up years** | 15.80 (5.20) |
| **Total cumulative waist circumference years** | 87.00 (106.00) |
| **Total cumulative waist circumference degree** | 42.00 (48.00) |
| **Total cumulative waist circumference duration** | 6.31 (3.55) |
| Mean (SD)  **Abbreviations**: N = number of participants; SD, standard deviation; BMI, body mass index, WC, waist circumference. | |

**Table S30: Incidence of cancer (events/1000 Person-Years) according to cumulative waist circumference years stratified by biological sex, smoking, HRT in White participants only.**

| **Men** | | | | | | | | | | | | | | |
| --- | --- | --- | --- | --- | --- | --- | --- | --- | --- | --- | --- | --- | --- | --- |
|  | **0 cm-years** | | | **≤100 cm-years** | | | **>100 cm-years** | | | **Baseline cohort** | | | |  |
|  | **N** | **PYFU** | **IR (95% CI)** | **N** | **PYFU** | **IR (95% CI)** | **N** | **PYFU** | **IR (95% CI)** | **N** | **PYFU** | **IR (95% CI)** |  |  |
| **Whole sample** | 760 | 144018.80 | 5.28 (4.90,5.66) | 487 | 91142.26 | 5.34 (4.86,5.82) | 267 | 41240.44 | 6.47 (5.69,7.26) | 1514 | 276401.50 | 5.48 (5.20,5.76) |  |  |
| **Smoking** | | | | | | | | | | | | | | |
| **Ever** | 555 | 98286.49 | 5.65 (5.17,6.12) | 374 | 66675.37 | 5.61 (5.03,6.19) | 195 | 30308.06 | 6.42 (5.50,7.34) | 1124 | 195269.90 | 5.76 (5.42,6.09) |  |  |
| **Never** | 205 | 45732.34 | 4.48 (3.85,5.10) | 113 | 24466.90 | 4.61 (3.74,5.49) | 72 | 10932.38 | 6.62 (5.05,8.20) | 390 | 81131.62 | 4.81 (4.32,5.29) |  |  |
| **Women** | | | | | | | | | | | | | | |
| **Whole sample** | 203 | 82058.92 | 2.47 (2.13,2.82) | 297 | 103911.00 | 2.86 (2.53,3.19) | 429 | 115263.80 | 3.72 (3.37,4.08) | 929 | 301233.70 | 3.08 (2.88,3.28) |  |  |
| **Smoking** | | | | | | | | | | | | | | |
| **Ever** | 128 | 41312.29 | 3.09 (2.54,3.64) | 168 | 50679.78 | 3.31 (2.8,3.83) | 211 | 50973.80 | 4.14 (3.57,4.71) | 507 | 142965.90 | 3.54 (3.23,3.86) |  |  |
| **Never** | 75 | 40746.63 | 1.85 (1.42,2.28) | 129 | 53231.21 | 2.42 (2.00,2.85) | 218 | 64290.00 | 3.39 (2.93,3.85) | 422 | 158267.80 | 2.67 (2.41,2.93) |  |  |
| **HRT** | | | | | | | | | | | | | | |
| **Ever** | 129 | 50618.47 | 2.55 (2.10,3.00) | 175.5 | 59146.59 | 2.97 (2.52,3.42) | 199 | 55835.52 | 3.57 (3.06,4.07) | 504 | 165600.60 | 3.04 (2.77,3.31) |  |  |
| **Never** | 74 | 31440.45 | 2.36 (1.80,2.91) | 121.5 | 44764.4 | 2.71 (2.22,3.21) | 230 | 59428.28 | 3.87 (3.36,4.38) | 426 | 135633.10 | 3.14 (2.84,3.44) |  |  |
| **Abbreviations**: N, number of cancer events; PYFR, person-years of follow-up; IR, incidence rate of all cancers , WC, waist circumference. | | | | | | | | | | | | | | |

**Table S31: Hazard ratio of specific cancers related to waist-circumference years in White participants only.**

| **Outcomes** | **Cancers** | **Waist circumference-years**  **(per SD)** | | **Baseline WC**  **(per SD)** | | **Baseline BMI**  **(per SD)** | |
| --- | --- | --- | --- | --- | --- | --- | --- |
|  |  | **Age-adjusted HR**  **(95% CI)** | **MV-adjusted HR**  **(95% CI)** | **Age-adjusted HR**  **(95% CI)** | **MV-adjusted HR**  **(95% CI)** | **Age-adjusted HR**  **(95% CI)** | **MV-adjusted HR**  **(95% CI)** |
|  |  | **Men** | | | | | |
| All Cancers | 1,213 | 1.08 (1.02,1.13) | 1.07 (1.02,1.13) | 1.06 (1.00,1.12) | 1.05 (0.99,1.11) | 1.03 (0.97,1.09) | 1.03 (0.97,1.08) |
| OBR-cancers | 248 | 1.14 (1.03,1.26) | 1.14 (1.03,1.26) | 1.12 (0.99,1.27) | 1.13 (1.00,1.27) | 1.12 (0.99,1.26) | 1.12 (0.99,1.27) |
| NOBR-cancers | 965 | 1.06 (1.00,1.12) | 1.06 (1.00,1.12) | 1.04 (0.98,1.11) | 1.03 (0.97,1.10) | 1.01 (0.94,1.07) | 1.00 (0.94,1.07) |
| NOBR-cancers excluding lung and prostate | 373 | 1.14 (1.05,1.24) | 1.14 (1.05,1.24) | 1.13 (1.02,1.25) | 1.12 (1.01,1.24) | 1.12 (1.01,1.24) | 1.11 (1.01,1.23) |
| **Specific cancer sites** | | | | | | | |
| Colorectal | 98 | 1.22 (1.05,1.42) | 1.23 (1.06,1.43) | 1.25 (1.06,1.48) | 1.27 (1.07,1.49) | 1.27 (1.08,1.49) | 1.26 (1.08,1.48) |
| Pancreas | 33 | 1.07 (0.80,1.43) | 1.08 (0.81,1.44) | 1.11 (0.83,1.48) | 1.13 (0.85,1.51) | 1.16 (0.88,1.54) | 1.16 (0.88,1.53) |
| Lung | 178 | 0.99 (0.87,1.13) | 0.96 (0.84,1.1) | 0.89 (0.78,1.03) | 0.87 (0.75,1.00) | 0.77 (0.66,0.89) | 0.77 (0.66,0.89) |
| Prostate | 414 | 0.98 (0.90,1.06) | 1.00 (0.92,1.08) | 0.97 (0.90,1.06) | 1.00 (0.93,1.09) | 1.01 (0.93,1.10) | 1.01 (0.93,1.10) |

| **Women** | | | | | | | |
| --- | --- | --- | --- | --- | --- | --- | --- |
| All Cancers | 929 | 1.13 (1.06,1.19) | 1.14 (1.08,1.21) | 1.15 (1.08,1.22) | 1.16 (1.09,1.24) | 1.14 (1.07,1.21) | 1.16 (1.09,1.24) |
| OBR-cancers | 539 | 1.20 (1.12,1.29) | 1.22 (1.14,1.31) | 1.22 (1.13,1.33) | 1.25 (1.15,1.35) | 1.2 (1.11,1.3) | 1.23 (1.13,1.33) |
| NOBR-cancers | 390 | 1.02 (0.93,1.13) | 1.03 (0.93,1.13) | 1.05 (0.95,1.16) | 1.05 (0.95,1.17) | 1.05 (0.95,1.16) | 1.07 (0.97,1.18) |
| NOBR-cancers excluding lung | 266 | 1.03 (0.92,1.16) | 1.04 (0.92,1.17) | 1.06 (0.94,1.20) | 1.06 (0.94,1.20) | 1.10 (0.98,1.24) | 1.11 (0.99,1.25) |
| **Specific cancer sites** | | | | |  |  |  |
| Colorectal | 85 | 1.23 (1.03,1.46) | 1.25 (1.05,1.49) | 1.09 (0.88,1.34) | 1.10 (0.89,1.36) | 1.05 (0.85,1.30) | 1.07 (0.87,1.33) |
| Pancreas | 22 | 1.26 (0.99,1.76) | 1.24 (0.89,1.75) | 1.31 (0.88,1.94) | 1.30 (0.87,1.94) | 1.23 (0.83,1.82) | 1.21 (0.82,1.80) |
| Kidney | 26 | 1.31 (1.00,1.73) | 1.37 (1.04,1.81) | 1.43 (1.00,2.04) | 1.51 (1.05,2.17) | 1.39 (0.99,1.95) | 1.48 (1.05,2.09) |
| Lung | 124 | 1.00 (0.84,1.19) | 1.01 (0.85,1.20) | 1.02 (0.85,1.22) | 1.03 (0.86,1.23) | 0.95 (0.79,1.14) | 0.98 (0.81,1.17) |
| Endometrial | 62 | 1.59 (1.35,1.88) | 1.57 (1.33,1.86) | 1.85 (1.49,2.29) | 1.83 (1.48,2.28) | 1.74 (1.43,2.11) | 1.72 (1.41,2.11) |
| Ovarian | 36 | 1.05 (0.76,1.45) | 1.08 (0.78,1.50) | 1.09 (0.79,1.50) | 1.13 (0.81,1.56) | 0.97 (0.7,1.35) | 1.00 (0.71,1.41) |
| Post-menopausal breast cancer | 267 | 1.12 (1.00,1.24) | 1.14 (1.02,1.27) | 1.17(1.04,1.32) | 1.20 (1.07,1.35) | 1.18 (1.06,1.32) | 1.21 (1.08,1.36) |
| * Multivariable adjustment for baseline age, race, alcohol, smoking and HRT (in women).  **Abbreviations**: OBR, obesity-related; NOBR, non-obesity related; CI, confidence interval; HR, hazard ratio; BMI, body mass index; MV, multivariable, WC, waist circumference. | | | | | | | |

**Table S32: Hazard ratio of specific cancers related to waist circumference years in White participants only.**

| **Outcomes** | **Cancers** | **Waist circumference-years**  **(per 100 cm-years)** | | **Baseline WC**  **(per 5 cm)** | | **Baseline BMI**  **(per 5 kg/m2)** | |
| --- | --- | --- | --- | --- | --- | --- | --- |
|  |  | **Age-adjusted HR (95% CI)** | **MV-adjusted HR**  **(95% CI)** | **Age-adjusted HR**  **(95% CI)** | **MV-adjusted HR**  **(95% CI)** | **Age-adjusted HR**  **(95% CI)** | **MV-adjusted HR**  **(95% CI)** |
|  |  | **Men** | | | | | |
| All Cancers | 1,213 | 1.09 (1.03,1.15) | 1.08 (1.02,1.14) | 1.03 (1.00,1.05) | 1.02 (1.00,1.05) | 1.03 (0.97,1.10) | 1.03 (0.96,1.10) |
| OBR-cancers | 248 | 1.15 (1.03,1.29) | 1.15 (1.03,1.29) | 1.05 (1.00,1.11) | 1.06 (1.00,1.12) | 1.14 (0.99,1.32) | 1.14 (0.99,1.32) |
| NOBR-cancers | 965 | 1.07 (1.00,1.14) | 1.06 (1.00,1.13) | 1.02 (0.99,1.05) | 1.01 (0.98,1.04) | 1.01 (0.93,1.09) | 1.00 (0.93,1.08) |
| NOBR-cancers excluding lung and prostate | 373 | 1.16 (1.06,1.27) | 1.15 (1.05,1.27) | 1.06 (1.01,1.10) | 1.05 (1.00,1.10) | 1.14 (1.01,1.28) | 1.13 (1.01,1.27) |
| **Specific cancer sites** | | | | | | | |
| Colorectal | 98 | 1.20 (1.02,1.41) | 1.20 (1.02,1.41) | 1.07 (0.98,1.17) | 1.07 (0.98,1.17) | 1.18 (0.95,1.48) | 1.18 (0.94,1.48) |
| Pancreas | 33 | 1.15 (0.82,1.62) | 1.15 (0.82,1.61) | 1.11 (0.96,1.28) | 1.10 (0.95,1.28) | 1.37 (0.96,1.97) | 1.37 (0.96,1.96) |
| Lung | 178 | 1.01 (0.87,1.19) | 0.99 (0.84,1.17) | 0.95 (0.88,1.01) | 0.93 (0.86,0.99) | 0.72 (0.59,0.88) | 0.71 (0.59,0.86) |
| Prostate | 414 | 1.00 (0.90,1.12) | 1.00 (0.90,1.12) | 1.02 (0.97,1.06) | 1.02 (0.97,1.06) | 1.02 (0.91,1.14) | 1.02 (0.91,1.14) |

| **Women** | | | | | | | |
| --- | --- | --- | --- | --- | --- | --- | --- |
| All Cancers | 929 | 1.07 (1.03,1.10) | 1.07 (1.04,1.11) | 1.05 (1.02,1.07) | 1.05 (1.03,1.07) | 1.12 (1.06,1.18) | 1.14 (1.08,1.20) |
| OBR-cancers | 539 | 1.10 (1.06,1.15) | 1.11 (1.07,1.16) | 1.07 (1.04,1.09) | 1.07 (1.05,1.10) | 1.17 (1.10,1.26) | 1.19 (1.11,1.28) |
| NOBR-cancers | 390 | 1.01 (0.96,1.07) | 1.02 (0.96,1.07) | 1.02 (0.98,1.05) | 1.02 (0.98,1.05) | 1.04 (0.96,1.14) | 1.06 (0.97,1.15) |
| NOBR-cancers excluding lung | 266 | 1.02 (0.96,1.08) | 1.02 (0.96,1.09) | 1.02 (0.98,1.06) | 1.02 (0.98,1.06) | 1.09 (0.98,1.20) | 1.09 (0.99,1.21) |
| **Specific cancer sites** | | | | |  |  |  |
| Colorectal | 85 | 1.12 (1.02,1.23) | 1.13 (1.02,1.24) | 1.03 (0.96,1.10) | 1.03 (0.96,1.10) | 1.04 (0.87,1.25) | 1.06 (0.88,1.27) |
| Pancreas | 22 | 1.13 (0.94,1.36) | 1.13 (0.94,1.35) | 1.09 (0.96,1.24) | 1.09 (0.95,1.23) | 1.19 (0.85,1.67) | 1.18 (0.84,1.66) |
| Kidney | 26 | 1.16 (1.00,1.35) | 1.19 (1.02,1.38) | 1.12 (1.00,1.25) | 1.14 (1.02,1.28) | 1.33 (0.99,1.77) | 1.40 (1.04,1.88) |
| Lung | 124 | 1.00 (0.91,1.10) | 1.00 (0.91,1.10) | 1.01 (0.95,1.07) | 1.01 (0.95,1.07) | 0.95 (0.81,1.12) | 0.98 (0.84,1.15) |
| Endometrial | 62 | 1.29 (1.18,1.41) | 1.28 (1.17,1.40) | 1.22 (1.14,1.30) | 1.21 (1.13,1.30) | 1.61 (1.36,1.90) | 1.60 (1.35,1.90) |
| Ovarian | 36 | 1.02 (0.86,1.22) | 1.04 (0.87,1.25) | 1.03 (0.93,1.14) | 1.04 (0.94,1.15) | 0.97 (0.73,1.30) | 1.00 (0.75,1.34) |
| Post-menopausal breast cancer | 267 | 1.06 (1.00,1.13) | 1.07 (1.01,1.14) | 1.05 (1.01,1.09) | 1.06 (1.02,1.10) | 1.16 (1.05,1.27) | 1.18 (1.07,1.30) |
| * Multivariable adjustment for baseline age, race, alcohol, smoking and HRT (in women).  **Abbreviations**: OBR, obesity-related; NOBR, non-obesity related; CI, confidence interval; HR, hazard ratio; BMI, body mass index; MV, multivariable, WC, waist circumference. | | | | | | | |

**Table S33: Comparison of the waist circumference degree and duration per unit standard deviation in White participants only.**

| **Outcomes** |  | **Cumulative degree of excess WC (per SD)** | | **Cumulative duration of excess WC (per SD)** | |
| --- | --- | --- | --- | --- | --- |
|  |  | **Age-adjusted HR (95% CI)** | **MV-adjusted HR (95% CI)** | **Age-adjusted HR (95% CI)** | **MV-adjusted HR (95% CI)** |
|  | **Cancers** | **Men** | | | |
| All Cancers | 1,213 | 1.09 (1.00,1.18) | 1.09 (1.01,1.19) | 1.06 (0.97,1.15) | 1.05 (0.97,1.15) |
| OBR-cancers | 248 | 1.13 (0.96,1.34) | 1.13 (0.95,1.34) | 1.08 (0.90,1.31) | 1.09 (0.90,1.32) |
| NOBR-cancers | 965 | 1.07 (0.98,1.18) | 1.08 (0.99,1.19) | 1.05 (0.95,1.16) | 1.05 (0.95,1.15) |
| NOBR-cancers excluding lung and prostate | 373 | 1.13 (0.99,1.30) | 1.14 (0.99,1.32) | 1.18 (1.02,1.38) | 1.18 (1.01,1.37) |
| **Specific cancer sites** | | | |  |  |
| Colorectal | 98 | 1.19 (0.93,1.52) | 1.19 (0.93,1.52) | 1.11 (0.83,1.48) | 1.11 (0.83,1.48) |
| Pancreas | 33 | 0.89 (0.50,1.57) | 0.89 (0.51,1.58) | 1.24 (0.74,2.09) | 1.24 (0.74,2.09) |
| Lung | 178 | 1.12 (0.71,1.76) | 1.1 (0.70,1.71) | 0.97 (0.60,1.60) | 0.99 (0.60,1.63) |
| Prostate | 414 | 1.24 (1.02,1.50) | 1.29 (1.04,1.60) | 1.24 (1.01,1.53) | 1.2 (0.98,1.48) |

| **Women** | | | | | |
| --- | --- | --- | --- | --- | --- |
| All Cancers | 929 | 1.04 (0.93,1.16) | 1.05 (0.94,1.17) | 1.1 (0.99,1.22) | 1.11 (1.00,1.24) |
| OBR-cancers | 539 | 1.10 (0.95,1.27) | 1.11 (0.96,1.28) | 1.14 (0.99,1.31) | 1.14 (0.99,1.32) |
| NOBR-cancers | 390 | 0.95 (0.79,1.14) | 0.96 (0.8,1.15) | 1.06 (0.91,1.24) | 1.09 (0.93,1.27) |
| NOBR-cancers excluding lung | 266 | 0.96 (0.77,1.19) | 0.96 (0.77,1.19) | 1.00 (0.83,1.22) | 1.01 (0.84,1.23) |
| **Specific cancer sites** | | | |  |  |
| Colorectal | 85 | 1.58 (1.16,2.16) | 1.59 (1.16,2.17) | 1.65 (1.17,2.33) | 1.68 (1.19,2.37) |
| Pancreas | 22 | 1.11 (0.56,2.19) | 1.09 (0.55,2.16) | 1.15 (0.58,2.29) | 1.15 (0.58,2.29) |
| Kidney | 26 | 1.10 (0.62,1.96) | 1.13 (0.63,2.01) | 1.19 (0.65,2.19) | 1.23 (0.67,2.26) |
| Lung | 124 | 0.94 (0.67,1.30) | 0.95 (0.69,1.30) | 1.19 (0.9,1.56) | 1.25 (0.95,1.65) |
| Endometrial | 62 | 1.07 (0.72,1.58) | 1.06 (0.72,1.56) | 0.88 (0.56,1.37) | 0.88 (0.57,1.38) |
| Ovarian | 36 | 0.92 (0.49,1.73) | 0.93 (0.49,1.75) | 1.39 (0.80,2.39) | 1.40 (0.81,2.42) |
| Post-menopausal breast cancer | 267 | 0.94 (0.75,1.17) | 0.95 (0.76,1.19) | 1.10 (0.90,1.34) | 1.10 (0.9,1.34) |
| * Multivariable adjustment for baseline age, baseline WC, race, alcohol, smoking and HRT (in women).  * Degree of excess WC is the cumulative sum of the number of WC units ≥ 102 cm in men and ≥ 88 cm in women over the exposure period.  * Duration of excess WC is the cumulative sum of the duration of WC above the threshold over the exposure period.  **Abbreviations**: OBR, obesity-related; NOBR, non-obesity related; CI, confidence interval; HR, hazard ratio; BMI, body mass index; MV, multivariable, WC, waist circumference. | | | | | |

**Table S34: Comparison of the waist circumference degree and duration per 10 units and per 10 years respectively stratified by biological sex in White participants only.**

| **Outcome** | **Cumulative degree of excess WC**  **(per 10 cm)** | | | **Cumulative duration of excess WC**  **(per 10 years)** | | | |
| --- | --- | --- | --- | --- | --- | --- | --- |
|  | **Age-adjusted HR**  **(95% CI)** | **MV-adjusted HR**  **(95% CI)** | | **Age-adjusted HR**  **(95% CI)** | **MV-adjusted HR**  **(95% CI)** | | |
| **Men** | | | | | | | |
| **All Cancers** | 1.01 (1.00,1.02) | 1.01 (1.00,1.02) | | 1.08 (0.96,1.21) | 1.07 (0.96,1.20) | | |
| **OBR-cancers** | 1.01 (1.00,1.03) | 1.01 (1.00,1.03) | | 1.11 (0.87,1.43) | 1.12 (0.87,1.43) | | |
| **NOBR-cancers** | 1.01 (1.00,1.02) | 1.01 (1.00,1.02) | | 1.07 (0.94,1.21) | 1.06 (0.94,1.20) | | |
| **NOBR-cancers excluding lung and prostate** | 1.01 (1.00,1.03) | 1.01 (1.00,1.03) | | 1.25 (1.02,1.52) | 1.24 (1.02,1.51) | | |
| **Specific cancer sites** | | | | | | | |
| Colorectal | 1.02 (0.99,1.04) | 1.02 (0.99,1.04) | | 1.14 (0.78,1.66) | 1.14 (0.78,1.66) | | |
| Pancreas | 0.99 (0.93,1.05) | 0.99 (0.93,1.05) | | 1.33 (0.67,2.62) | 1.33 (0.67,2.61) | | |
| Lung | 1.02 (1.00,1.04) | 1.03 (1.00,1.05) | | 1.33 (1.01,1.75) | 1.27 (0.97,1.67) | | |
| Prostate | 0.99 (0.98,1.01) | 0.99 (0.98,1.01) | | 0.83 (0.68,1.02) | 0.84 (0.68,1.03) | | |
| **Women** | | | | | | | |
| **All Cancers** | 1.00 (1.00,1.01) | | 1.00 (1.00,1.01) | 1.12 (0.98,1.27) | | 1.14 (1.00,1.29) |  |
| **OBR-cancers** | 1.00 (1.00,1.01) | | 1.01 (1.00,1.01) | 1.16 (0.98,1.38) | | 1.18 (0.99,1.39) |  |
| **NOBR-cancers** | 1.00 (0.99,1.01) | | 1.00 (0.99,1.01) | 1.07 (0.89,1.30) | | 1.11 (0.91,1.34) |  |
| **NOBR-cancers excluding lung** | 1.00 (0.99,1.01) | | 1.00 (0.99,1.01) | 1.00 (0.80,1.26) | | 1.02 (0.81,1.28) |  |
| **Specific cancer sites** | | | | | | |  |
| Colorectal | 1.02 (1.01,1.04) | | 1.02 (1.01,1.04) | 1.82 (1.20,2.76) | | 1.86 (1.23,2.82) |  |
| Pancreas | 1.01 (0.97,1.04) | | 1.00 (0.97,1.04) | 1.19 (0.52,2.71) | | 1.19 (0.52,2.70) |  |
| Kidney | 1.00 (0.98,1.03) | | 1.01 (0.98,1.04) | 1.23 (0.59,2.57) | | 1.28 (0.61,2.66) |  |
| Lung | 1.00 (0.98,1.01) | | 1.00 (0.98,1.01) | 1.23 (0.88,1.71) | | 1.31 (0.94,1.83) |  |
| Endometrial | 1.00 (0.98,1.02) | | 1.00 (0.98,1.02) | 0.85 (0.50,1.46) | | 0.86 (0.50,1.47) |  |
| Ovarian | 1.00 (0.96,1.03) | | 1.00 (0.96,1.03) | 1.48 (0.77,2.85) | | 1.50 (0.78,2.89) |  |
| Post-menopausal breast cancer | 1.00 (0.99,1.01) | | 1.00 (0.99,1.01) | 1.12 (0.88,1.42) | | 1.12 (0.88,1.42) |  |
| * Multivariable adjustment for baseline age, baseline WC, race, alcohol, smoking and HRT (in women).  * Degree of excess WC is the cumulative sum of the number of WC units ≥ 102 cm in men and ≥ 88 cm in women over the exposure period.  * Duration of excess WC is the cumulative sum of the duration of WC above the threshold over the exposure period.  **Abbreviations**: OBR, obesity-related; NOBR, non-obesity related; CI, confidence interval; HR, hazard ratio; BMI, body mass index; MV, multivariable, WC, waist circumference. | | | | | | |  |

|  |  | |  | |  | | **AIC** | | | | |
| --- | --- | --- | --- | --- | --- | --- | --- | --- | --- | --- | --- |
| **Characteristic** | **MV-adjusted waist circumference-years** | **MV-adjusted single**  **WC** | | **MV-adjusted single BMI** | | **MV-adjusted waist circumference -years with single WC** | | **MV-adjusted waist circumference -years with single BMI** | **MV-adjusted single WC -years with single BMI** | **MV-adjusted cumulative degree of excess WC** | **MV-adjusted cumulative duration of excess WC** |
| **Men** | | | | | | | | | | | |
| **All cancers** | 19026.78 | 19031.08 | | 19032.99 | | 19026.20 | | 19028.40 | 19031.02 | 19026.97 | 19029.63 |
| **OBR-cancers** | 3890.35 | 3892.05 | | 3892.27 | | 3892.31 | | 3892.31 | 3893.98 | 3890.21 | 3891.58 |
| **NOBR-cancers** | 15148.93 | 15151.30 | | 15152.06 | | 15147.43 | | 15150.36 | 15150.25 | 15149.12 | 15150.49 |
| **NOBR-cancers excluding lung and prostate** | 5808.37 | 5811.66 | | 5811.91 | | 5810.37 | | 5810.37 | 5813.55 | 5808.41 | 5807.29 |
| **Specific cancer sites** | | | | | | | | | | | |
| Colorectal | 1534.50 | 1536.14 | | 1536.27 | | 1536.50 | | 1536.49 | 1538.10 | 1534.47 | 1535.84 |
| Pancreas | 520.39 | 519.26 | | 518.29 | | 519.68 | | 521.05 | 520.08 | 520.30 | 518.65 |
| Kidney | 732.69 | 732.65 | | 732.66 | | 734.55 | | 734.51 | 734.65 | 732.69 | 732.66 |
| Lung | 2720.58 | 2715.92 | | 2707.53 | | 2697.16 | | 2712.78 | 2704.10 | 2720.55 | 2720.24 |
| Prostate | 6567.15 | 6566.67 | | 6567.01 | | 6568.90 | | 6568.12 | 6568.33 | 6567.16 | 6566.87 |
| **Women** | | | | | | | | | | | |
| **All cancers** | 14996.03 | 14992.84 | | 14993.21 | | 14994.13 | | 14994.13 | 14993.99 | 14995.62 | 14991.52 |
| **OBR-cancers** | 8734.40 | 8733.38 | | 8736.17 | | 8734.35 | | 8734.35 | 8735.17 | 8733.97 | 8734.33 |
| **NOBR-cancers** | 6229.30 | 6228.59 | | 6228.00 | | 6229.50 | | 6229.50 | 6229.90 | 6229.27 | 6227.52 |
| **NOBR-cancers excluding lung** | 4294.03 | 4293.40 | | 4291.53 | | 4292.09 | | 4292.09 | 4291.97 | 4294.00 | 4293.68 |
| **Specific cancer sites** | | | | | | | | | | | |
| Colorectal | 1391.07 | 1395.48 | | 1395.91 | | 1389.43 | | 1389.43 | 1397.26 | 1391.20 | 1389.29 |
| Pancreas | 366.19 | 366.05 | | 366.68 | | 368.19 | | 368.19 | 367.84 | 366.19 | 366.17 |
| Kidney | 431.99 | 431.34 | | 431.60 | | 433.29 | | 433.29 | 433.24 | 431.88 | 431.99 |
| Lung | 1903.74 | 1903.64 | | 1903.67 | | 1905.47 | | 1905.47 | 1903.98 | 1903.73 | 1902.12 |
| Endometrial | 994.43 | 987.92 | | 990.30 | | 991.45 | | 991.45 | 989.70 | 993.88 | 1004.88 |
| Ovarian | 597.54 | 597.23 | | 597.75 | | 599.25 | | 599.25 | 596.89 | 597.52 | 595.92 |
| Post-menopausal breast cancer | 4336.58 | 4332.74 | | 4331.27 | | 4333.01 | | 4333.01 | 4333.25 | 4336.43 | 4333.56 |
| * Multivariable adjustment for baseline age, race, alcohol, smoking and HRT (in women).  **Abbreviations**: SE, standard error; OBR, obesity-related; NOBR, non-obesity related; BMI, body mass index; AIC, Akaike information criterion, WC, waist circumference. | | | | | | | | | | | |

**Table S35: Comparison of the metrics calculated using WC predicted from the subgroup with at least 3 WC measurements by Akaike information criterion in White participants only.**

| **Table S36a and S36b: Comparison of the metrics calculated using WC predicted from the subgroup**  **in White participants only by Harrell’s C-index.** | | | | | | | | |
| --- | --- | --- | --- | --- | --- | --- | --- | --- |
| 1. **Harrell’s C-statistic (95% CI)** | | | | | | | | |
| **Characteristic** | **WC-years** | **Single WC** | **Difference in c-statistic between single WC vs WC-years** | **Single**  **BMI** | **Difference in c-statistic between**  **single BMI vs WC-years** | **Difference in c-statistic between single BMI vs single WC** | **WC-years with single WC** | **Difference in c-statistic between WC-years with**  **single WC and WC-years** |
| **Men** | | | | | | | | |
| **All cancers** | 0.590  (0.580,  0.600) | 0.588 (0.578,  0.598) | -0.002   (-0.008,  0.004) | 0.587  (0.577,  0.598) | -0.003  (-0.007,  0.002) | -0.001   (-0.006,  0.005) | 0.591 (0.581,  0.601) | -0.001   (-0.004,  0.004) |
| **OBR-cancers** | 0.577 (0.558,  0.597) | 0.570 (0.551  ,0.592) | -0.006  (-0.020,  0.007) | 0.568  (0.548,  0.589) | -0.009   (-0.022,  0.005) | -0.002   (-0.011,  0.007) | 0.576 (0.556,  0.596) | -0.000  (-0.003,  0.002) |
| **NOBR-cancers** | 0.595 (0.584,  0.607) | 0.594 (0.583,  0.606) | -0.001   (-0.009,  0.008) | 0.594  (0.582,  0.606) | -0.001  (-0.009,  0.006) | -0.001   (-0.005,  0.004) | 0.594 (0.583,  0.606) | 0.002   (-0.006,  0.010) |
| **NOBR-cancers excluding lung and prostate** | 0.602 (0.584,  0.620) | 0.601 (0.583,  0.619) | -0.001  (-0.008,  0.006) | 0.600  (0.582,  0.619) | -0.001   (-0.009,  0.006) | -0.000   (-0.004,  0.004) | 0.602 (0.584,  0.620) | -0.000   (-0.000,  0.000) |
| **Specific cancer sites** | | | | | | | | |
| Colorectal | 0.647  (0.594,  0.704) | 0.645 (0.594,  0.702) | -0.010  (-0.023,  0.003) | 0.646  (0.595,  0.702) | -0.012  (-0.027,  0.003) | -0.002  (-0.010,  0.006) | 0.647 (0.595,  0.703) | -0.000   (-0.002,  0.001) |
| Kidney | 0.590  (0.580,  0.600) | 0.588 (0.578,  0.598) | -0.001  (-0.014,  0.011) | 0.587  (0.577,  0.598) | -0.001   (-0.012,  0.010) | 0.001  (-0.004,  0.006) | 0.5901  (0.581,  0.601) | -0.005   (-0.019,  0.009) |
| Pancreas | 0.660 (0.604,  0.720) | 0.655 (0.595,  0.720) | -0.005  (-0.048,  0.038) | 0.662  (0.606,  0.723) | 0.003  (-0.052,  0.057) | 0.007  (-0.022,  0.037) | 0.664 (0.604,  0.731) | 0.000  (-0.054,  0.054) |
| Lung | 0.725 (0.704,  0.748) | 0.732 (0.710  ,0.755) | 0.006  (-0.005,  0.018) | 0.742  (0.719,  0.765) | 0.016  (-0.001,  0.034) | 0.010 (0.002,  0.018) | 0.750 (0.727,  0.773) | 0.010  (-0.006,  0.0251) |
| Prostate | 0.587  (0.571,  0.603) | 0.587 (0.572,  0.603) | 0.001  (-0.003,  0.005) | 0.587  (0.571,  0.603) | 0.000  (-0.002,  0.003) | -0.001  (-0.003,  0.002) | 0.586 (0.570,  0.602) | -0.001  (-0.007,  0.005) |
| **Women** | | | | | | | | |
| **All cancers** | 0.580 (0.569,  0.591) | 0.580 (0.569,  0.591) | 0.001   (-0.007,  0.008) | 0.577  (0.566,  0.589) | -0.002   (-0.013,  0.009) | -0.003   (-0.014,  0.008) | 0.579 (0.568,  0.590) | 0.000   (-0.008,  0.009) |
| **OBR-cancers** | 0.591 (0.577,  0.605) | 0.585 (0.571,  0.599) | -0.006  (-0.018,  0.006) | 0.580  (0.566,  0.594) | -0.011   (-0.024,  0.002) | -0.005   (-0.014,  0.005) | 0.589 (0.576,  0.604) | -0.001   (-0.008,  0.007) |
| **NOBR-cancers** | 0.629 (0.612,  0.646) | 0.629 (0.612,  0.647) | 0.001   (-0.002,  0.003) | 0.629  (0.613,  0.647) | 0.001  (-0.003,  0.004) | 0.000   (-0.002,  0.002) | 0.629 (0.612,  0.647) | 0.001  (-0.003,  0.004) |
| **NOBR-cancers excluding lung** | 0.581 (0.562,  0.601) | 0.584 (0.565,  0.603) | 0.003  (-0.004,  0.009) | 0.590  (0.571,  0.610) | 0.009  (-0.003,  0.021) | 0.006   (-0.006,  0.014) | 0.590 (0.571,  0.610) | 0.003  (-0.006,  0.011) |
| **Specific cancer sites** | | | | | | | | |
| Colorectal | 0.654 (0.587,  0.729) | 0.648 (0.581,  0.722) | -0.029   (-0.060,  0.001) | 0.640  (0.572,  0.716) | -0.033   (-0.066,  -0.000) | -0.004   (-0.012,  0.004) | 0.653 (0.586,  0.728) | 0.008  (-0.023,  0.038) |
| Pancreas | 0.703 (0.643,  0.768) | 0.700 (0.632,  0.774) | -0.006  (-0.031,  0.018) | 0.689  (0.626,  0.759) | -0.014   (-0.042,  0.015) | -0.007   (-0.034,  0.020) | 0.695 (0.629,  0.766) | -0.003   (-0.022,  0.016) |
| Kidney | 0.764 (0.735,  0.793) | 0.764 (0.735,  0.794) | -0.003   (-0.042,  0.036) | 0.763 (0.734,  0.793) | -0.013   (-0.039,  0.012) | -0.010   (-0.045,  0.024) | 0.764 (0.735,  0.793) | -0.002   (-0.033,  0.028) |
| Lung | 0.694 (0.645,  0.746) | 0.713(  0.665,  0.764) | 0.001   (-0.001,  0.002) | 0.700  (0.653,  0.750) | -0.000  (-0.002,  0.002) | -0.001   (-0.004,  0.002) | 0.704 (0.657,  0.755) | 0.001   (-0.001,  0.004) |
| Endometrial | 0.649 (0.594,  0.709) | 0.647 (0.590,  0.711) | 0.019  (-0.015,  0.053) | 0.646 (0.591,  0.706) | 0.006  (-0.032,  0.044) | -0.013  (-0.041,  0.0148) | 0.651 (0.591,  0.717) | 0.019  (-0.013,  0.051) |
| Ovarian | 0.587 (0.567,  0.607) | 0.595 (0.575,  0.615) | -0.002   (-0.019,  0.015) | 0.5967 (0.577,  0.617) | -0.004   (-0.028,  0.021) | -0.002  (-0.031,  0.0275) | 0.597 (0.577,  0.618) | -0.001   (-0.029,  0.027) |
| Post-menopausal breast cancer | 0.580 (0.569,  0.591) | 0.580 (0.569,  0.591) | 0.008   (-0.004,  0.020) | 0.577 (0.566,  0.589) | 0.010   (-0.003,  0.024) | 0.002   (-0.007,  0.011) | 0.579 (0.568,  0.590) | 0.008   (-0.006,  0.022) |

| 1. **Harrell’s C-statistic (95% CI)** | | | | | | | |
| --- | --- | --- | --- | --- | --- | --- | --- |
| **Characteristic** | **WC -years with single BMI** | **Difference in c-statistic between WC-years with**  **single BMI vs WC-years** | **Single WC with single BMI** | **Difference in c-statistic between**  **single BMI with single WC and WC-years** | **Cumulative degree of excess WC** | **Cumulative duration of excess WC** | **Difference in c-statistic between**  **cumulative WC duration and WC degree** |
| **All cancers** | 0.591 (0.581,  0.601) | 0.000   (-0.004,  0.004) | 0.588 (0.578,  0.599) | -0.002  (-0.007,  0.003) | 0.588 (0.578,  0.598) | 0.587  (0.577,  0.597) | -0.001  (-0.006,  0.005) |
| **OBR-cancers** | 0.576 (0.556,  0.596) | -0.001  (-0.004,  0.001) | 0.568 (0.549,  0.589) | -0.008  (-0.021,  0.006) | 0.577  (0.557,  0.597) | 0.564  (0.545,  0.585) | -0.012  (-0.026,  0.002) |
| **NOBR-cancers** | 0.594 (0.583,  0.606) | 0.004  (-0.004,  0.011) | 0.591 (0.580,  0.602) | 0.001  (-0.007,  0.010) | 0.596  (0.584,  0.608) | 0.595  (0.583,  0.607) | -0.001   (-0.008,  0.006) |
| **NOBR-cancers excluding lung and prostate** | 0.602 (0.584,  0.620) | 0.000  (-0.000,  0.000) | 0.600 (0.582,  0.619) | -0.001  (-0.008,  0.006) | 0.602 (0.584,  0.620) | 0.601  (0.583,  0.620) | -0.001  (-0.009,  0.008) |
| **Specific cancer sites** | | | | | | | |
| Colorectal | 0.647 (0.595,  0.703) | 0.000   (-0.000,  0.002) | 0.644 (0.594,  0.703) | -0.010   (-0.024,  0.003) | 0.647 (0.595,  0.704) | 0.645  (0.593,  0.702) | -0.008  (-0.023,  0.007) |
| Kidney | 0.591 (0.581,  0.601) | -0.000   (-0.013,  0.013) | 0.588 (0.578,  0.599) | -0.002   (-0.012,  0.011) | 0.588 (0.578,  0.598) | 0.587  (0.577,  0.597) | -0.002   (-0.015,  0.011) |
| Pancreas | 0.664 (0.609,  0.725) | 0.005  (-0.070,  0.077) | 0.662 (0.608,  0.720) | 0.005  (-0.058,  0.0670) | 0.661 (0.605,  0.721) | 0.658  (0.599,  0.722) | -0.003  (-0.039,  0.033) |
| Lung | 0.750 (0.727,  0.773) | 0.025  (0.004,  0.045) | 0.742 (0.719,  0.765) | 0.023  (0.004,  0.041) | 0.726  (0.704,  0.748) | 0.726  (0.705,  0.748) | 0.001  (-0.002,  0.003) |
| Prostate | 0.586 (0.570,  0.602) | -0.001  (-0.004,  0.003) | 0.587 (0.571,  0.603) | 0.001  (-0.004,  0.006) | 0.587  (0.571,  0.603) | 0.587  (0.571,  0.603) | 0.000  (-0.004,  0.004) |
| **Women** | | | | | | | |
| **All cancers** | 0.575 (0.565,  0.586) | 0.000  (-0.008,  0.008) | 0.580 (0.569,  0.591) | -0.002  (-0.009,  0.005) | 0.580  (0.569,  0.592) | 0.575  (0.565,  0.586) | 0.000   (-0.012,  0.012) |
| **OBR-cancers** | 0.580 (0.566,  0.594) | -0.002   (-0.008,  0.005) | 0.591 (0.578,  0.606) | -0.006   (-0.018,  0.005) | 0.582  (0.568,  0.596) | 0.580  (0.566,  0.594) | -0.009  (-0.024,  0.005) |
| **NOBR-cancers** | 0.629 (0.613,  0.647) | 0.001  (-0.004,  0.005) | 0.629 (0.612,  0.646) | 0.000  (-0.003,  0.004) | 0.631 (0.614,  0.648) | 0.629  (0.613,  0.647) | 0.002  (-0.002,  0.006) |
| **NOBR-cancers excluding lung** | 0.590 (0.571,  0.610) | 0.009   (-0.007,  0.025) | 0.582 (0.562,  0.606) | 0.009  (-0.007,  0.026) | 0.582 (0.562,  0.601) | 0.590  (0.571,  0.610) | 0.000  (-0.006,  0.007) |
| **Specific cancer sites** | | | | | | | |
| Colorectal | 0.640 (0.576,  0.712) | 0.009   (-0.026,  0.044) | 0.654 (0.587,  0.728) | -0.028   (-0.062,  0.006) | 0.662 (0.590,  0.744) | 0.640  (0.576,  0.712) | -0.003  (-0.036,  0.030) |
| Pancreas | 0.689 (0.624,  0.761) | -0.001   (-0.003,  0.002) | 0.703 (0.642,  0.769) | -0.000  (-0.034,  0.034) | 0.688 (0.619,  0.764) | 0.689  (0.624,  0.761) | 0.008   (-0.033,  0.050) |
| Kidney | 0.763 (0.734,  0.793) | -0.008   (-0.027,  0.011) | 0.764 (0.735,  0.793) | -0.005  (-0.037,  0.027) | 0.768 (0.740,  0.797) | 0.763  (0.734,  0.793) | -0.015   (-0.073,  0.043) |
| Lung | 0.700 (0.653,  0.750) | 0.000   (-0.002,  0.003) | 0.696 (0.648,  0.748) | 0.004   (-0.003,  0.012) | 0.676 (0.632,  0.723) | 0.700  (0.653,  0.750) | 0.004  (-0.002,  0.010) |
| Endometrial | 0.646 (0.583,  0.714) | 0.010  (-0.019,  0.039) | 0.649 (0.594,  0.709) | 0.017  (-0.016,  0.051) | 0.667 (0.613,  0.724) | 0.646  (0.583,  0.714) | -0.020  (-0.054,  0.014) |
| Ovarian | 0.597 (0.577,  0.617) | 0.002  (-0.026,  0.030) | 0.587 (0.567,  0.608) | -0.007  (-0.065,  0.052) | 0.596 (0.576,  0.617) | 0.597  (0.577,  0.617) | 0.018  (-0.015,  0.051) |
| Post-menopausal breast cancer | 0.575 (0.565,  0.586) | 0.011   (-0.005,  0.026) | 0.580 (0.569,  0.591) | 0.011  (-0.002,  0.024) | 0.580 (0.569,  0.592) | 0.575  (0.565,  0.586) | 0.009   (-0.006,  0.024) |
| **Key: Green – significant difference in C-statistic.**  **Abbreviations**: SE, standard error; OBR, obesity-related; NOBR, non-obesity related; BMI, body mass index; CI, confidence interval; MV, multivariable-adjusted, WC, waist circumference. | | | | | | | |

**Analysis of Black participants**

**Table S37: Summary of the exposure metrics in Black participants only.**

| **Characteristic** | **Men** |
| --- | --- |
| **Baseline BMI, kg/m^2^** | 28.50 (5.00) |
| **Baseline WC, cm** | 101.00 (13.00) |
| **Follow up years** | 12.60 (6.30) |
| **Total cumulative waist circumference years** | 28.00 (58.00) |
| **Total cumulative waist circumference degree** | 14.00 (27.00) |
| **Total cumulative waist circumference duration** | 3.60 (3.99) |
|  | **Women** |
| **Baseline BMI, kg/m^2^** | 32.00 (7.00) |
| **Baseline WC, cm** | 106.00 (17.00) |
| **Follow up years** | 15.60 (5.40) |
| **Total cumulative waist circumference years** | 136.00 (123.00) |
| **Total cumulative waist circumference degree** | 64.00 (55.00) |
| **Total cumulative waist circumference duration** | 7.5 (2.80) |
| Mean (SD)  **Abbreviations**: N = number of participants; SD, standard deviation; BMI, body mass index, WC, waist circumference. | |

**Table S38: Incidence of cancer (events/1000 Person-Years) according to cumulative waist circumference years stratified by biological sex, race, smoking, HRT in Black participants only.**

| **Men** | | | | | | | | | | | | | | |
| --- | --- | --- | --- | --- | --- | --- | --- | --- | --- | --- | --- | --- | --- | --- |
|  | **0 Waist circumference-years** | | | **≤100 Waist circumference-years** | | | **>100 Waist circumference-years** | | | **Baseline cohort** | | | |  |
|  | **N** | **PYFU** | **IR (95% CI)** | **N** | **PYFU** | **IR (95% CI)** | **N** | **PYFU** | **IR (95% CI)** | **N** | **PYFU** | **IR (95% CI)** |  |  |
| **Whole sample** | 760 | 144018.80 | 5.28  (4.9,5.66) | 487 | 91142.26 | 5.34  (4.86,5.82) | 267 | 41240.44 | 6.47  (5.69,7.26) | 1514 | 276401.50 | 5.48  (5.2,5.76) |  |  |
| **Smoking** | | | | | | | | | | | | | | |
| **Ever** | 555 | 98286.49 | 5.65  (5.17,6.12) | 374. | 66675.37 | 5.61  (5.03,6.19) | 195 | 30308.06 | 6.42  (5.50,7.34) | 1124 | 195269.90 | 5.76  (5.42,6.09) |  |  |
| **Never** | 205 | 45732.34 | 4.48   (3.85,5.10) | 113 | 24466.90 | 4.61   (3.74,5.49) | 72 | 10932.38 | 6.62  (5.05,8.20) | 390 | 81131.62 | 4.81  (4.32,5.29) |  |  |
| **Women** | | | | | | | | | | | | | | |
| **Whole sample** | 34 | 13375.32 | 2.54  (1.65,3.44) | 82 | 30509.99 | 2.69  (2.09,3.29) | 194 | 64154.37 | 3.02  (2.59,3.46) | 310 | 108039.70 | 2.87  (2.55,3.19) |  |  |
| **Smoking** | | | | | | | | | | | | | | |
| **Ever** | 16 | 5998.72 | 2.67  (1.26,4.07) | 45 | 12894.86 | 3.49  (2.43,4.55) | 96 | 26079.84 | 3.66  (2.91,4.42) | 157 | 44973.41 | 3.48  (2.92,4.04) |  |  |
| **Never** | 18 | 7376.61 | 2.44  (1.23,3.65) | 37 | 17615.13 | 2.1  (1.39,2.81) | 99 | 38074.53 | 2.59  (2.06,3.11) | 154 | 63066.27 | 2.43   (2.04,2.83) |  |  |
| **HRT** | | | | | | | | | | | | | | |
| **Ever** | 16 | 7396.87 | 2.12  (0.99,3.25) | 24 | 14497.46 | 1.68  (0.97,2.38) | 59 | 22558.78 | 2.61  (1.92,3.3) | 99 | 44453.11 | 2.22  (1.77,2.68) |  |  |
| **Never** | 18 | 5978.46 | 3.06  (1.56,4.56) | 58 | 16012.53 | 3.61  (2.64,4.57) | 135 | 41595.59 | 3.25  (2.69,3.81) | 211 | 63586.57 | 3.32   (2.87,3.78) |  |  |
| **Abbreviations**: N, number of cancer events; PYFR, person-years of follow-up; IR, incidence rate of all cancers | | | | | | | | | | | | | | |

**Table S39: Hazard ratio of specific cancers related to waist-circumference years in Black participants only.**

| **Outcomes** | **Cancers** | **Waist circumference-years (per SD)** | | **Baseline WC (per SD)** | | **Baseline BMI (per SD)** | |
| --- | --- | --- | --- | --- | --- | --- | --- |
|  |  | **Age-adjusted HR**  **(95% CI)** | **MV-adjusted HR**  **(95% CI)** | **Age-adjusted HR (95% CI)** | **MV-adjusted HR**  **(95% CI)** | **Age-adjusted HR**  **(95% CI)** | **MV-adjusted HR**  **(95% CI)** |
|  |  | **Men** | | | | | |
| All Cancers | 301 | 1.09 (0.99,1.20) | 1.08 (0.98,1.19) | 1.02 (0.91,1.14) | 1.02 (0.91,1.14) | 1.02 (0.91,1.14) | 1.02 (0.91,1.14) |
| OBR-cancers | 58 | 1.18 (0.98,1.43) | 1.17 (0.96,1.41) | 1.21 (0.95,1.55) | 1.19 (0.94,1.53) | 1.18 (0.92,1.51) | 1.16 (0.90,1.49) |
| NOBR-cancers | 243 | 1.06 (0.95,1.19) | 1.06 (0.95,1.19) | 0.98 (0.86,1.12) | 0.98 (0.86,1.11) | 0.99 (0.87,1.12) | 0.99 (0.87,1.13) |
| NOBR-cancers excluding lung and prostate | 53 | 1.21 (1.01,1.46) | 1.20 (1.00,1.45) | 1.15 (0.89,1.49) | 1.14 (0.88,1.47) | 1.07 (0.82,1.40) | 1.06 (0.81,1.39) |
| **Specific cancer sites** | | | | | | | |
| Colorectal | 28 | 1.38 (1.14,1.67) | 1.37 (1.13,1.67) | 1.58 (1.19,2.11) | 1.56 (1.17,2.09) | 1.62 (1.21,2.16) | 1.61 (1.19,2.17) |
| Pancreas | 12 | 0.78 (0.33,1.82) | 0.78 (0.33,1.83) | 0.86 (0.46,1.59) | 0.85 (0.45,1.60) | 0.79 (0.41,1.49) | 0.79 (0.41,1.51) |
| Lung | 33 | 1.13 (0.87,1.47) | 1.17 (0.90,1.53) | 0.96 (0.67,1.36) | 1.01 (0.71,1.44) | 0.82 (0.57,1.19) | 0.9 (0.62,1.31) |
| Prostate | 157 | 0.98 (0.83,1.16) | 0.97 (0.82,1.15) | 0.93 (0.80,1.10) | 0.93 (0.79,1.09) | 1.00 (0.85,1.17) | 0.99 (0.84,1.16) |

| **Women** | | | | | | | |
| --- | --- | --- | --- | --- | --- | --- | --- |
| All Cancers | 310 | 1.06 (0.95,1.17) | 1.05 (0.94,1.17) | 1.11 (0.99,1.24) | 1.09 (0.98,1.22) | 1.09 (0.98,1.22) | 1.08 (0.97,1.20) |
| OBR-cancers | 212 | 1.11 (0.98,1.26) | 1.1 (0.96,1.25) | 1.15 (1.01,1.31) | 1.13 (0.99,1.29) | 1.12 (0.99,1.28) | 1.11 (0.98,1.26) |
| NOBR-cancers | 98 | 0.94 (0.76,1.15) | 0.94 (0.77,1.16) | 1.02 (0.84,1.25) | 1.01 (0.83,1.23) | 1.02 (0.83,1.24) | 1.02 (0.84,1.24) |
| NOBR-cancers excluding lung | 70 | 0.96 (0.75,1.21) | 0.96 (0.76,1.22) | 1.07 (0.85,1.35) | 1.07 (0.85,1.34) | 1.07 (0.85,1.35) | 1.07 (0.85,1.34) |
| **Specific cancer sites** | | | | |  |  |  |
| Colorectal | 47 | 1.01 (0.76,1.34) | 1.03 (0.78,1.36) | 0.97 (0.72,1.29) | 0.98 (0.74,1.31) | 0.93 (0.69,1.25) | 0.94 (0.70,1.26) |
| Pancreas | 21 | 1.11 (0.76,1.62) | 1.09 (0.74,1.61) | 1.15 (0.75,1.75) | 1.12 (0.73,1.71) | 0.9 (0.57,1.42) | 0.89 (0.56,1.4) |
| Kidney | 15 | 1.26 (0.76,2.11) | 1.16 (0.69,1.97) | 1.40 (0.88,2.22) | 1.28 (0.80,2.04) | 1.34 (0.87,2.09) | 1.25 (0.80,1.95) |
| Lung | 28 | 0.90 (0.60,1.34) | 0.89 (0.59,1.35) | 0.91 (0.62,1.34) | 0.89 (0.61,1.30) | 0.89 (0.60,1.32) | 0.90 (0.62,1.31) |
| Endometrial | 14 | 1.87 (1.28,2.73) | 1.69 (1.15,2.50) | 1.97 (1.24,3.13) | 1.81 (1.12,2.94) | 1.91 (1.28,2.84) | 1.76 (1.16,2.65) |
| Ovarian | 4 | 0.74 (0.21,2.61) | 0.68 (0.19,2.48) | 1.02 (0.36,2.93) | 0.96 (0.33,2.75) | 1.43 (0.57,3.59) | 1.36 (0.54,3.46) |
| Post-menopausal breast cancer | 90 | 1.03 (0.85,1.26) | 1.03 (0.84,1.25) | 1.12 (0.91,1.37) | 1.10 (0.90,1.35) | 1.07 (0.87,1.31) | 1.06 (0.86,1.29) |
| * Multivariable adjustment for baseline age, race, alcohol, smoking and HRT (in women).  **Abbreviations**: OBR, obesity-related; NOBR, non-obesity related; CI, confidence interval; HR, hazard ratio; BMI, body mass index; MV, multivariable, WC, waist circumference. | | | | | | | |

**Table S40: Hazard ratio of specific cancers related to waist circumference years in Black participants only.**

| **Outcomes** | **Cancers** | **Waist circumference-years**  **(per 100 cm-years)** | | **Baseline WC**  **(per 5 cm)** | | **Baseline BMI**  **(per 5 kg/m2)** | |
| --- | --- | --- | --- | --- | --- | --- | --- |
|  |  | **Age-adjusted HR**  **(95% CI)** | **MV-adjusted HR**  **(95% CI)** | **Age-adjusted HR**  **(95% CI)** | **MV-adjusted HR**  **(95% CI)** | **Age-adjusted HR**  **(95% CI)** | **MV-adjusted HR**  **(95% CI)** |
|  |  | **Men** | | | | | |
| All Cancers | 301 | 1.10 (0.98,1.22) | 1.10 (0.99,1.23) | 1.01 (0.96,1.06) | 1.01 (0.96,1.06) | 1.02 (0.91,1.15) | 1.03 (0.91,1.16) |
| OBR-cancers | 58 | 1.20 (0.97,1.49) | 1.20 (0.97,1.48) | 1.08 (0.98,1.19) | 1.08 (0.98,1.19) | 1.19 (0.92,1.53) | 1.17 (0.90,1.52) |
| NOBR-cancers | 243 | 1.07 (0.95,1.22) | 1.08 (0.95,1.22) | 0.99 (0.94,1.05) | 1.00 (0.95,1.05) | 0.99 (0.87,1.13) | 1.00 (0.87,1.14) |
| NOBR-cancers excluding lung and prostate | 53 | 1.24 (1.01,1.53) | 1.24 (1.01,1.53) | 1.06 (0.96,1.17) | 1.06 (0.95,1.17) | 1.08 (0.82,1.42) | 1.07 (0.81,1.42) |
| **Specific cancer sites** | | | | | | | |
| Colorectal | 28 | 1.43 (1.16,1.78) | 1.42 (1.15,1.77) | 1.20 (1.07,1.35) | 1.2 (1.07,1.34) | 1.65 (1.22,2.23) | 1.65 (1.21,2.24) |
| Pancreas | 12 | 0.76 (0.29,1.95) | 0.77 (0.30,1.99) | 0.94 (0.73,1.21) | 0.94 (0.73,1.22) | 0.78 (0.40,1.52) | 0.79 (0.40,1.56) |
| Lung | 33 | 1.15 (0.85,1.54) | 1.21 (0.91,1.63) | 0.98 (0.85,1.13) | 1.01 (0.87,1.16) | 0.82 (0.56,1.20) | 0.91 (0.62,1.34) |
| Prostate | 157 | 0.98 (0.81,1.18) | 0.98 (0.81,1.18) | 0.97 (0.91,1.04) | 0.97 (0.91,1.04) | 1.00 (0.85,1.18) | 0.99 (0.84,1.18) |

| **Women** | | | | | | | | |
| --- | --- | --- | --- | --- | --- | --- | --- | --- |
| All Cancers | 310 | 1.02 (0.98,1.07) | 1.02 (0.97,1.07) | 1.03 (1.00,1.07) | 1.03 (0.99,1.06) | 1.07 (0.98,1.16) | 1.06 (0.98,1.15) |  |
| OBR-cancers | 212 | 1.05 (0.99,1.11) | 1.04 (0.98,1.10) | 1.04 (1.00,1.08) | 1.04 (1.00,1.08) | 1.09 (0.99,1.20) | 1.08 (0.98,1.19) |  |
| NOBR-cancers | 98 | 0.97 (0.89,1.07) | 0.97 (0.89,1.07) | 1.01 (0.95,1.07) | 1.00 (0.95,1.07) | 1.01 (0.87,1.18) | 1.02 (0.88,1.18) |  |
| NOBR-cancers excluding lung | 70 | 0.98 (0.88,1.09) | 0.98 (0.88,1.09) | 1.02 (0.95,1.09) | 1.02 (0.95,1.09) | 1.05 (0.88,1.25) | 1.05 (0.89,1.25) |  |
| **Specific cancer sites** | | | | | | | | |
| Colorectal | 47 | 1.01 (0.89,1.14) | 1.01 (0.89,1.15) | 0.99 (0.91,1.08) | 0.99 (0.91,1.08) | 0.94 (0.75,1.18) | 0.95 (0.77,1.19) |  |
| Pancreas | 21 | 1.05 (0.88,1.24) | 1.04 (0.88,1.23) | 1.04 (0.92,1.18) | 1.03 (0.91,1.17) | 0.92 (0.65,1.30) | 0.91 (0.65,1.28) |  |
| Kidney | 15 | 1.11 (0.88,1.39) | 1.07 (0.85,1.35) | 1.11 (0.96,1.27) | 1.08 (0.94,1.24) | 1.25 (0.90,1.74) | 1.18 (0.84,1.65) |  |
| Lung | 28 | 0.95 (0.80,1.14) | 0.95 (0.79,1.14) | 0.97 (0.87,1.09) | 0.97 (0.86,1.08) | 0.91 (0.68,1.23) | 0.92 (0.70,1.23) |  |
| Endometrial | 14 | 1.32 (1.12,1.56) | 1.26 (1.06,1.50) | 1.23 (1.07,1.41) | 1.19 (1.03,1.38) | 1.62 (1.21,2.19) | 1.52 (1.12,2.07) |  |
| Ovarian | 4 | 0.87 (0.50,1.53) | 0.84 (0.47,1.50) | 1.01 (0.73,1.38) | 0.99 (0.72,1.35) | 1.31 (0.66,2.61) | 1.26 (0.63,2.53) |  |
| Post-menopausal breast cancer | 90 | 1.02 (0.93,1.11) | 1.01 (0.92,1.11) | 1.03 (0.97,1.10) | 1.03 (0.97,1.09) | 1.05 (0.90,1.22) | 1.04 (0.90,1.21) |  |
| * Multivariable adjustment for baseline age, race, alcohol, smoking and HRT (in women).  **Abbreviations**: OBR, obesity-related; NOBR, non-obesity related; CI, confidence interval; HR, hazard ratio; BMI, body mass index; MV, multivariable, WC, waist circumference. | | | | | | | | |

**Table S41: Comparison of the waist circumference degree and duration per unit standard deviation in Black participants only.**

| **Outcomes** |  | **Cumulative degree of excess WC (per SD)** | | **Cumulative duration of excess WC (per SD)** | |
| --- | --- | --- | --- | --- | --- |
|  |  | **Age-adjusted HR (95% CI)** | **MV-adjusted HR (95% CI)** | **Age-adjusted HR (95% CI)** | **MV-adjusted HR (95% CI)** |
|  | **Cancers** | **Men** | | | |
| All Cancers | 301 | 1.17 (1.01,1.35) | 1.17 (1.00,1.35) | 0.98 (0.83,1.17) | 0.99 (0.83,1.17) |
| OBR-cancers | 58 | 1.11 (0.80,1.54) | 1.11 (0.80,1.55) | 0.91 (0.64,1.30) | 0.91 (0.64,1.30) |
| NOBR-cancers | 243 | 1.17 (0.99,1.39) | 1.17 (0.99,1.39) | 1 (0.82,1.21) | 1.00 (0.83,1.22) |
| NOBR-cancers excluding lung and prostate | 53 | 1.3 (0.95,1.78) | 1.3 (0.95,1.78) | 1.00 (0.67,1.50) | 0.99 (0.66,1.49) |
| **Specific cancer sites** | | | | | |
| Colorectal | 28 | 1.14 (0.75,1.74) | 1.14 (0.74,1.75) | 0.95 (0.60,1.53) | 0.97 (0.61,1.54) |
| Pancreas | 12 | 0.81 (0.27,2.46) | 0.82 (0.27,2.49) | 1.17 (0.55,2.49) | 1.18 (0.55,2.51) |
| Lung | 33 | 0.36 (0.06,2.28) | 0.37 (0.06,2.44) | 0.57 (0.19,1.73) | 0.57 (0.18,1.78) |
| Prostate | 157 | 1.38 (0.93,2.05) | 1.37 (0.94,2.01) | 1.12 (0.69,1.82) | 1.12 (0.69,1.83) |

| **Women** | | | | | |
| --- | --- | --- | --- | --- | --- |
| All Cancers | 310 | 0.90 (0.72,1.11) | 0.91 (0.73,1.13) | 0.95 (0.81,1.12) | 0.96 (0.82,1.13) |
| OBR-cancers | 212 | 0.97 (0.75,1.25) | 0.98 (0.76,1.26) | 1.04 (0.85,1.28) | 1.05 (0.85,1.29) |
| NOBR-cancers | 98 | 0.74 (0.49,1.11) | 0.77 (0.51,1.16) | 0.8 (0.61,1.06) | 0.83 (0.63,1.08) |
| NOBR-cancers excluding lung | 70 | 0.68 (0.42,1.1) | 0.69 (0.43,1.13) | 0.89 (0.64,1.24) | 0.92 (0.66,1.28) |
| **Specific cancer sites** | | | | | |
| Colorectal | 47 | 1.15 (0.68,1.94) | 1.18 (0.7,1.98) | 1.21 (0.79,1.86) | 1.26 (0.82,1.95) |
| Pancreas | 21 | 0.97 (0.46,2.07) | 0.99 (0.46,2.12) | 1.18 (0.64,2.17) | 1.18 (0.64,2.18) |
| Lung | 28 | 0.69 (0.22,2.17) | 0.67 (0.20,2.19) | 14.54 (0.46,456.33) | 13.76 (0.44,428.12) |
| Endometrial | 14 | 0.91 (0.43,1.95) | 0.99 (0.45,2.16) | 0.63 (0.38,1.04) | 0.64 (0.39,1.04) |
| Ovarian | 4 | 1.60 (0.75,3.39) | 1.44 (0.68,3.06) | 1.53 (0.42,5.61) | 1.37 (0.40,4.74) |
| Post-menopausal breast cancer | 90 | 0.24 (0.01,4.11) | 0.21 (0.01,4.13) | 0.47 (0.12,1.82) | 0.45 (0.12,1.75) |
| * Multivariable adjustment for baseline age, baseline WC, race, alcohol, smoking and HRT (in women).  * Degree of excess WC is the cumulative sum of the number of WC units ≥ 102 cm in men and ≥ 88 cm in women over the exposure period.  * Duration of excess WC is the cumulative sum of the duration of WC above the threshold over the exposure period.  **Abbreviations**: OBR, obesity-related; NOBR, non-obesity related; CI, confidence interval; HR, hazard ratio; BMI, body mass index; MV, multivariable, WC, waist circumference. | | | | | |

**Table S42: Comparison of the waist circumference degree and duration per 10 units and per 10 years respectively in Black participants only.**

| **Outcome** | **Cumulative degree of excess WC (per 10 cm)** | | | **Cumulative duration of excess WC (per 10 years)** | | |
| --- | --- | --- | --- | --- | --- | --- |
|  | **Age-adjusted HR**  **(95% CI)** | **MV-adjusted HR**  **(95% CI)** | | **Age-adjusted HR**  **(95% CI)** | **MV-adjusted HR**  **(95% CI)** | |
| **Men** | | | | | | |
| **All Cancers** | 1.02 (1.00,1.03) | 1.02 (1.00,1.03) | | 0.98 (0.77,1.24) | 0.98 (0.77,1.25) | |
| **OBR-cancers** | 1.01 (0.98,1.05) | 1.01 (0.98,1.05) | | 0.87 (0.53,1.44) | 0.88 (0.53,1.45) | |
| **NOBR-cancers** | 1.02 (1.00,1.03) | 1.02 (1.00,1.03) | | 1.00 (0.76,1.31) | 1.01 (0.77,1.32) | |
| **NOBR-cancers excluding lung and prostate** | 1.03 (0.99,1.06) | 1.03 (0.99,1.06) | | 1.00 (0.57,1.76) | 0.99 (0.56,1.75) | |
| **Specific cancer sites** | | | | | | |
| Colorectal | 1.01 (0.97,1.06) | 1.01 (0.97,1.06) | | 0.94 (0.49,1.81) | 0.95 (0.50,1.84) | |
| Pancreas | 0.98 (0.87,1.10) | 0.98 (0.87,1.10) | | 1.25 (0.44,3.58) | 1.25 (0.43,3.63) | |
| Lung | 1.03 (0.99,1.08) | 1.03 (0.99,1.08) | | 1.18 (0.60,2.32) | 1.18 (0.59,2.33) | |
| Prostate | 1.01 (0.98,1.03) | 1.01 (0.98,1.03) | | 0.96 (0.68,1.35) | 0.97 (0.69,1.37) | |
| **Women** | | | | | | |
| **All Cancers** | 1.00 (0.99,1.00) | | 1.00 (0.99,1.01) | 0.93 (0.75,1.16) | | 0.95 (0.76,1.18) |
| **OBR-cancers** | 1.00 (0.99,1.01) | | 1.00 (0.99,1.01) | 1.05 (0.80,1.39) | | 1.06 (0.81,1.40) |
| **NOBR-cancers** | 0.99 (0.97,1.00) | | 0.99 (0.97,1.01) | 0.75 (0.52,1.08) | | 0.78 (0.54,1.11) |
| **NOBR-cancers excluding lung** | 0.98 (0.96,1.00) | | 0.98 (0.96,1.01) | 0.86 (0.56,1.33) | | 0.90 (0.58,1.39) |
| **Specific cancer sites** | | | | | | |
| Colorectal | 1.01 (0.98,1.03) | | 1.01 (0.99,1.03) | 1.29 (0.73,2.28) | | 1.37 (0.77,2.42) |
| Pancreas | 1.00 (0.97,1.03) | | 1.00 (0.97,1.03) | 1.24 (0.55,2.80) | | 1.25 (0.56,2.81) |
| Lung | 0.98 (0.94,1.03) | | 0.98 (0.94,1.03) | 35.08 (0.36,3418.61) | | 32.58 (0.34,3141.1) |
| Endometrial | 1.00 (0.97,1.03) | | 1.00 (0.97,1.03) | 0.54 (0.28,1.05) | | 0.55 (0.29,1.06) |
| Ovarian | 1.02 (0.99,1.05) | | 1.02 (0.98,1.05) | 1.76 (0.31,9.88) | | 1.52 (0.29,7.92) |
| Post-menopausal breast cancer | 0.94 (0.84,1.06) | | 0.94 (0.83,1.06) | 0.37 (0.06,2.22) | | 0.35 (0.06,2.1) |
| * Multivariable adjustment for baseline age, baseline WC, race, alcohol, smoking and HRT (in women).  * Degree of excess WC is the cumulative sum of the number of WC units ≥ 102 cm in men and ≥ 88 cm in women over the exposure period.  * Duration of excess WC is the cumulative sum of the duration of WC above the threshold over the exposure period.  **Abbreviations**: OBR, obesity-related; NOBR, non-obesity related; CI, confidence interval; HR, hazard ratio; BMI, body mass index; MV, multivariable, WC, waist circumference. | | | | | | |

**Table S43: Comparison of the metrics by Akaike information criterion in Black participants only.**

|  |  | |  | |  | | **AIC** | | | | |
| --- | --- | --- | --- | --- | --- | --- | --- | --- | --- | --- | --- |
| **Characteristic** | **MV-adjusted waist circumference-years** | **MV-adjusted single**  **WC** | | **MV-adjusted single BMI** | | **MV-adjusted waist circumference -years with single WC** | | **MV-adjusted waist circumference -years with single BMI** | **MV-adjusted single WC -years with single BMI** | **MV-adjusted cumulative degree of excess WC** | **MV-adjusted cumulative duration of excess WC** |
| **Men** | | | | | | | | | | | |
| **All cancers** | 3807.01 | 3809.51 | | 3809.50 | | 3807.97 | | 3807.68 | 3811.5 | 3807.11 | 3809.64 |
| **OBR-cancers** | 719.67 | 719.78 | | 720.50 | | 721.66 | | 721.44 | 721.60 | 719.60 | 721.34 |
| **NOBR-cancers** | 3079.35 | 3080.60 | | 3080.63 | | 3080.07 | | 3079.32 | 3082.51 | 3079.46 | 3080.62 |
| **NOBR-cancers excluding lung and prostate** | 681.49 | 683.57 | | 684.39 | | 682.18 | | 683.26 | 684.16 | 681.50 | 684.07 |
| **Specific cancer sites** | | | | | | | | | | | |
| Colorectal | 352.58 | 351.81 | | 351.09 | | 352.80 | | 353.46 | 353.04 | 352.52 | 355.98 |
| Pancreas | 146.47 | 146.65 | | 146.37 | | 148.32 | | 148.47 | 148.15 | 146.52 | 146.84 |
| Lung | 412.04 | 413.36 | | 413.14 | | 411.13 | | 413.15 | 413.04 | 412.08 | 413.22 |
| Prostate | 1996.30 | 1995.71 | | 1996.36 | | 1998.28 | | 1997.47 | 1994.69 | 1996.28 | 1995.86 |
| **Women** | | | | | | | | | | | |
| **All cancers** | 4368.87 | 4367.27 | | 4367.56 | | 4369.26 | | 4369.26 | 4369.24 | 4368.81 | 4369.22 |
| **OBR-cancers** | 3002.46 | 3001.35 | | 3001.94 | | 3003.90 | | 3003.90 | 3003.35 | 3002.41 | 3002.37 |
| **NOBR-cancers** | 1371.38 | 1371.70 | | 1371.68 | | 1372.01 | | 1372.01 | 1373.66 | 1371.42 | 1370.79 |
| **NOBR-cancers excluding lung** | 993.54 | 993.37 | | 993.32 | | 993.39 | | 993.39 | 995.32 | 993.56 | 993.66 |
| **Specific cancer sites** | | | | | | | | | | | |
| Colorectal | 671.29 | 671.32 | | 671.16 | | 672.32 | | 672.32 | 672.89 | 671.29 | 670.77 |
| Pancreas | 297.48 | 297.41 | | 297.39 | | 297.44 | | 297.44 | 294.92 | 297.49 | 297.11 |
| Kidney | 223.71 | 222.97 | | 223.12 | | 224.95 | | 224.95 | 224.96 | 223.69 | 216.63 |
| Lung | 386.11 | 386.04 | | 386.11 | | 388.08 | | 388.08 | 388.04 | 386.13 | 383.35 |
| Endometrial | 195.65 | 196.17 | | 195.47 | | 196.95 | | 196.95 | 197.37 | 195.58 | 199.01 |
| Ovarian | 57.30 | 57.69 | | 57.31 | | 55.24 | | 55.24 | 57.76 | 57.34 | 56.88 |
| Post-menopausal breast cancer | 1287.26 | 1286.49 | | 1287.02 | | 1288.91 | | 1288.91 | 1288.16 | 1287.25 | 1286.94 |
| * Multivariable adjustment for baseline age, race, alcohol, smoking and HRT (in women).  **Abbreviations**: WC, waist circumference, SE, standard error; OBR, obesity-related; NOBR, non-obesity related; BMI, body mass index; AIC, Akaike information criterion. | | | | | | | | | | | |

| **Table S44a and S44b: Comparison of the waist circumference-years metric, single WC and single BMI each at Visit 4 using Harrell’s C-statistic in Black participants only.** | | | | | | | | | |
| --- | --- | --- | --- | --- | --- | --- | --- | --- | --- |
| 1. **Harrell’s C-statistic (95% CI)** | | | | | | | | | |
| **Characteristic** | **WC-years** | **Single WC** | **Difference in c-statistic between single WC vs WC-years** | **Single**  **BMI** | **Difference in c-statistic between**  **single BMI vs WC-years** | **Difference in c-statistic between single BMI vs single WC** | **WC-years with single WC** | **Difference in c-statistic between WC-years with**  **single WC and WC-years** |  |
| **Men** | | | | | | | | | |
| **All cancers** | 0.588   (0.568,  0.608) | 0.5823  (0.563,  0.603) | -0.005  (-0.017,  0.007) | 0.583  (0.563,  0.604) | -0.005   (-0.016,  0.007) | 0.001   (-0.001,  0.002) | 0.585  (0.565,  0.606) | -0.001   (-0.011,  0.009) |  |
| **OBR-cancers** | 0.701  (0.656,  0.749) | 0.708  (0.667,  0.753) | 0.008  (-0.034,  0.029) | 0.701  (0.660,  0.746) | 0.001  (-0.019,  0.020) | -0.007   (-0.019,  0.004) | 0.703  (0.660,  0.748) | 0.007  (-0.006,  0.020) |  |
| **NOBR-cancers** | 0.571  (0.549,  0.594) | 0.573  (0.551,  0.596) | 0.003  (-0.01,  0.015) | 0.573   (0.551,  0.596) | 0.002   (-0.009,  0.013) | -0.001   (-0.003,  0.002) | 0.571  (0.548,  0.594) | 0.001   (-0.015,  0.018) |  |
| **NOBR-cancers excluding lung and prostate** | 0.608  (0.563,  0.658) | 0.593  (0.548,  0.641) | -0.016  (-0.048,  0.017) | 0.596   (0.553,  0.643) | -0.012  (-0.042,  0.018) | 0.004  (-0.025,  0.033) | 0.621  (0.576,  0.671) | 0.001  (-0.025,  0.027) |  |
| **Specific cancer sites** | | | | | | | | | |
| Colorectal | 0.679  (0.610,  0.756) | 0.694  (0.620,  0.776) | 0.015  (-0.037,  0.066) | 0.699  (0.628,  0.779) | 0.020  (-0.033,  0.073) | 0.005   (-0.017,  0.028) | 0.698 (0.626,  0.779) | 0.013   (-0.029,  0.055) |  |
| Kidney | 0.786  (0.691,  0.895) | 0.770  (0.684,  0.867) | -0.016  (-0.101,  0.068) | 0.787  (0.707,  0.877) | 0.001   (-0.050,  0.052) | 0.017  (-0.032,  0.067) | 0.776 (0.645,  0.933) | -0.028  (-0.162,  0.107) |  |
| Lung | 0.734 (0.677,  0.797) | 0.741 (0.686,  0.800) | 0.006  (-0.019,  0.038) | 0.758  (0.706,  0.814) | 0.024  (-0.026,  0.073) | 0.017  (-0.014,  0.049) | 0.771 (0.710,  0.838) | 0.015  (-0.023,  0.054) |  |
| Prostate | 0.593 (0.566,  0.623) | 0.585 (0.557,  0.615) | -0.008  (-0.019,  0.003) | 0.592  (0.564,  0.622) | -0.001  (-0.005,  0.003) | 0.007  (-0.006,  0.020) | 0.596 (0.568,  0.627) | -0.006  (-0.021,  0.009) |  |
| **Women** | | | | | | | | | |
| **All cancers** | 0.609  (0.590,  0.628) | 0.611  (0.592,  0.631) | 0.003   (-0.003,  0.008) | 0.612  (0.593,  0.632) | 0.004  (-0.002,  0.009) | 0.001   (-0.003,  0.005) | 0.614 (0.595,  0.633) | 0.004   (-0.005,  0.013) |  |
| **OBR-cancers** | 0.594  (0.572,  0.617) | 0.597  (0.575,  0.620) | 0.003   (-0.005,  0.012) | 0.598  (0.576,  0.621) | 0.004  (-0.005,  0.013) | 0.001  (-0.007,  0.008) | 0.597  (0.575,  0.620) | 0.003  (-0.007,  0.013) |  |
| **NOBR-cancers** | 0.662  (0.626,  0.701) | 0.666  (0.630,  0.704) | 0.004   (-0.006,  0.014) | 0.667  (0.631,  0.705) | 0.005   (-0.006,  0.016) | 0.001  (-0.002,  0.004) | 0.668  (0.631,  0.706) | 0.005  (-0.009,  0.020) |  |
| **NOBR-cancers excluding lung** | 0.621  (0.581,  0.665) | 0.640  (0.601,  0.681) | 0.018  (-0.017,  0.053) | 0.643  (0.604,  0.685) | 0.022  (-0.013,  0.056) | 0.004  (-0.007,  0.014) | 0.647  (0.606,  0.690) | 0.026  (-0.018,  0.069) |  |
| **Specific cancer sites** | | | | | | | | | |
| Colorectal | 0.642  (0.592,  0.696) | 0.644  (0.595,  0.696) | 0.002  (-0.014,  0.017) | 0.645  (0.597,  0.697) | 0.003  (-0.027,  0.033) | 0.001  (-0.016,  0.019) | 0.655  (0.606,  0.707) | 0.007  (-0.019,  0.033) |  |
| Pancreas | 0.735  (0.658,  0.820) | 0.737  (0.662,  0.820) | 0.002   (-0.006,  0.010) | 0.734  (0.657,  0.819) | -0.001  (-0.026,  0.024) | -0.003   (-0.028,  0.022) | 0.740  (0.665,  0.824) | 0.002  (-0.005,  0.009) |  |
| Kidney | 0.711  (0.634,  0.798) | 0.730  (0.654,  0.816) | 0.019  (-0.019,  0.057) | 0.725  (0.650,  0.809) | 0.014  (-0.014,  0.043) | -0.005  (-0.026,  0.016) | 0.719  (0.639,  0.810) | 0.017   (-0.049,  0.082) |  |
| Lung | 0.770 (0.703,  0.845) | 0.771 (0.704,  0.845) | 0.001  (-0.009,  0.011) | 0.770  (0.703,  0.843) | -0.001   (-0.009,  0.008) | -0.001  (-0.007,  0.004) | 0.770  (0.703,  0.844) | 0.001  (-0.007,  0.008) |  |
| Endometrial | 0.860  (0.809,  0.914) | 0.863  (0.813,  0.916) | 0.004  (-0.029,  0.036) | 0.872  (0.824,  0.924) | 0.013  (-0.019,  0.044) | 0.009   (-0.013,  0.031) | 0.870 (0.821,  0.923) | 0.005  (-0.012,  0.021) |  |

| 1. **Harrell’s C-statistic (95% CI)** | | | | | | | |
| --- | --- | --- | --- | --- | --- | --- | --- |
| **Characteristic** | **WC -years with single BMI** | **Difference in c-statistic between WC-years with**  **single BMI vs WC-years** | **Single WC with single BMI** | **Difference in c-statistic between**  **single BMI with single WC and WC-years** | **Cumulative degree of excess WC** | **Cumulative duration of excess WC** | **Difference in c-statistic between**  **cumulative WC duration and WC degree** |
| **All cancers** | 0.585   (0.565,  0.606) | -0.003  (-0.012,  0.006) | 0.583  (0.563,  0.604) | -0.005  (-0.016,  0.007) | 0.589  (0.568,  0.608) | 0.582  (0.562,  0.603) | -0.006  (-0.018,  0.007) |
| **OBR-cancers** | 0.703  (0.659,  0.750) | 0.002   (-0.002,  0.006) | 0.701   (0.660,  0.746) | 0.007   (-0.016,  0.031) | 0.702   (0.658,  0.750) | 0.688   (0.645,  0.734) | -0.014   (-0.040,  0.013) |
| **NOBR-cancers** | 0.571  (0.548,  0.595) | 0.000  (-0.014,  0.014) | 0.573   (0.551,  0.596) | 0.004   (-0.010,  0.017) | 0.571   (0.549,  0.594) | 0.573   (0.551,  0.596) | 0.003   (-0.009,  0.014) |
| **NOBR-cancers excluding lung and prostate** | 0.621   (0.577,0.669) | 0.013   (-0.038,  0.065) | 0.596  (0.551,  0.645) | 0.005   (-0.062,  0.072) | 0.608  (0.562,  0.657) | 0.597  (0.551,  0.646) | -0.011  (-0.040,  0.019) |
| **Specific cancer sites** | | | | | | | |
| Colorectal | 0.698 (0.628,  0.777) | 0.019  (-0.025,  0.064) | 0.699 (0.627,  0.779) | 0.020   (-0.034,  0.073) | 0.680 (0.610,  0.756) | 0.665  (0.595,  0.743) | -0.015  (-0.054,  0.025) |
| Kidney | 0.776 (0.665,  0.905) | -0.010  (-0.128,  0.107) | 0.787 (0.694,  0.893) | -0.023  (-0.072,  0.025) | 0.784 (0.690,  0.892) | 0.776  (0.687,  0.878) | -0.008  (-0.037,  0.021 |
| Lung | 0.771 (0.715,  0.832) | 0.037  (-0.017,  0.090) | 0.758 (0.709,  0.810) | 0.036  (-0.022,  0.093) | 0.735 (0.677,  0.797) | 0.738  (0.681,  0.800) | 0.003  (-0.004,  0.011) |
| Prostate | 0.596 (0.569,  0.625) | 0.003  (-0.001,  0.006) | 0.592  (0.565,  0.621) | 0.007  (-0.018,  0.031) | 0.593 (0.565,  0.622) | 0.589  (0.561,  0.618) | -0.004  (-0.014,  0.006) |
| **Women** | | | | | | | |
| **All cancers** | 0.614 (0.595,  0.633) | 0.005  (-0.003,  0.013) | 0.612 (0.593,  0.632) | 0.003   (-0.002,  0.008) | 0.609 (0.590,  0.628) | 0.605  (0.587,  0.625) | -0.003  (-0.009,  0.003) |
| **OBR-cancers** | 0.597 (0.575,  0.620) | 0.003  (-0.004,  0.010) | 0.598 (0.575,  0.621) | 0.003   (-0.005,  0.012) | 0.594 (0.572,  0.617) | 0.595  (0.572,  0.618) | 0.001   (-0.010,  0.012) |
| **NOBR-cancers** | 0.668 (0.631,  0.707) | 0.006   (-0.008,  0.019) | 0.667 (0.631,  0.705) | 0.004  (-0.006,  0.015) | 0.662 (0.626,  0.701) | 0.661  (0.625,  0.700) | -0.001   (-0.009,  0.007) |
| **NOBR-cancers excluding lung** | 0.647 (0.605,  0.691) | 0.025  (-0.013,  0.063) | 0.643 (0.604,  0.684) | 0.022  (-0.013,  0.056) | 0.622 (0.581,  0.665) | 0.628  (0.588,  0.671) | 0.007   (-0.010,  0.023) |
| **Specific cancer sites** | | | | | | | |
| Colorectal | 0.655 (0.607,  0.706) | 0.013  (-0.030,  0.055) | 0.648 (0.597,  0.697) | 0.005  (-0.029,  0.039) | 0.642 (0.592,  0.696) | 0.647  (0.596,  0.702) | 0.005  (-0.024,  0.033) |
| Pancreas | 0.740 (0.662,  0.827) | 0.006  (-0.034,  0.045) | 0.734 (0.657,  0.820) | 0.010   (-0.062,  0.082) | 0.734 (0.658,  0.820) | 0.738  (0.658,  0.828) | 0.004   (-0.013,  0.020) |
| Kidney | 0.719 (0.642,  0.806) | 0.008  (-0.038,  0.054) | 0.725 (0.650,  0.810) | 0.020  (-0.017,  0.057) | 0.712 (0.635,  0.798) | 0.774  (0.720,  0.831) | 0.062   (-0.005,  0.128) |
| Lung | 0.770 (0.703,  0.844) | 0.000   (-0.005,  0.005) | 0.770 (0.703,  0.843) | 0.001   (-0.009,  0.010) | 0.770 (0.703,  0.844) | 0.785  (0.725,  0.850) | 0.015  (-0.014,  0.043) |
| Endometrial | 0.870 (0.822,  0.921) | 0.011  (-0.009,  0.030) | 0.872 (0.824,  0.924) | 0.011  (-0.020,  0.043) | 0.861 (0.810,  0.914) | 0.842  (0.790,  0.897) | -0.019  (-0.065,  0.027) |
| **Key: Green – significant difference in C-statistic.**  **Abbreviations**: WC, waist circumference, SE, standard error; OBR, obesity-related; NOBR, non-obesity related; BMI, body mass index; CI, confidence interval; MV, multivariable-adjusted. | | | | | | | |
